# Tuberculosis drugs’ distribution and emergence of resistance in patient’s lung lesions: A mechanistic model and tool for regimen and dose optimization

**DOI:** 10.1371/journal.pmed.1002773

**Published:** 2019-04-02

**Authors:** Natasha Strydom, Sneha V. Gupta, William S. Fox, Laura E. Via, Hyeeun Bang, Myungsun Lee, Seokyong Eum, TaeSun Shim, Clifton E. Barry, Matthew Zimmerman, Véronique Dartois, Radojka M. Savic

**Affiliations:** 1 Department of Bioengineering and Therapeutic Sciences, University of California, San Francisco, San Francisco, California, United States of America; 2 Tuberculosis Research Section, Laboratory of Clinical Immunology and Microbiology, NIAID, NIH, Bethesda, Maryland, United States of America; 3 International Tuberculosis Research Center, Changwon, Republic of Korea; 4 Asan Medical Center, Seoul, Republic of Korea; 5 Public Health Research Institute and New Jersey Medical School, Rutgers, The State University of New Jersey, Newark, New Jersey, United States of America; Harvard School of Public Health, UNITED STATES

## Abstract

**Background:**

The sites of mycobacterial infection in the lungs of tuberculosis (TB) patients have complex structures and poor vascularization, which obstructs drug distribution to these hard-to-reach and hard-to-treat disease sites, further leading to suboptimal drug concentrations, resulting in compromised TB treatment response and resistance development. Quantifying lesion-specific drug uptake and pharmacokinetics (PKs) in TB patients is necessary to optimize treatment regimens at all infection sites, to identify patients at risk, to improve existing regimens, and to advance development of novel regimens. Using drug-level data in plasma and from 9 distinct pulmonary lesion types (vascular, avascular, and mixed) obtained from 15 hard-to-treat TB patients who failed TB treatments and therefore underwent lung resection surgery, we quantified the distribution and the penetration of 7 major TB drugs at these sites, and we provide novel tools for treatment optimization.

**Methods and findings:**

A total of 329 plasma- and 1,362 tissue-specific drug concentrations from 9 distinct lung lesion types were obtained according to optimal PK sampling schema from 15 patients (10 men, 5 women, aged 23 to 58) undergoing lung resection surgery (clinical study NCT00816426 performed in South Korea between 9 June 2010 and 24 June 2014). Seven major TB drugs (rifampin [RIF], isoniazid [INH], linezolid [LZD], moxifloxacin [MFX], clofazimine [CFZ], pyrazinamide [PZA], and kanamycin [KAN]) were quantified. We developed and evaluated a site-of-action mechanistic PK model using nonlinear mixed effects methodology. We quantified population- and patient-specific lesion/plasma ratios (RPLs), dynamics, and variability of drug uptake into each lesion for each drug. CFZ and MFX had higher drug exposures in lesions compared to plasma (median RPL 2.37, range across lesions 1.26–22.03); RIF, PZA, and LZD showed moderate yet suboptimal lesion penetration (median RPL 0.61, range 0.21–2.4), while INH and KAN showed poor tissue penetration (median RPL 0.4, range 0.03–0.73). Stochastic PK/pharmacodynamic (PD) simulations were carried out to evaluate current regimen combinations and dosing guidelines in distinct patient strata. Patients receiving standard doses of RIF and INH, who are of the lower range of exposure distribution, spent substantial periods (>12 h/d) below effective concentrations in hard-to-treat lesions, such as caseous lesions and cavities. Standard doses of INH (300 mg) and KAN (1,000 mg) did not reach therapeutic thresholds in most lesions for a majority of the population. Drugs and doses that did reach target exposure in most subjects include 400 mg MFX and 100 mg CFZ. Patients with cavitary lesions, irrespective of drug choice, have an increased likelihood of subtherapeutic concentrations, leading to a higher risk of resistance acquisition while on treatment. A limitation of this study was the small sample size of 15 patients, performed in a unique study population of TB patients who failed treatment and underwent lung resection surgery. These results still need further exploration and validation in larger and more diverse cohorts.

**Conclusions:**

Our results suggest that the ability to reach and maintain therapeutic concentrations is both lesion and drug specific, indicating that stratifying patients based on disease extent, lesion types, and individual drug-susceptibility profiles may eventually be useful for guiding the selection of patient-tailored drug regimens and may lead to improved TB treatment outcomes. We provide a web-based tool to further explore this model and results at http://saviclab.org/tb-lesion/.

## Abstract 2

Using data from resected lung lesions, Radojka Savic and colleagues develop a model to describe the amount and rate of drug penetration for seven major tuberculosis drugs into lung tissue.

## Author summary

**Why was this study done?:**

- Tuberculosis (TB) remains the number one infectious disease killer, with patients failing treatment despite use of multidrug curative treatment.
- The potential reason for suboptimal response is reduced drug penetration to the diverse lung lesions formed by the host immune system, in which the bacteria remains in the secluded areas inaccessible to the drug.

**What did the researchers do and find?:**

- We developed a mathematical model for 7 major TB drugs by utilizing actual patient data, which describes the amount and rate of drug penetration into 9 distinct TB lesions in which mycobacteria reside.
- The majority of TB drugs show poor penetration into the cavitary lesions, leading to inadequate drug levels at the site of the disease.
- Multidrug-level simulations to identify favorable drug combinations for cure at the site of infection showed that a combination of clofazimine (CFZ) and moxifloxacin (MFX) or CFZ and linezolid (LZD) would be optimal for patients presenting with caseous lesions.

**What do these findings mean?:**

- Drugs do not penetrate equally in different tissues, and considering that a variety of TB patients presented with unique lesion subtypes and lung pathology, the current one regimen to fit all approaches is inadequate to treat every patient.
- To maximize cure, a patient-focused approach is needed for which clinical phenotype should be used to match the right drug regimen to the right patient.

## Introduction

A primary objective of contemporary tuberculosis (TB) drug development programs is the identification of therapeutic regimens that have shorter treatment duration and improved efficacy with decreased risk of resistance [1]. Similarly, therapeutic use of the current regimen for drug-sensitive (DS)-TB can further be optimized, as its current use, despite high efficacy of the regimens (>95%) in the clinical trials, leads to unsatisfying effectiveness in the field and emergence of resistance [2–4]. Further, both efficacy and effectiveness of regimens for all forms of drug-resistant TB can further be optimized to reach high cure rates. A significant barrier to improving TB regimens is the complex pathology of TB in human lung tissue. Drug access to the site of mycobacterial infection becomes increasingly difficult as the disease progresses from initial macrophage infection to the formation of heterogeneous granuloma lesions that have variable or limited blood supply [5,6]. In a clinical research study of drug distribution into TB lesions [7], imaging and conventional mass spectrometry of resected lung lesions from patients with multidrug-resistant (MDR)-TB showed that the distribution patterns of TB drugs within these diverse lesions vary significantly and that, in many cases, drug concentrations in lesions correlate poorly with concentrations measured in blood [7,8]. Presence of cavitary lesions and extensive lung pathology has been established as a major risk factor for poor TB outcomes, which could be explained by reduced drug penetration at these sites. Recent translational studies of rifapentine’s poor penetration properties in the cavitary lesions quantitatively predicted and explained substantially longer times of culture conversion observed in patients with large cavitation and comparably high blood exposures [9,10]. Drug regimens designed solely based on plasma pharmacokinetic (PK) profiles are therefore inadequate to predict the true sterilizing effect of drugs at the site of mycobacterial infection, especially for the drugs that have reduced or variable penetration at the site of infection. Additionally, mycobacterial resistance may be driven by this differential drug exposure in infected TB lesions, for which windows of monotherapy at selected times and locations likely increase drug pressure. Our goal is to provide a data-driven framework and tool that enables quantification of lesion-specific information in addition to dosing regimens and microbiological PK/pharmacodynamic (PD) parameters and can serve the TB community at multiple levels, such as a) treatment optimization to achieve cure, b) profiling high-risk populations based on reduced penetration in hard-to-treat lesions with the current regimens, and c) in silico investigation and optimization of novel regimens that achieve sterilization and prevent resistance development.

Here, (1) to our knowledge, we report for the first time the penetration of kanamycin (KAN) and linezolid (LZD) in human TB lesions; (2) we report a population lesion-focused PK model and parameters from the observed concentration–time profiles in TB patients of first-line drugs isoniazid (INH), rifampicin (RIF), and pyrazinamide (PZA) and second-line drugs moxifloxacin (MFX), KAN, clofazimine (CFZ), and LZD in 9 distinct human lesion-specific tissues: uninvolved lung, closed nodules (cellular, necrotic, or fibrotic), cavity wall, cavity caseum or nodule caseum, and fungal ball; (3) we profile by means of stochastic population simulations the clinical utility of these 7 drugs within a regimen with a focus on identifying underexposed lesions, suboptimal doses and regimens, and patient subgroups at risk of relapse and resistance development; and (4) we provide a tool that can be used by scientific and clinical communities to evaluate the lesion-focused time course of drug levels following various drug combinations, doses, and schedules.

## Methods

### Patient, drug regimen, and surgical procedure details

Original clinical trial design and partial raw data on RIF, INH, PZA, CFZ, and MFX in plasma, lung tissue, and lesions of TB patients were previously reported in Prideaux and colleagues, and these data were added to our database; the original prospective protocol is included as S1 Study protocol [7]. Briefly, 15 patients who failed initial TB treatment and who were receiving selected first- and second-line TB drugs underwent elective lung resection surgery to debulk tubercular lung disease at the National Masan TB Hospital and Asan and Samsung Medical Center in Seoul and Pusan National University Hospital in Pusan, Republic of Korea. The study was approved by the Institutional Review Board of the National Institute of Allergy and Infectious Diseases and by the institutional review boards of the hospitals conducting the study in the Republic of Korea. The subjects gave written informed consent to participate in the study and to have their resected tissue used for research. Exclusion criteria included patients younger than 20, women pregnant or unwilling to avoid pregnancy, and HIV positive. S1 Study protocol contains specific details regarding comorbidity and concomitant drug exclusions. All patients received single-dose tablets of 600 mg RIF, 300 mg INH, 1,500 mg PZA, 400 mg MFX, and 1,000 mg KAN dissolved in 5 ml normal saline (0.9% NaCl) before their scheduled surgery. Patients taking LZD and CFZ as part of their background regimens were considered at steady state if they had been on the drugs for more than 14 days. Patients receiving other drugs that were not the approved study drugs, such as LZD and CFZ, were not given these drugs on the day of surgery to minimize drug–drug interactions. These background drugs were still quantified and included in the data set analyzed and modeled in the present work, including appropriate dosing histories that had the exact times of their previous LZD and CFZ dose, which occurred more than 24 h before surgery. The lower limits of quantification (LOQ) were 1 ng/mL in plasma and 10 ng/g in tissue for both CFZ and LZD. Individual patient dosing times were planned to occur at approximately 2, 4, 8, 12, or 24 h before surgery with the aim of collecting longitudinal tissue sample concentrations. Plasma samples were drawn prior to and at the time of surgery. The PK sampling times served as proposed times, and no interference to the surgery occurred to obtain exact PK times. The drug administration was timed to occur before the surgery at various time points so different patients could contribute lesion samples at different time points post dose to reconstruct the entire PK profile. The exact resection times were recorded, and collected lung tissues were classified and dissected into defined lesions by macroscopic appearance and flash frozen for drug quantification. During resection, the pulmonary artery was clamped and resected tissue very briefly rinsed to avoid blood contamination in the tissue. Samples were further classified by histological and radiological methods into lung, necrotic nodule, caseum from closed nodule, caseous fibrotic nodule, caseum from cavity, cavity wall, fibrotic tissue, small nodule, and fungal ball (S1 Fig), quantified by HPLC-coupled tandem mass spectrometry (LC/MS-MS), and imaged by matrix-assisted laser desorption-ionization mass spectrometry imaging (MALDI-MSI). For large enough lesions that had discrete necrotic content, the lesion walls and caseum were analyzed separately.

### Plasma PK models

Plasma compartmental models were built for each drug based on established methodology. To capture the absorption, distribution, and clearance of the drugs, 1- and 2-compartment models with first-order elimination, parameterized in terms of oral clearance (CL), oral volume of distribution (V), peripheral volume of distribution (V2), and intercompartmental clearance accounting for drug movement between central and peripheral volume of distribution (Q), were tested and fitted to the data. To describe the process of absorptions, different established models were explored for the absorption profile of the drugs: a straight forward first-order model and, if necessary, a lag time and a transit compartment model with a fixed or estimated number of compartments were evaluated [11,12]. Within a population, we expect variability in some PK parameters, and to account for this distribution, interindividual variability (IIV) parameter was tested on plasma PK parameters and allowed based on the magnitude of estimates and the likelihood ratio test (LRT), which acts as a measure of statistical significance.

The plasma PK model for PZA, MXF, KAN, CFZ, and LZD was a 1-compartment model with first-order absorption described by the following equation:

$$\frac{dA}{dt}=k_{a}\times A_{1}-\left(\frac{CL}{V}\right)\times A_{2},$$ (1)

in which A_1_ is the amount of drug in the absorption compartment, A_2_ is the amount of drug in the central compartment in milligrams, CL is the clearance in liters per hour, and V is the volume of distribution in liters.

RIF PKs were described with a 1-compartment model linked to the transit compartment absorption chain, as described in Savic and colleagues [12], while the INH model was a 2-compartment model with first-order absorption.

### Tissue penetration model

Penetration of each drug into lung and lesions was described using a separate lesion compartment, as described by Kjellsson and colleagues [13], according to the following equations:

$$\frac{{dC}_{lung}}{dt}=k_{pl-lung}\times \left(R_{lung-pl}\times \frac{A_{plasma}}{V_{plasma}}-C_{lung}\right),$$ (2)

$$\frac{{dC}_{lesion}}{dt}=k_{pl-lesion}\times \left(R_{lesion-pl}\times \frac{A_{plasma}}{V_{plasma}}-C_{lesion}\right),$$ (3)

in which C_lung_ and C_lesion_ represent the drug concentration within uninvolved lung and lesion, KPL_−lung_ and KPL_−lesion_ are intercompartment rate constants for the transfer of drug from the plasma to lung or lesion, R_lung−pl_ and R_lesion−pl_ are the penetration coefficients (ratios) between lung or lesion and plasma, and A_plasma_/V_plasma_ is the drug concentration in plasma at time t. If the ratio value is 1, there is equal distribution between plasma and tissue; if the ratio is <1, there is reduced drug penetration from plasma to the tissue, and if the ratio is >1, there is accumulation of drug in the tissue compared to the plasma.

### Population PK model selection and evaluation

Concentration–time data for each drug in plasma, lesions, and uninvolved lung from each patient were modeled using a population PK methodology, which is the appropriate method for sparse longitudinal data with expected variability between patients and separation of the signal from noise due to random error. To best fit the sparse longitudinal data, we used nonlinear mixed-effects models with first-order conditional estimation methods as implemented in the software NONMEM (version 7.3; ICON Development Solutions, Ellicott City, MD, United States). Graphical, statistical, and exploratory analyses were conducted using the open-source software R (version 3.3.1). The Xpose (version 4.0) package, implemented within R, was used for graphical evaluations and visual predictive checks [14].

A stepwise approach was taken to model fitting. First, a structural PK model was fit to the plasma PK data, which contained the longitudinal samples in each patient, required to describe the population distribution. After establishing and validating the plasma PK model, PK data in uninvolved-lung and individual-lesion subtypes were added one lesion at a time, and the model was expanded in a stepwise manner, followed by assessment of parameters and goodness of fit. The plasma/lesion ratios and rates of drug uptake into the lesion compartments were estimated. It was assumed that the drug distribution into the lesion can only happen from the plasma, given general physiological knowledge of drug distribution. S2 Fig shows the influence of these KPL and RPL parameters on the PK profile from plasma to lesion, for which KPL influences the temporal delay as related to the plasma profile and RPL the magnitude change of the concentration in the tissue compared to plasma concentration. To account for accumulation of drugs into the lesions for patients at steady state, the lesion compartments were never reset after the dosing. To account for potential noise in the data, additive and proportional error models of residual variability were explored. Different residual errors were estimated for plasma, lung, and lesions. The LRT was used to evaluate statistical significance for inclusion of additional parameters in the nested models, with a decrease of 3.84 points (proportional to −2 log likelihood change), considered statistically different with 5% significance level assuming the LRT is *X*^*2*^ distributed. No additional variability was added to the lesion parameters, as the data were too sparse with a single-time point per patient. The variance seen for the lesion PKs follows expected plasma variance.

Finally, individual and population predictions of exposures at steady state were estimated in terms of the area under the concentration–time curve over 24 h at steady state (AUC_0–24_) and peak concentration (C_max_) in plasma, lung, and each lesion subtype. Simulations achieved 2 goals: (1) evaluation of the performance properties of selected models (visual predictive checks) and (2) exploration of PKPD relationships with alternative dosing scenarios.

### PK/PD exposure target selection

Antibiotic exposure to determine effective activity can be quantified using PK/PD indices: area under the concentration curve (AUC), maximum concentration (C_max_), and time, all relative to minimum inhibitory concentration (MIC). The final population PK parameters and interindividual variability were used to simulate PK profiles over the course of the treatment. Distributions of wild-type MIC were obtained from published epidemiological data based on clinical isolates tested according to standardized EUCAST/CLSI methodology as well as literature [15,16]. Target attainment analyses were performed using the upper limit of the published wild-type MIC distributions for each drug. Target analysis was not possible for PZA due to the drug’s apparent lack of antimycobacterial activity in vitro under standardized pH-acidic conditions. All simulations were performed using the mlxR package in R, with the final estimates and patient distribution from the established clinical PK model [17,18].

### Implementation of the model into interactive tool

A user interface to allow rapid simulations of the described lesion model and predict new data based on dosing regimen, microbiology values, and patient distribution was developed using the Shiny package from Rstudio [19,20]. The web application utilized the mlxR package for calculating and running the population model [17]. The code used is available within the web application and can be found at http://saviclab.org/tb-lesion/.

## Results

### Subject demographics and observed PK profiles

**Fig 1:**
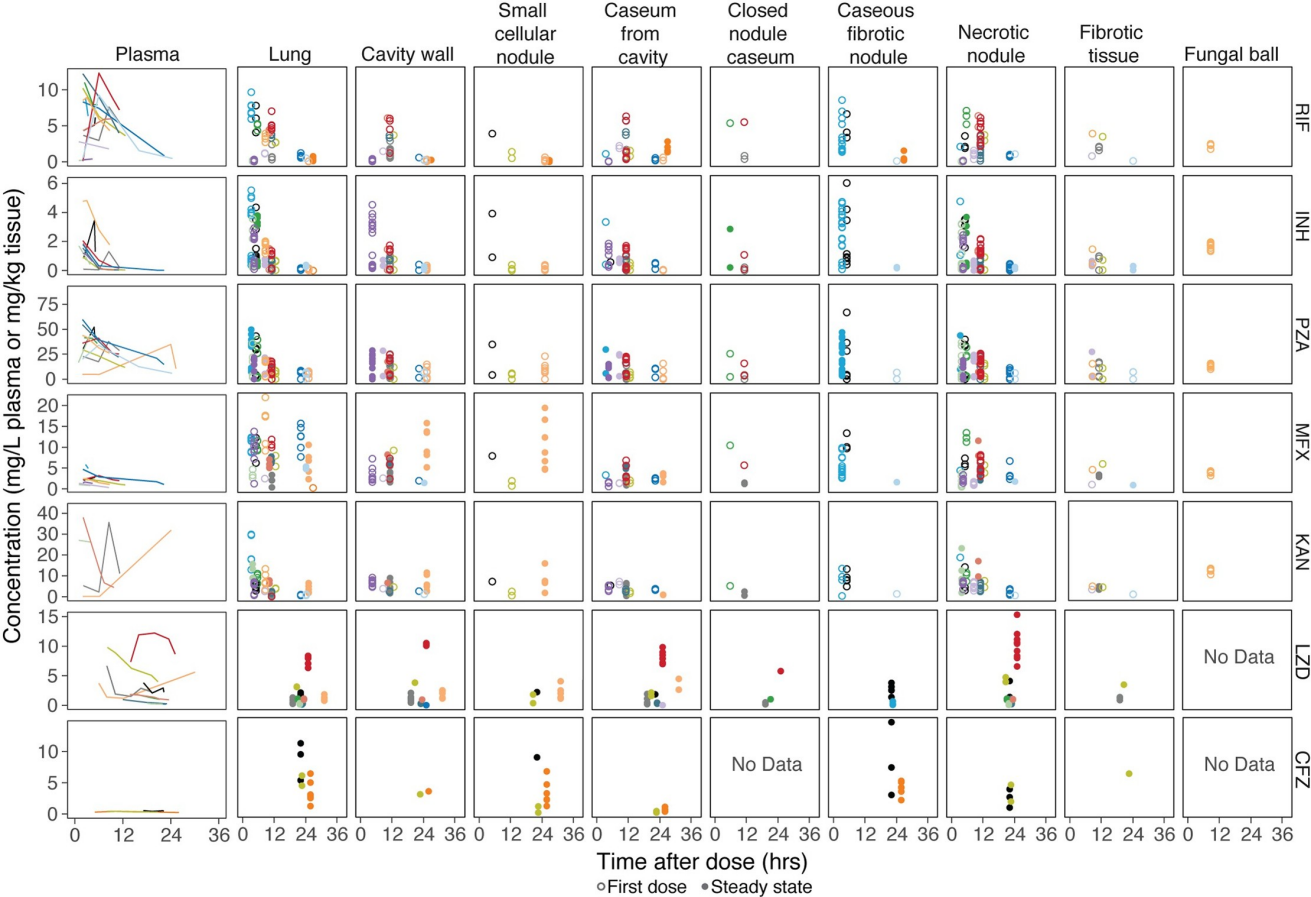
Raw clinical data for each drug in each lesion type. Concentration–time profiles are shown for 9 lesions and 7 drugs by respective panel. Plasma concentrations over time for each individual were measured at multiple time points after the time of drug administration and before lung resection and are shown as individual lines of different colors. Lesion concentrations were measured at a single time point (time of resection) per subject and are represented by circles of different colors that correspond to their individual subject plasma line. Closed circles show patients assumed to be at steady state, and open circles show patients receiving the drug for the first time. Some subjects received LZD and CFZ in their background regimen, and their last CFZ and LZD doses were administered on the day preceding the resection, leading to tissue samples after 24 h. None of the patients receiving LZD and CFZ had fungal ball lesions, and those patients on CFZ also didn’t have closed nodule caseum lesions. CFZ, clofazimine; INH, isoniazid; KAN, kanamycin; LZD, linezolid; MFX, moxifloxacin; NA, not available; PZA, pyrazinamide; RIF, rifampicin.

**Fig 2:**
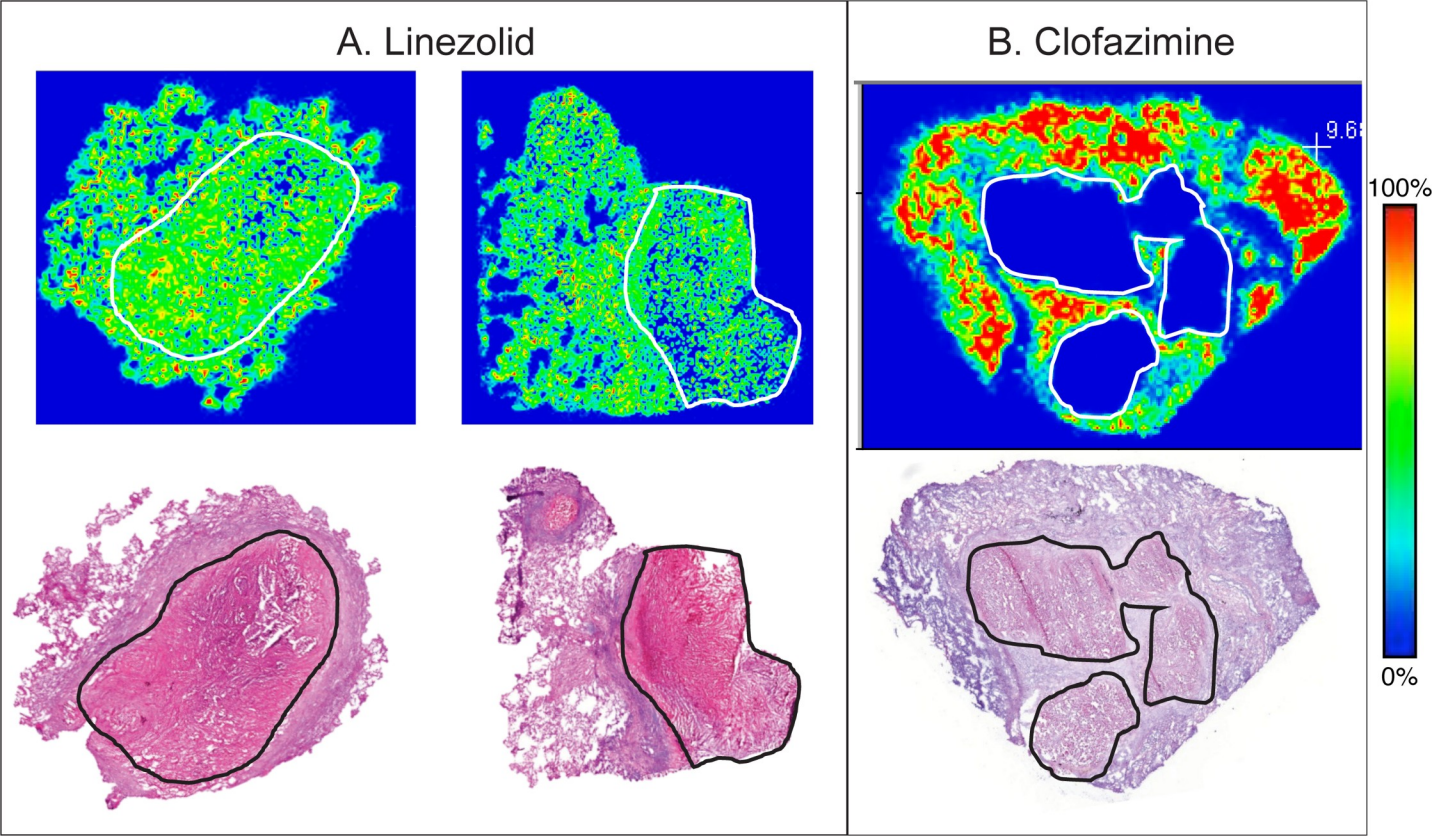
Two-dimensional imaging of LZD and CFZ by MALDI-MS. MALDI-MS ion maps of LZD and CFZ that provide a semiquantitative measure using the relative ion abundance of specific analytes in regions of interest. The highest signal intensity is fixed to 100% for each drug (red) and 0% indicating no detectable drug present (blue) on the rainbow color scale bar to the right. The caseum area is demarcated by the white line in the MALDI-MS image and corresponds to the black line of the histologically stained lesion images of the same sample in the bottom panel. A. The top panel of a nodule caseum and cavity lesion resected 20 h after LZD administration. LZD partitions favorably into cavity and nodule caseum in vivo, as indicated by the green color present in caseum and surrounding tissue showing similar concentrations in both. B. Clofazimine at steady state and 5 h post dose shows low penetration into the large necrotic granuloma in the top panel, as indicated by the blue area. CFZ, clofazimine; LZD, linezolid; MALDI-MS, matrix-assisted laser desorption-ionization mass spectrometry.

Previously described clinical study NCT00816426 collected lung lesion samples after elective lung resection from 10 male and 5 female patients aged 23 to 58 who failed TB treatment [7]. The demographics of the individual patients and their respective steady-state drugs are presented in Table 1. The PK data collected included samples from uninvolved lung, necrotic nodule, caseum from closed nodule, caseous fibrotic nodule, caseum from cavity, cavity wall, fibrotic tissue, small nodule, and fungal ball over a time range of 3–33 h, and plasma samples were taken prior to and at the time of surgery, with a total of 329 plasma and 1,362 lesion data points above LOQ included in the analysis. A summary of the number of observations for each drug, lesion, and number of patients contributing to each compartment can be found in S1 Table. The observed data of measured drugs in their respective lesions are shown in Fig 1. Drug concentrations differed greatly among lesions and patients and even within similar lesions of a single patient, demonstrating the high variance of TB drug distribution into lung lesions. Plasma and lesion concentrations of KAN and LZD are reported here for the first time (S1 Data) as well as spatial distribution of LZD and CFZ as measured by two-dimensional MALDI-MSI (Fig 2). From Fig 2, we observe that LZD seems to distribute equally between caseum and cellular tissue, suggesting high penetration into caseum. In contrast, CFZ shows little penetration into the caseum region and has a much higher affinity for cellular tissues surrounding the caseum area.

**Table 1:** Patient demographics.

Subject	Gender	Age (yrs)	BMI (kg/m^2^)	Prior TB episodes	Study steady-state drugs	Other steady-state drugs	NAT2 Polymorph (phenotype^§^)
1	Male	27	24.0	2	MFX, KAN	LZD, AUG, CLA	NAT2*4 (Fast)
2	Male	51	22.3	1	-	LFX	NAT2*4 (Fast)
3	Male	40	17.3	2	INH	EMB	NAT2*4 (Fast)
4	Female	53	22.0	8	-	-	^¥^NAT2*6A (Intermediate)
5	Male	54	29.4	2	-	LZD, AMK, PAS, CFZ	NAT2*4 (Fast)
6	Female	48	22.2	1	MFX	LZD, CS, AMX, PTH	NAT2*4 (Fast)
7	Male	43	27.1	2	MFX, KAN	LZD, PAS, AUG	NAT2*4 (Fast)
8	Male	59	19.2	1	-	-	NAT2*6 A (Slow)
9	Male	36	24.8	1	MFX, KAN, PZA	LZD, CS, PAS, PTH	NAT2*4 (Fast)
10	Female	23	18.8*	2	-	LZD, CS, CFZ	^¥^NAT2*7B (Intermediate)
11	Male	47	18.9	1	INH	LZD, CS, AUG, PTH, STM	NAT2*4 (Fast)
12	Male	39	22.2	4	PZA	CS, LFX, PTH, STM	NAT2*4 (Fast)
13	Female	58	21.2	1	KAN	LZD, CS, PAS	NAT2*4 (Fast)
14	Female	27	20.0	1	PZA	LZD, CS, AUG, STM	NAT2*4 (Fast)
15	Male	44	24.1	1	INH, PZA	EMB, LFX	NAT2*6A (Slow)

**Abbreviations:** AMK, amikacin; AUG, amoxicillin/clavulanate; CFZ, clofazimine; CLA, clarithromycin; CS, cycloserine; EMB, ethambutol; INH, isoniazid; KAN, kanamycin; LFX, levofloxacin; LZD, linezolid; MFX, moxifloxacin; NAT2, N-acetyltransferase 2; PAS, para-aminosalicylate; PTH, prothionamide; PZA, pyrazinamide; RIF, rifampicin; STM, streptomycin.

*Patient received LZD 450-mg dose due to low BMI.

^§^Acetylator phenotype inferred from NAT2 genotype.

^¥^Heterozygous allele.

### Plasma and tissue PK model

**Fig 3:**
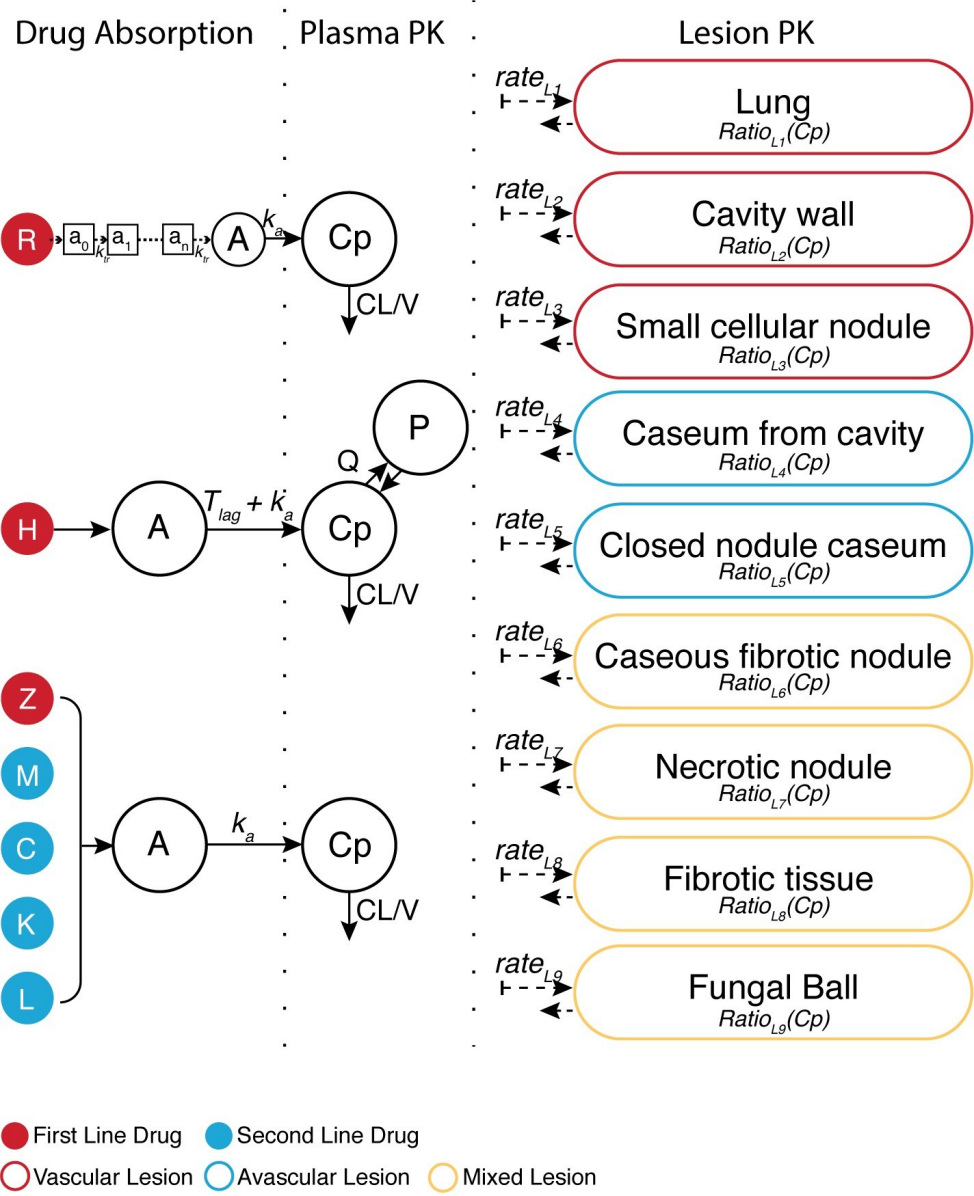
PK structural model. First-line drugs R, H, and PZA and second-line drugs M, C, K, and L were modeled individually with parameters CL, V, and k_a_ from an A describing Cps. R required a transit model to capture absorption, and H required an additional compartment (P) with Q and lag in time before absorption (Tlag) to model plasma data. Tissue concentration–time profiles were modeled with the addition of 2 parameters to describe the rate of drug absorption into the tissue compartment and the ratio of observed tissue concentration to plasma concentration. A, absorption compartment; C, clofazimine; CL, clearance; Cp, plasma concentration; H, isoniazid; K, kanamycin; k_a_, rate of absorption; L, linezolid; M, moxifloxacin; P, peripheral compartment; PK, pharmacokinetic; PZA, pyrazinamide; Q, intercompartmental clearance; R, rifampicin; V, volume.

**Fig 4:**
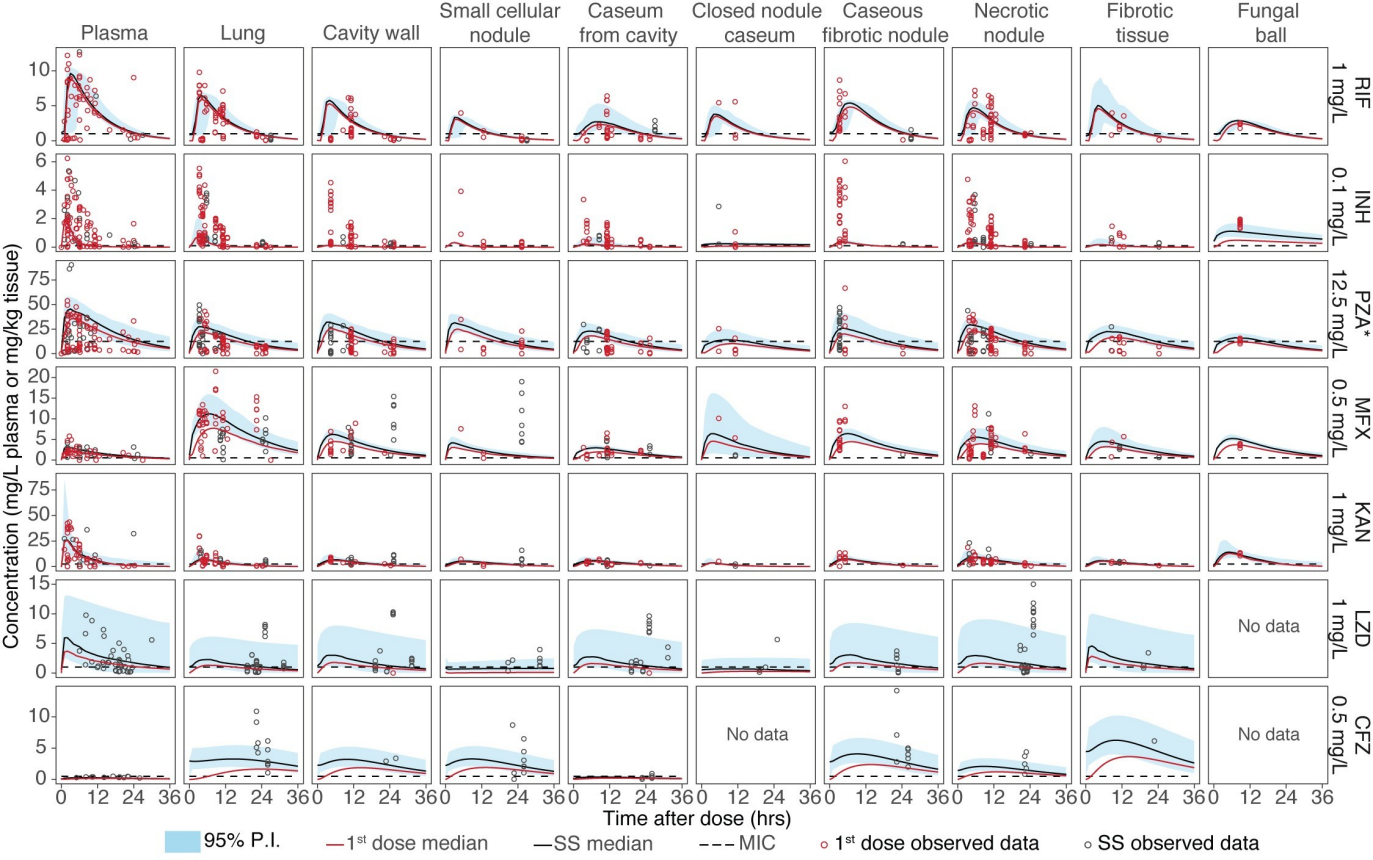
Visual predictive check of individual lesions and drugs. The visual predictive checks were simulated for 1,000 patients and are shown for all 7 drugs and 9 lesions. The simulated concentration–time profiles are represented by a solid black line to represent the median (typical patient) at steady state, with the shaded light blue area representing the 95% prediction interval of the 1,000 patients simulated. The red line represents the median of plasma–time concentrations after the first dose of each respective drug. The dashed black line represents the respective MIC of each drug and is displayed below the drug name. The original observed data is overlaid on top of the simulated bands as open circles to assess the ability of the model to capture both the median and distribution of the clinical data. Open red circles represent drug concentrations of patients receiving their first doses, and black open circles represent observed data for patients who were at steady state at the time of resection. First dose and steady state are overlaid in a single panel. A time-extended plasma visual predictive check is provided in S3 Fig. *PZA MIC of 12.5 mg/L is shown and is specific to an acidic environment less than pH 5.8. MIC, minimum inhibitory concentration; CFZ, clofazimine; INH, isoniazid; KAN, kanamycin; LZD, linezolid; MFX, moxifloxacin; NA, not available; PZA, pyrazinamide; RIF, rifampicin.

The structural model describing the plasma PKs and distribution of RIF, INH, PZA, MFX, KAN, LZD, and CFZ in the 9 lung tissue compartments is summarized in Fig 3. A 1-compartment model with first-order absorption best described the plasma data of PZA, MFX, KAN, LZD, and CFZ. RIF model–building required a transit absorption compartment to capture the observed absorption data, and INH PKs best fit a 2-compartment model with first-order absorption and lag time. INH undergoes N-acetyltransferase 2 (NAT2) mediated conversion to acetyl-INH in the liver. Due to widespread genetic polymorphisms in NAT2 and a demonstrated correlation between polymorphism and INH concentration–time profile [21], INH profiles were characterized for slow, intermediate, and fast acetylators with 11 out of the 15 subjects phenotyped as fast acetylators, i.e., high metabolizers. Further discussion, therefore, focuses on fast acetylators and their increased risk of treatment failure. Established models were evaluated by a visual predictive check of the final model, stratified by drug and lesion, shown in simplified Fig 4 indicating appropriate model fit with overlapping of first dose and steady state. A more complete plasma concentration visual predictive check is provided in S3 Fig. The final estimates of the parameter values from the plasma PK models are shown in Table 2. Clearance values for all drugs except INH were within reported literature ranges, as shown in the S2 Table, showing that this subpopulation of patients exhibits similar PK profiles to the general population. Tissue distribution was best described by separate compartment with 2 distinct parameters: parameter to describe the rate of drug movement from plasma to each lesion type or compartment (KPL) and a parameter to estimate the ratio of drug concentration observed in the tissue compared to the concentration in plasma (RPL). Table 3 shows the final estimated penetration ratios of each drug in the 9 types of lung and lesion compartments. The plasma-to-tissue distribution rate constants are detailed in S3 Table.

**Table 2:** Plasma PK parameters.

Parameter	RIF	INH	PZA	MFX	CFZ	KAN	LZD
Rate of absorption (h^−1^)	1.55 (0.0434)	0.738 (0.150)	0.554 (0.183)	1.55 (1.50)	0.100 (0.349)	1.65 (0.066)	2.13 (0.010)
Lag time (h)	-	0.130 (0.230)	-	-	-	-	-
Clearance (L/h)	5.72 (1.44)	s: 9.41 (3.26) i: 24.0 (9.41) f: 38.1 (5.41)	2.05 (0.312)	8.93 (1.50)	16.3 (0.109)	4.61 (0.251)	3.77 (0.082)
Central volume (L)	52.3 (12.5)	48.9 (9.98)	30.0 (2.71)	147 (32.9)	280 (0.273)	30.6 (0.167)	145 (0.530)
Intercomp clearance	-	6.43 (1.86)	-	-	-	-	-
Peripheral volume (L)	-	40.4 (14.3)	-	-	-	-	-

**Abbrievations:** CFZ, clofazimine; f, fast; i, intermediate; INH, isoniazid; KAN, kanamycin; LZD, linezolid; MFX, moxifloxacin; NONMEM, NONlinear Mixed Effects Modeling; PK, pharmacokinetic; PZA, pyrazinamide; RIF, rifampicin; s, slow.

*Standard error shown in parentheses describes the precision of the parameters generated by NONMEM. INH clearance was estimated for 3 subgroups representing s, i, and f metabolizers.

**Table 3:** Estimated drug lesion ratios.

Drug	Lung	Cavity wall	Small cellular nodule	Caseum from cavity	Closed nodule caseum	Caseous fibrotic nodule	Necrotic nodule	Fibrotic tissue	Fungal ball
RIF	0.694 (0.19)	0.614 (0.16)	0.348 (0.18)	0.449 (0.32)	0.443 (0.25)	0.733 (0.29)	0.569 (0.12)	0.539 (0.18)	0.447 (0.07)
INH	0.552 (1.20)	0.175 (0.80)	0.228	0.298 (0.42)	0. 824 (0.04)	0.516 (0.04)	0.376 (0.52)	0.267 (2.79)	3.04 (0.0004)
PZA	0.616 (0.17)	0.72 (0.02)	0.698 (0.04)	0.544 (0.13)	0.394 (0.32)	0.611 (0.032)	0.676 (0.09)	0.607 (0.04)	0.429 (0.001)
MFX	4.41 (2.29)	2.33 (0.77)	1.34 (1.46)	1.26 (0.37)	1.83 (2.24)	2.41 (0.99)	2.29 (1.16)	1.69 (1.46)	2.08 (0.01)
CFZ	12.4 (0.10)	11.3 (3.68)	11.47 (2.55)	1.34 (0.48)	NA	14.3 (0.30)	7.32 (1.38)	22.03 (4.28)	NA
KAN	0.404 (0.61)	0.468 (0.96)	0.422 (1.12)	0.468 (0.13)	0.176 (0.17)	0.503	0.667 (0.46)	0.404 (1.12)	0.918
LZD	0.609 (0.10)	0.497 (0.34)	0.531 (0.24)	0.651 (0.036)	0.673 (0.016)	0.177 (0.073)	0.609 (0.040)	0.487 (0.114)	NA

**Abbrievations:** CFZ, clofazimine; INH, isoniazid; KAN, kanamycin; LZD, linezolid; MFX, moxifloxacin; NA, not available; PZA, pyrazinamide; RIF, rifampicin.

*Standard error shown in parentheses describes the precision of the parameters.

#### Rifampin

Overall, RIF distributed reasonably well into all lesion subtypes and compartments, with penetration ratios ranging from 0.3 to 0.7. RIF concentrations were below the MIC for less than 50% of the dosing interval in all tissue compartments in typical patients. Only one patient had received multiple RIF doses, limiting the possibility to establish steady-state residence time in lesions of interest.

#### Isoniazid

Concentrations of the other first-line drug, INH, were below the MIC for more than 10 h of the dosing interval in the majority of lesion subtypes, except for fungal ball tissue. Comparing tissue-penetration coefficients of INH between lesions, higher penetration was observed in caseum from closed nodules and caseous fibrotic nodules, suggesting more rapid and favorable penetration and affinity in this more difficult-to-treat caseous environment. However, it has been found that INH has no detectable activity against nonreplicating bacilli found in caseum [22], indicating that despite favorable penetration, adequate concentrations are likely not reached in this compartment.

#### Pyrazinamide

PZA is considered a treatment-shortening and sterilizing drug and performs best in the acidic environments thought to be present in the phagolysomes of infected macrophages and in the necrotic foci of closed nodules and cavities [23]. Differentiating lesion subtypes based on their likely pH microenvironments, PZA should be most active in necrotic nodules, macrophage rich lung, cavity wall, cavity caseum, closed nodule caseum, and small cellular nodules. Considering lowered MIC in these lesion compartments, PZA concentrations were above the “acidic” MIC for 9 h of the dosing interval in the caseum of closed nodules and for at least 70% of the dosing interval in lung tissue, cavity wall, cavity caseum, cellular and necrotic nodules, and fibrotic tissue.

#### Moxifloxacin

Second-line drugs MFX and CFZ (100 mg daily) had the highest lesion-penetrating ratios with higher observed tissue than plasma concentrations. As a result, MFX was above the MIC for the dosing interval in all lesion subtypes, including caseous lesions.

#### Clofazimine

CFZ had high-penetration ratios into cellular tissues, such as uninvolved lung, cavity wall and small cellular nodules, and fibrotic lesions, translating into concentrations above its MIC for the entire dosing interval. Penetration in tissues with little or no cellularity, such as caseum from cavity, was low relative to other lesions. Thus, CFZ is expected to reach cellular—but not caseous—lesion compartments at adequate concentrations, in line with its reduced efficacy in mouse models that present large necrotic lesions [24]. In addition, like INH, CFZ has no detectable activity against nonreplicating bacilli found in caseum [22].

#### Kanamycin

A 1,000-mg dose of KAN had lower concentrations in tissues compared to plasma and had a very low tissue-to-plasma ratio of 0.176 in caseum from closed nodules. The highest estimated ratio of KAN was in necrotic nodules corresponding to the high C_max_/MIC and a long time above MIC profile.

#### Linezolid

The typical profile of 300 mg LZD had best concentration levels and tissue-to-plasma ratios in necrotic foci. Within most of the caseous lesions, LZD concentrations were well maintained above MIC after reaching steady state.

### At-risk patients show low target attainment in lesions

**Fig 5:**
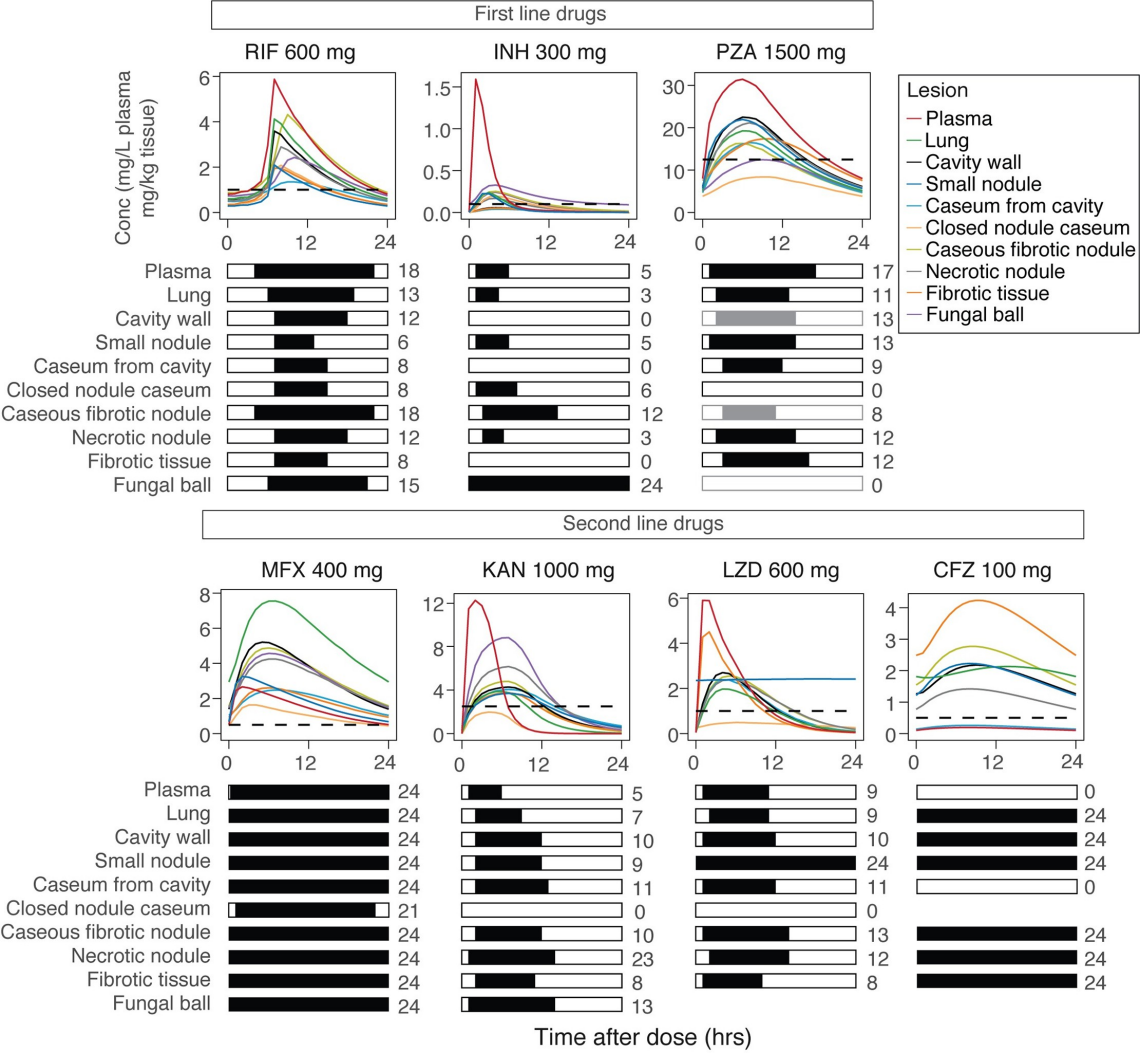
PK profiles of individual drugs in at-risk patients relative to MIC. One thousand patients were simulated using the established model and the fifth percentile of lower exposure shown with facets for individual drugs to compare drug penetration into respective lesions shown by different colors. The dashed line in the concentration–time panel represents each drug’s respective MIC, and the bar graph below simplifies drug concentration above or below by black and white bars for each lesion. Additionally, the total time drug concentration is above MIC at steady state over 24 h is printed next to each respective bar. Importantly, PZA’s MIC of 12.5 mg/L is low-pH specific, which does not represent all the lesions’ pH environment. For the lesions with likely higher pH environments and assumed less PZA activity, gray bars instead of black bars are used to indicate drug concentration above MIC. MIC, minimum inhibitory concentration; PK, pharmacokinetic; CFZ, clofazimine; INH, isoniazid; KAN, kanamycin; LZD, linezolid; MFX, moxifloxacin; NA, not available; PZA, pyrazinamide; RIF, rifampicin.

Next, the population PK model was implemented as a simulation tool to stratify patients at risk of treatment failure. The population PK parameter estimates in our population corresponded well to reported literature values (S2 Table) in general TB patient population; therefore, we assume that this population is similar in its PK profiles to the general population. Considering that 95% of TB patients are successfully cured at the completion of standard treatment for drug-susceptible TB, we investigated the lower fifth percentile of patient concentration–time profiles in plasma to account for the fraction of the population group that fails treatment. This fifth percentile, was simulated from 1,000 repetitions at steady state for each drug in lesion subtypes. The time above MIC is visualized for an at-risk patient in Fig 5. The simulated at-risk population showed that first-line drug concentrations do not stay above the respective MIC for the entire dosing interval. The horizontal timeline bars illustrate the period during which a drug is above or below its MIC within a dosing interval in each of the 9 tissue or lesion types. On average, RIF was below MIC values for hard-to-treat necrotic foci for less than 50% of the dosing interval in at-risk patients. While INH reached adequate concentrations in fungal ball tissue for the entire dosing interval and in caseous fibrotic nodule for half of the dosing interval, in other lesions, INH was below its MIC more than 75% of its dosing interval. In at-risk patients, PZA achieved concentrations above the MIC for 38%–55% of the dosing interval in almost all lesion types, with caseum from cavity, closed nodule caseum, and caseous fibrotic nodule showing the longest below-MIC periods, assuming an optimal acidic MIC of 12.5 mg/L in all lesions.

Second-line CFZ achieved adequate exposure in cellular lesion compartments, consistent with its higher intracellular uptake in macrophages and other immune cells. MFX performed better than most drugs in all lesions, and while LZD performed well in all lesions in typical patients, at-risk patients had less than 50% above-MIC concentrations—except for small cellular nodules. The ratio between peak concentration (C_max_) and MIC is generally considered the PK/PD driver of aminoglycosides. In at-risk patient populations, the C_max_/MIC of KAN was approximately 2-fold lower than in patients with standard exposure. Across drugs, closed nodule caseum was the most problematic lesion compartment.

### TB drugs do not reach their PK/PD target in all lesions

For most TB drugs, the exposure/potency index that drives efficacy is the ratio of the area under the concentration curve to the MIC (AUC_0–24_/MIC). Published AUC_0–24_/MIC ratios—associated with 90% maximal effective concentrations (EC_90_)—used in the present simulations were 1,360 for RIF, 567 for INH, 56 for MFX, and 25 for LZD [15,16,25,26]. Aminoglycosides, like KAN, generally show C_max_-dependent killing, and a C_max_/MIC value greater than 10 is thought to result in 90% killing in the treatment of pneumonia [27]. In the absence of experimental and clinical data for CFZ, 90% time above MIC was chosen as the PK/PD index.

**Fig 6:**
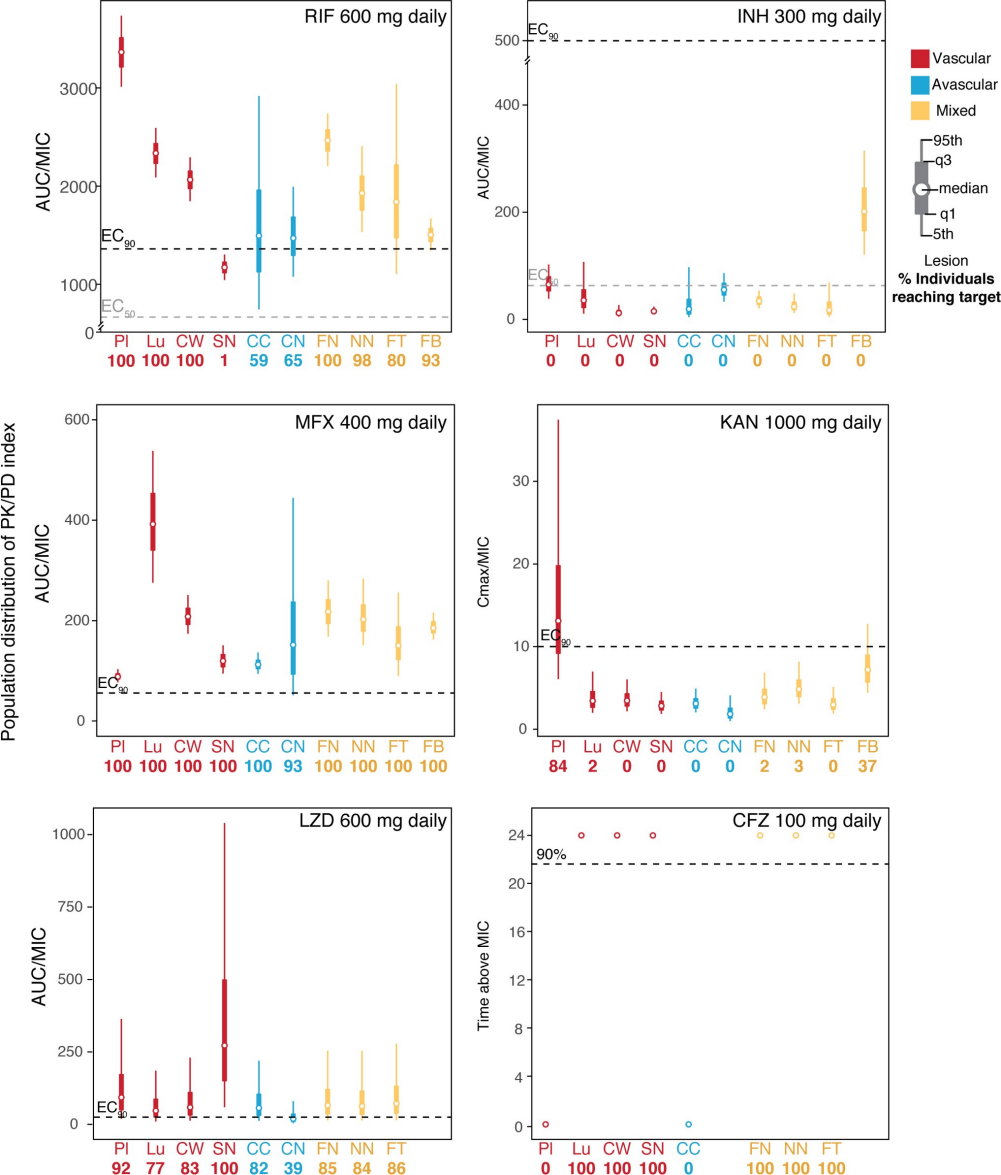
Distribution of PK/PD target attainment per drug. Exposure of 1,000 simulated patients are represented as box plots in each lesion to visualize the distributed PK/PD efficacy for individual drugs and lesions. Each panel represents an individual drug given at their standard dose until steady state is reached. The dashed lines indicate published target attainment PK/PD ratios, for which black dashed lines indicate the critical exposure values able to achieve ≥90% of maximal kill (EC_90_) values and gray dashed lines for drugs with published critical exposure values able to achieve ≥50% maximal kill (EC_50_). The percentage of the 1,000 simulated patients are printed below each box plot. Lesions are colored by their vascularity. CC, caseum from cavity; CN, closed nodule caseum; CW, cavity wall; EC_90_, 90% maximal effective concentrations; EC_50_, 50% maximal effective concentrations; FB, fungal ball; FN, caseous fibrotic nodule; FT, fibrotic tissue; Lu, lung; NN, necrotic nodule; PK/PD, pharmacokinetic/pharmacodynamic; Pl, plasma; SN, small cellular nodule.

Using simulations that represent the entire population, the results of the patient target attainment simulation in lesions are shown in Fig 6. The simulation consisted of 1,000 patients and showed improved outcomes for MFX and RIF, but INH and KAN performed poorly across lesions, with only a small fraction of the patient population reaching target breakpoints of efficacy. CFZ performed well across all lesion types except caseum from cavity.

### Simulations can guide the selection of the best multidrug options for patients with cavitary lesions

**Fig 7:**
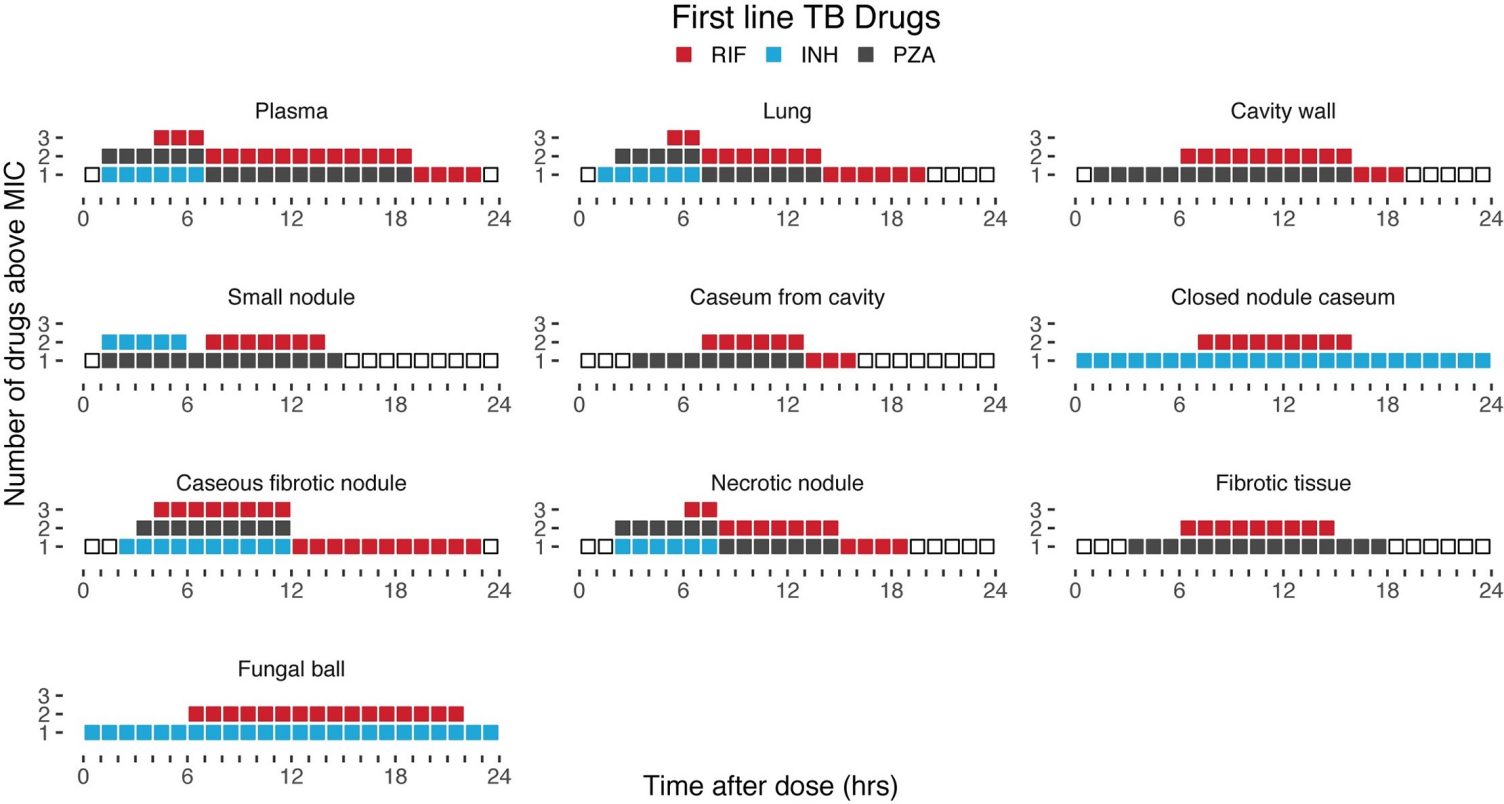
Number of drugs above MIC by lesion type. Number of drugs above MIC over time plot of first-line drugs at steady state, collected as the fifth percentile (low-exposure or at-risk patients) of 1,000 simulated patients over the 24-h dosing interval. Each square represents 1 h that a drug is above the MIC. The drugs are stacked for each hour with different colors representing different drugs. Empty squares show no drug on board. MIC, minimum inhibitory concentration.

Overall, the plasma and lesion PK data collected in this study clearly highlight the impact of interindividual PK variability and differential lesion penetration on PK/PD target attainment. Importantly, the simulations uncover the potential for actual monotherapy in specific lesion compartments where several drugs in the regimen do not reach adequate concentrations, creating windows of opportunity for the development of resistance in patients with low exposure (Fig 7). The first-line regimen (RIF-INH-PZA) performed poorly in cavity and closed nodule caseum, with only a maximum of 2 drugs with suitable drug levels in each lesion and sufficient windows of monotherapy that could lead to resistance. Second-line drugs are included in S4 Fig. Collectively, the second-line drugs exhibited limited time above MIC in cavity caseum.

**Fig 8:**
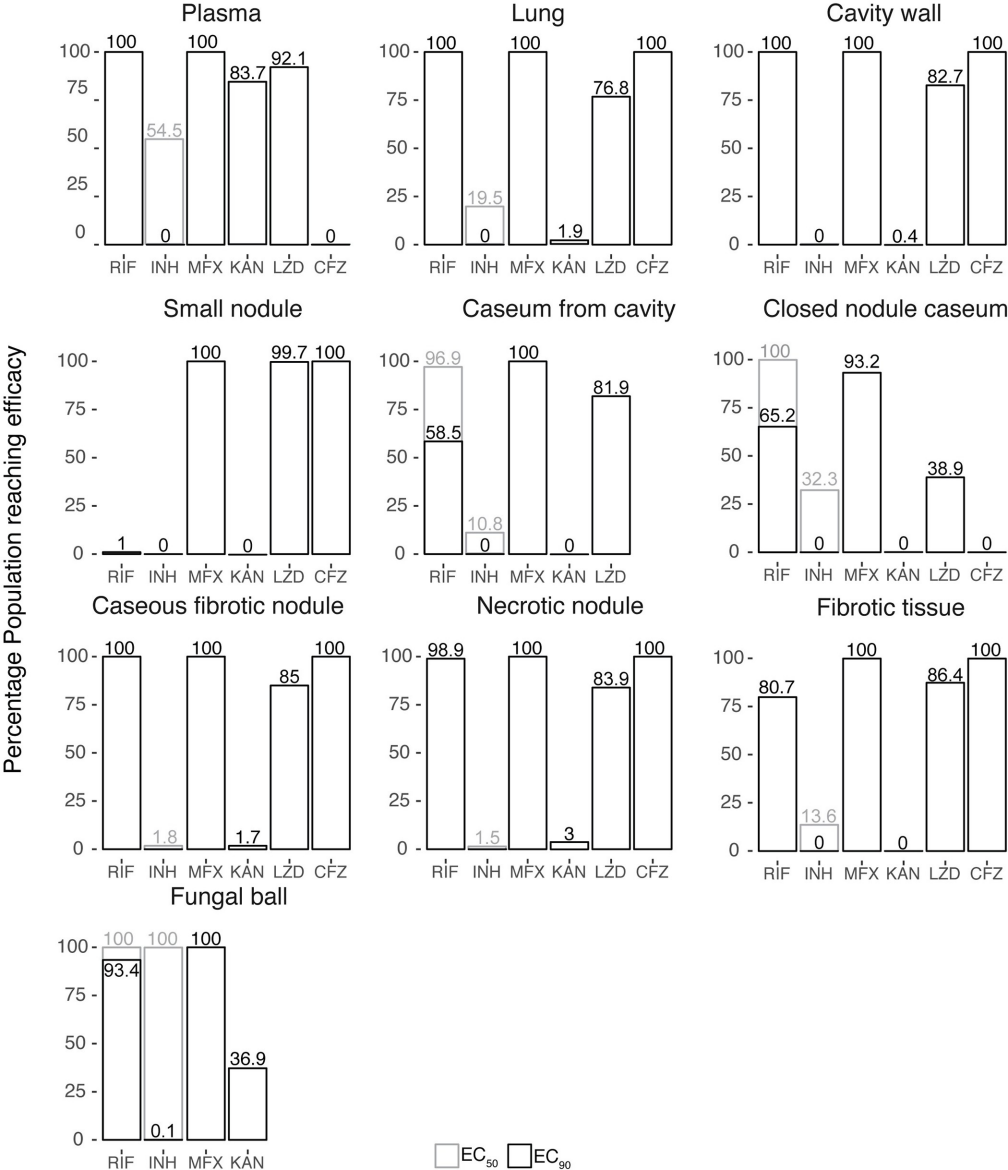
Prediction of drug efficacy by lesion type. Using defined critical PK/PD exposure values, a population of 1,000 simulated patients were defined as reaching efficacy or not from their PK simulations. The percentage of those reaching efficacy are shown as percentage box plots to compare the drugs that are able to reach a higher ratio of efficacy in specific lesions. Black boxes represent the percentage population reaching EC_90,_ and if available, gray boxes represent the percentage population able to reach EC_50_. EC_90_, 90% maximal effective concentrations; EC_50_, 50% maximal effective concentrations; PK/PD, pharmacokinetic/pharmacodynamics.

Using the PK/PD indices described in the previous section, we calculated the percentage target attainment of each drug in each lesion type to identify regimens that would be effective in specific lesions (Fig 8). Worst outcomes were predicted in cavity caseum and closed nodules, with only RIF, MFX, and LZD reaching effective concentrations. First-line drug RIF outperformed INH, which seldom impacted the population percentage reaching PK/PD targets. CFZ exhibited a contrasted profile, achieving high population percentages of target attainment in cellular lesions while not contributing to efficacy in caseum. KAN did not meet expectations and could benefit from dose optimization to ensure that lesion concentrations are adequate to inhibit bacteria; however, KAN’s severe safety profile would limit such optimization.

**Fig 9:**
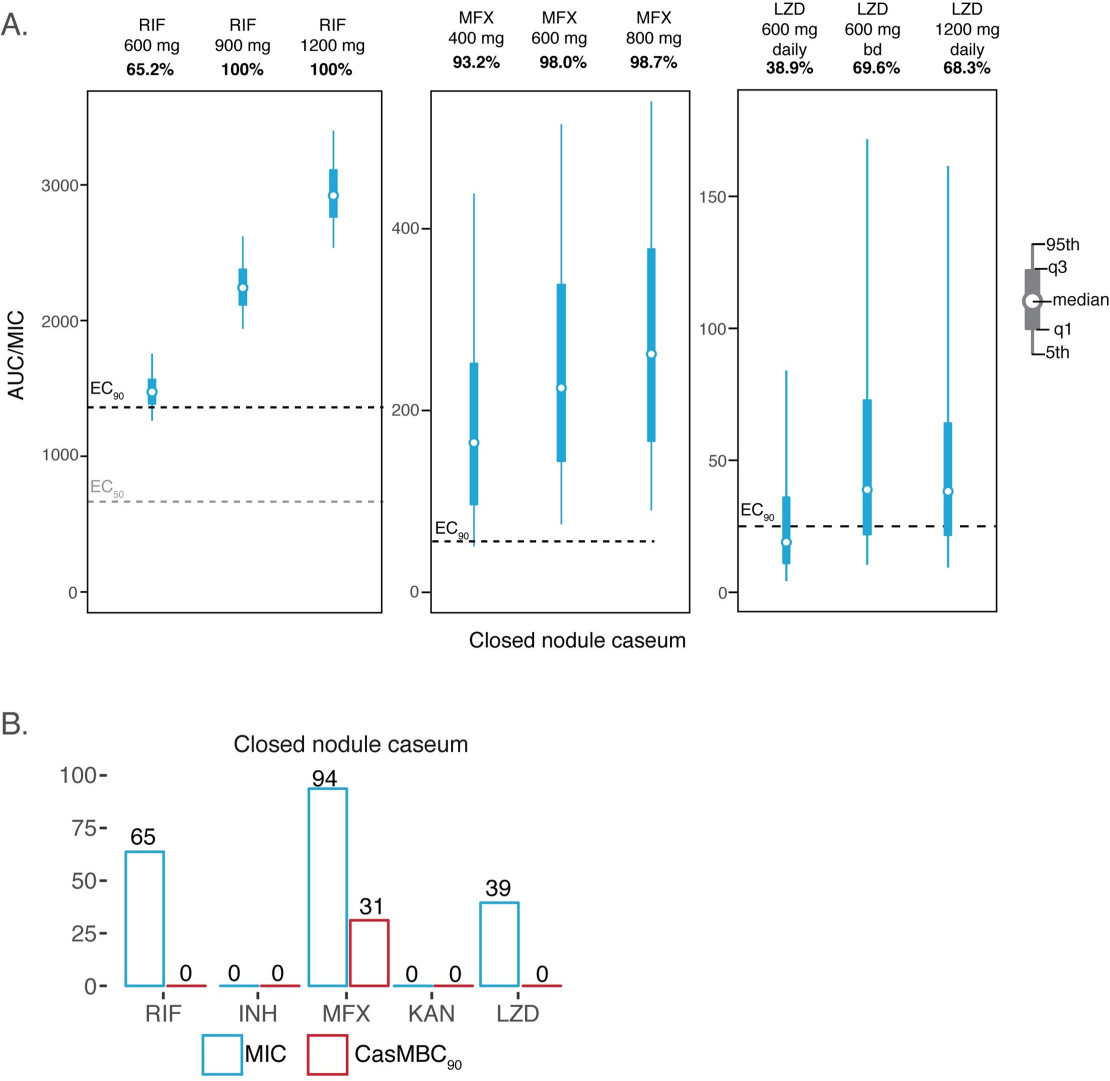
Predicted contribution of drugs by lesion type. A. RIF, MFX, and LZD simulations were performed at higher doses to observe their impact in hard-to-treat caseum from closed nodule. Their respective exposure distribution in 1,000 patients are shown in relation to their critical exposure value to reach EC_90_ (black lines), and EC_50_ (gray bars) if available. B. The percentage PK/PD efficacy target reached by a 1,000 simulated patient PK profiles as defined by their drug exposure versus drug MIC is shown in blue. Red boxes use CasMBC_90_ instead of MIC in the same set of simulated patients to compare that efficacy is both lesion and drug specific. EC_90_, 90% maximal effective concentrations; LZD, linezolid; CasMBC_90_, caseum-specific minimum bactericidal concentration; MIC, minimum inhibitory concentration; MFX, moxifloxacin; PK/PD, pharmacokinetic/pharmacodynamic; RIF, rifampicin.

To further examine if the drugs could perform better at increased doses, dose-escalation simulations of 600 mg and 800 mg MFX were performed but did not produce significant changes in outcome (Fig 9A). High-RIF doses of 900 mg or 1,200 mg predicted 100% target attainment in the simulated population. Simulated LZD doses of 600 mg twice daily and 1,200 mg daily showed improved efficacy outcomes almost 2-fold. Considering LZD toxicity is concentration driven, a 600-mg twice-daily dose would decrease risk of toxicity while still maintaining AUC values similar to a 1,200-mg daily dose [28]. To account for the phenotypic drug tolerance of *Mycobacterium tuberculosis* (Mtb) bacilli in nodule and cavity caseum [29], additional population PK/PD distributions were simulated using caseum-specific minimum bactericidal concentration MBC_90_ (CasMBC_90_) as the PD parameter to capture the drugs’ killing potential in the caseous core of necrotic lesions (Fig 9B). None of the drugs were able to reach full efficacy in this hard-to-treat lesion, and MFX was the only drug to show efficacy for 31% of the simulated population. Additional results using MBC_90_ and casMBC_90_ in other lesions are shown in S5 Fig.

### Implementation of the established clinical model into interactive research and clinical in silico tool

**Fig 10:**
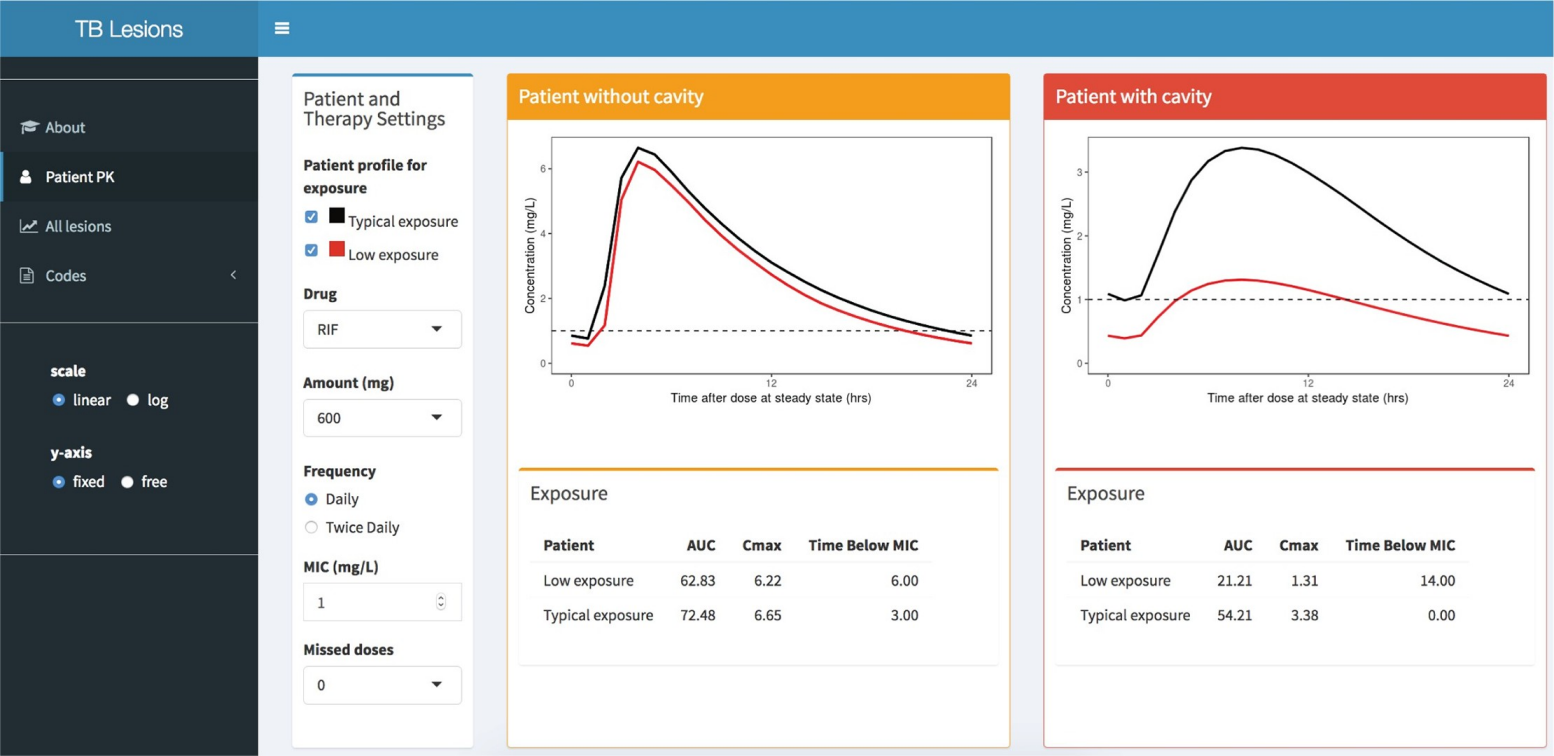
Interactive web application. A page of the web app is shown that displays 2 scenarios of a patient presenting with or without cavity. The user can investigate the patient’s exposure distribution, dosing, and microbiology properties.

An online tool for the established model was developed to simulate different doses and schedules for each of the 7 drugs in each specific lesion for any user-entered MIC. This interactive tool can generate critical knowledge essential for drug treatment and regimen optimization in a clinical trial setting by highlighting those patients at lower exposure that are at risk of treatment failure and stratifying patients with and without cavity. The web app is hosted at http://saviclab.org/tb-lesion/, with snapshots of 1 panel shown in Fig 10.

## Discussion

We have described and modeled the penetration of 7 first- and second-line TB drugs in 9 different lesion types from clinically resected lung samples. The population PK and tissue distribution model successfully captured the data and observed variance.

Overall, MFX, LZD, and RIF achieved the best coverage across lesion types relative to published MIC values. Our MFX results are aligned with a recent study by Kempker and colleagues, who measured free-cavitary MFX concentrations between 1 and 6 μg/mL approximately 2 h after drug administration [30]. In our simulations, to explore caseum-specific MBC (8 μg/mL for RIF and 2 μg/mL for MFX), RIF and MFX were remarkably the only 2 study drugs that could reach the concentrations required to kill 90% of nonreplicating Mtb bacilli in caseum [22]. However, despite its good cidal activity in caseum, MFX does leave a substantial population of persisters behind [22]; this could explain its lack of sterilizing activity. Considering the disappointing results of the REMoxTB and RIFAQUIN trials, in which MFX failed to shorten TB chemotherapy when substituted for either INH or ethambutol in the first-line treatment of DS-TB [31,32], more studies into lesion-focused penetration and activity could guide better decision-making for regimen and trial designs. In addition to MFX’s poor sterilizing activity, Drusano and colleagues demonstrated an antagonism between RIF and MFX against nonreplicating bacilli in the hollow-fiber system [33]. Additionally, there is a significant and clinically relevant drug–drug interaction between RIF and MFX, reducing MFX levels up to 40%, which would require dose adjustment, but is often not implemented due to QT prolongation concerns [34]. In light of these observations, our results suggest that the clinical utility of MFX may be maximized in a regimen that does not include rifamycin.

None of the drugs evaluated could reach their respective PK/PD targets in 100% of patients with closed caseum nodules. The best outcome was seen with MFX at 93.2% and RIF at 65.2%. CFZ reached adequate exposure in all cellular lesion types and lesion compartments, for which PK/PD modeling indicates it contributes to regimen efficacy in a major way, except for cavity caseum, for which CFZ failed to meet target attainment. This suggests that CFZ should always be paired with TB drugs that can reach and kill bacterial populations residing in necrotic lesion compartments. This could include RIF, MFX, or LZD, but care should be taken to ensure that patients are not resistant to RIF if CFZ is used as a second-line drug. Considering these outcomes, RIF-MFX-CFZ with or without LZD could potentially be the best treatment regimen to treat the majority of patients with diverse lesions. A stratified treatment approach based on the lesion profiles of patients could also be useful, for which patients with noncavitary TB and limited pathology could be adequately treated with a short course regimen containing CFZ. Our study offers further rationale for use of stratified medicine approaches in TB care for which the right treatment as well as the right dose could be matched with the right patient to ensure optimal treatment for all patients.

INH showed poor overall distribution and did not reach any PK/PD targets. In our cohort, the majority of the patients were fast acetylators (11 out of 15), resulting in very low plasma levels in a majority of the patients, which could skew the lesion results toward very low levels. Considering these results, and based on our simulations, INH may not perform so poorly in other regions of the world where distribution of acetylator status is more balanced. Administration of high-INH dose could also help; for example, based on our simulations, administration of a daily 15-mg/kg dose in slow acetylators would result with some coverage in caseum from cavity and closed nodule caseum.

KAN was another drug that performed poorly, and considering aminoglycoside cross-resistance, serious side effects, and a less favorable view of injectable drugs for TB treatment due to adherence problems, the use of KAN proves to be limited and of little value, as has recently been confirmed in new WHO Rapid Communication: Key changes to treatment of multidrug- and rifampicin-resistant tuberculosis (MDR/RR-TB) Guideline [35,36].

LZD showed good penetration into all lesions, confirming that LZD remains an excellent drug choice for TB treatment, as recently observed in clinical trials or extensively drug-resistant TB (XDR-TB) from an efficacy standpoint [37]. Use of LZD will remain limited by its toxicities, namely anemia and neuropathy, and an optimal risk/benefit ratio is yet to be determined.

For all of our simulations, we have used general MIC targets as recommended by standardized European Committee on Antimicrobial Susceptibility Testing/Clinical & Laboratory Standards Institute (EUCAST/CLSI) methodology as well as literature [15,16]. The accuracy in MIC measurements and any uncertainties around these values would carry over to the simulations. More precise assessment of MIC, as recently recommended by Alland and colleagues, would benefit accuracy and precision of our simulations [38]. For this reason, our interactive tool allows the MIC of drugs to be adjusted by the user as new mycobacterial targets become available.

Our patient population failed their initial TB treatment, and one of the potential reasons could be overall low drug levels leading to suboptimal therapy. Low drug exposures could be a consequence of nonadherence, reduced bioavailability, or increased clearance, all leading to suboptimal drug levels compared to the general population. However, the plasma PK parameter estimates from our models were within range of reported literature values. A recent patient-pooled analysis showed that HIV seropositivity, low BMI, adherence, baseline burden, and cavitation were all significant risk factors for treatment failure [39]. Both cavitation and adherence would lead to reduced levels in the cavities despite satisfactory plasma levels. Indeed, as shown in our study, drug levels in the cavities were very much reduced, which can explain why cavitation is an important risk factor for poor treatment response and might contribute to failing treatment in our cohort. Indeed, all patients had TB lesions so significant and diverse that they required lung resection as treatment. Our study offers a pharmacological and mechanistic explanation as to why cavitation indeed is such an important risk factor for TB outcome.

Most of the TB drugs show large between-patient variability in plasma drug levels. To explore the impact of PK variability, we have conducted population simulations to explore how patients with very low plasma levels would perform in terms of lesion PK and PK/PD target attainment, providing further rationale for the likelihood of poor clinical response due to suboptimal PK. First- and second-line drugs showed suboptimal concentrations in the lesions of these patients, especially cavity caseum and closed nodule caseum, in agreement with the poor prognostics associated with cavitary TB [39,40]. This also further suggests that a majority of the TB drugs might not be used at their optimal dose.

Emergence of resistance is prevalent in cavities, and different resistant isolates have been retrieved from different cavities of the same patient [41,42], pointing toward diverging resistance evolution, depending on lesion-specific context (bacterial load, drug tolerance of local populations [22], and fluctuating drug levels). Such lesion-specific acquired resistance is consistent with suboptimal penetration of a subset of the TB drugs in nonvascularized cavity caseum [7,9,43,44]. Our results, together with the recently demonstrated correlation between sub-breakpoint MIC and relapse [38], point in the direction of spatial and temporal windows of de facto monotherapy for which only one drug is present at adequate (i.e., above the concentration required to inhibit and/or kill local bacterial populations) concentrations during a significant portion or all of the dosing interval.

The limitations of this study include the relatively small sample size and the fact that individual tissue samples could only be taken at one time point for each patient, which is the time of the resection surgery. An additional limitation is that only one subject was at steady state for RIF. While a nonlinear mixed effects approach is useful to evaluate sparse data sets, RIF estimates did not capture the high caseum retention times observed in this specific patient [45], indicating that steady-state exposure of RIF could have been underestimated in the current model; however, any such interpretation is limited given this observation in only one patient. Further, all subjects were under anesthesia at the time of lung resection, and this could have affected drug distribution, metabolism, and elimination. The surgical procedure itself also led to 3 distinct types of lesions, namely vascular, avascular, and mixed lesion. For vascular and mixed lesions, vascular tissue and/or spill-over blood from surgery could potentially affect the final concentrations measured; however, this is likely to be negligible due to the pulmonary artery being clamped during the procedure and the tissues briefly rinsed. Finally, patients underwent lung resection due to failing their TB treatment, which may have selected for subjects that had suboptimal lesion-specific drug exposure and introduced a bias in the type of lesions present.

In conclusion, our results suggest that target attainment is both lesion and drug specific. This suggests that the current paradigm, according to which all patients receive the same regimen at the time of TB diagnosis, could be revisited. The results show that cavities and lesions that contain caseum are the most difficult to treat, as their drug levels often do not reach adequate levels over the course of treatment. It is therefore important to take special precautions when treating patients with cavitation and monitor their outcomes closely. Stratifying patients based on disease extent, lesion types, and individuals’ drug-susceptibility profiles, followed by the rational selection of a tailored drug regimen, may lead to more adequate drug coverage of bacterial subpopulations residing in TB lesions. To that end, we provide an interactive tool that is able to estimate lesion-focused drug levels following any dose and schedule for these 7 drugs with the aim to support smarter design of regimens for clinical trial settings.

## Supporting information

S1 Study protocol (PDF)

S1 Table Number of observations for each lesion and drug. (DOCX)

S2 Table Plasma PK results compared to literature values. PK, pharmacokinetic
(DOCX)

S3 Table Rate of drug moving from plasma to lesion (KPL) measured in (h^−1^). KPL, rate parameter
(DOCX)

S1 Fig Representative examples of the 9 tissue types analyzed in this study. Samples were stained by HE and Mason’s trichrome for fibrosis. (A) HE staining of a lesion cluster including (1) uninvolved lung, (2) small cellular granuloma, (3) necrotic nodule, and (4) closed necrotic nodules with central caseum delineated by the white contour line, as indicated. (B) Mason’s trichrome staining of (5) large caseous (necrotic) fibrotic nodules and (6) highlighting of the fibrotic cuff in blue; (C) HE staining of a large cavity; most of the central caseum was lost upon sectioning. (7) Cavity wall delineates (8) remnants of caseum highlighted by the black contour lines. (D) HE staining of a collapsed cavity with fragments of a (9) fungal ball marked by black box and arrow. The hypha are shown in the inset above D. HE, hematoxylin–eosin.
(TIF)

S2 Fig Role of rate and ration on plasma profile. Plasma profile is shown in black and example of lesion profile shown in red. The KPL changes the profile on the time scale, while the RPL scales the concentration dimension of the profile. KPL, rate parameter; RPL, ratio parameter.
(TIF)

S3 Fig Visual predictive check of plasma over time to steady state. The visual predictive checks were simulated for 1,000 patients and are shown for all 7 drugs in plasma. The simulated concentration–time profiles are represented by a solid black line to represent the median at steady state, with the shaded light blue area representing the 95% prediction interval of the 1,000 patients simulated. The red line represents the median of plasma–time concentrations after the first dose of each respective drug. Open red circles represent drug concentrations of patients receiving their first doses, and black open circles represent observed data for patients who were at steady state at the time of resection. The dashed PK profiles serve to illustrate drug exposure increasing after each dose until reaching steady state. *PZA MIC of 12.5 mg/L is shown and is specific to an acidic environment less than pH 5.8. MIC, minimum inhibitory concentration; PK, pharmacokinetic; PZA, pyrazinamide.
(TIF)

S4 Fig Number of second-line drugs above MIC over time. Second-line drugs at steady state collected as the fifth percentile (low-exposure or at-risk patients) of 1,000 simulated patients over the 24-h dosing interval. Each square represents 1 h that a drug is above the MIC. The drugs are stacked for each hour with different colors representing different drugs. MIC, minimum inhibitory concentration.
(TIF)

S5 Fig Percentage PK/PD target reached using minimum bactericidal concentration compared to MIC. The percentage PK/PD efficacy target reached by a 1,000 simulated patient PK profiles as defined by their drug exposure versus drug MIC is shown in blue and MBC shown in yellow. Red boxes use CasMBC_90_ instead of MIC in the same set of simulated patients to compare that efficacy is both lesion and drug specific. CasMBC_90_, caseum-specific minimum bactericidal concentration; MIC, minimum inhibitory concentration; PK/PD, pharmacokinetic/pharmacodynamic.
(TIF)

S1 Data Plasma concentration data for KAN, CFZ, and LZD. CFZ, clofazimine; KAN, kanamycin; LZD, linezolid.
(XLSX)

## Abbreviations

AMK: amikacin
AUC: area under the concentration curve
AUG: amoxicillin/clavulanate
CasMBC_90_: caseum-specific minimum bactericidal concentration
CFZ: clofazimine
CLA: clarithromycin
CS: cycloserine
DS-TB: drug-sensitive TB
EC_90_: 90% maximal effective concentrations
EUCAST/CLSI: European Committee on Antimicrobial Susceptibility Testing/Clinical & Laboratory Standards Institute
IIV: interindividual variability
INH: isoniazid
KAN: kanamycin
LC/MS-MS: HPLC-coupled tandem mass spectrometry
LFX: levofloxacin
LOQ: limits of quantification
RPL: ratio from plasma to lesion
KPL: rate from plasma to lesion
LRT: likelihood ratio test
LZD: linezolid
MALDI-MSI: matrix-assisted laser desorption-ionization mass spectrometry imaging
MDR-TB: multidrug-resistant TB
MFX: moxifloxacin
MIC: minimum inhibitory concentration
NAT2: N-acetyltransferase 2
PAS: para-aminosalicylate
PD: pharmacodynamic
PK: pharmacokinetic
PTH: prothionamide
PZA: pyrazinamide
RIF: rifampicin
STM: streptomycin
TB: tuberculosis
V2: peripheral volume of distribution
CL: clearance
Cp: plasma concentration
ka: rate of absorption
Q: intercompartmental clearance
V: volume
XDR-TB: extensively drug-resistant TB

## References

[1] WHO | Report of the Technical Consultation on Advances in Clinical Trial Design for Development of New TB Treatments. WHO World Health Organization; 2018; Available from: https://www.who.int/tb/publications/2018/clinical_trail_design_TB_treatments/en/ (Accessed December 21, 2018)

[2] Jindani A, Harrison TS, Nunn AJ, Phillips PPJ, Churchyard GJ, Charalambous S, et al High-Dose Rifapentine with Moxifloxacin for Pulmonary Tuberculosis. N Engl J Med. Massachusetts Medical Society; 2014;371: 1599–1608. 10.1056/NEJMoa1314210 25337749 PMC4233406

[3] Gillespie SH, Crook AM, McHugh TD, Mendel CM, Meredith SK, Murray SR, et al Four-Month Moxifloxacin-Based Regimens for Drug-Sensitive Tuberculosis. N Engl J Med. Massachusetts Medical Society; 2014;371: 1577–1587. 10.1056/NEJMoa1407426 25196020 PMC4277680

[4] Merle CS, Fielding K, Sow OB, Gninafon M, Lo MB, Mthiyane T, et al A Four-Month Gatifloxacin-Containing Regimen for Treating Tuberculosis. N Engl J Med. Massachusetts Medical Society; 2014;371: 1588–1598. 10.1056/NEJMoa1315817 25337748

[5] Barry CE, Boshoff HI, Dartois V, Dick T, Ehrt S, Flynn J, et al The spectrum of latent tuberculosis: rethinking the biology and intervention strategies. Nat Rev Microbiol. Nature Publishing Group; 2009;7: 845 10.1038/nrmicro2236 19855401 PMC4144869

[6] Dartois V. The path of anti-tuberculosis drugs: from blood to lesions to mycobacterial cells. Nat Rev Microbiol. Nature Publishing Group; 2014;12: 159–67. 10.1038/nrmicro3200 24487820 PMC4341982

[7] Prideaux B, Via LE, Zimmerman MD, Eum S, Sarathy J, O’Brien P, et al The association between sterilizing activity and drug distribution into tuberculosis lesions. Nat Med. 2015;21: 1223–7. 10.1038/nm.3937 26343800 PMC4598290

[8] Pienaar E, Dartois V, Linderman JJ, Kirschner DE. In silico evaluation and exploration of antibiotic tuberculosis treatment regimens. BMC Syst Biol. 2015;9: 79 10.1186/s12918-015-0221-8 26578235 PMC4650854

[9] Rifat D, Prideaux B, Savic RM, Urbanowski ME, Parsons TL, Luna B, et al Pharmacokinetics of rifapentine and rifampin in a rabbit model of tuberculosis and correlation with clinical trial data. Sci Transl Med. American Association for the Advancement of Science; 2018;10: eaai7786 10.1126/scitranslmed.aai7786 29618565 PMC5969904

[10] Savic R, Weiner M, MacKenzie W, Engle M, Whitworth W, Johnson J, et al Defining the optimal dose of rifapentine for pulmonary tuberculosis: Exposure-response relations from two phase II clinical trials. Clin Pharmacol Ther. Wiley-Blackwell; 2017;102: 321–331. 10.1002/cpt.634 28124478 PMC5545752

[11] Mould DR, Upton RN. Basic concepts in population modeling, simulation, and model-based drug development-part 2: introduction to pharmacokinetic modeling methods. CPT pharmacometrics Syst Pharmacol. 2013;2: e38 10.1038/psp.2013.14 23887688 PMC3636497

[12] Savic RM, Jonker DM, Kerbusch T, Karlsson MO. Implementation of a transit compartment model for describing drug absorption in pharmacokinetic studies. J Pharmacokinet Pharmacodyn. 2007;34: 711–26. 10.1007/s10928-007-9066-0 17653836

[13] Kjellsson MC, Via LE, Goh A, Weiner D, Low KM, Kern S, et al Pharmacokinetic evaluation of the penetration of antituberculosis agents in rabbit pulmonary lesions. Antimicrob Agents Chemother. 2012;56: 446–457. 10.1128/AAC.05208-11 21986820 PMC3256032

[14] Guiastrennec B, Hooker AC, Olofsson A, Ueckert S, Keizer R, Karlsson MO. xpose: Diagnostics for Pharmacometric Models [Internet]. 2017 Available from: https://cran.r-project.org/package=xpose (Accessed March 18, 2019)

[15] Gumbo T. New susceptibility breakpoints for first-line antituberculosis drugs based on antimicrobial pharmacokinetic/pharmacodynamic science and population pharmacokinetic variability. Antimicrob Agents Chemother. American Society for Microbiology; 2010;54: 1484–91. 10.1128/AAC.01474-09 20086150 PMC2849358

[16] EUCAST. EUCAST. In: EUCAST [Internet]. 2015. Available from: http://www.eucast.org/ast_of_bacteria/ (Accessed March 18, 2019)

[17] Lavielle M. mlxR: Simulation of Longitudinal Data [Internet]. 2017. Available from: https://cran.r-project.org/package=mlxR (Accessed March 18, 2019)

[18] R Core Team. R: A Language and Environment for Statistical Computing [Internet]. Vienna, Austria; 2017. Available from: https://www.r-project.org/ (Accessed March 18, 2019)

[19] RStudio Team. RStudio: Integrated Development Environment for R [Internet]. Boston, MA; 2015. Available from: http://www.rstudio.com/ (Accessed March 18, 2019)

[20] Chang W, Cheng J, Allaire JJ, Xie Y, McPherson J. shiny: Web Application Framework for R [Internet]. 2017. Available from: https://cran.r-project.org/package=shiny (Accessed March 18, 2019)

[21] Werely CJ, Donald PR, van Helden PD. NAT2 polymorphisms and their influence on the pharmacology and toxicity of isoniazid in TB patients. Per Med. Future Medicine Ltd London, UK; 2007;4: 123–131. 10.2217/17410541.4.2.123 29788627

[22] Sarathy JP, Via LE, Weiner D, Blanc L, Boshoff H, Eugenin EA, et al Extreme Drug Tolerance of Mycobacterium tuberculosis in Caseum. Antimicrob Agents Chemother. American Society for Microbiology; 2018;62: e02266–17. 10.1128/AAC.02266-17 29203492 PMC5786764

[23] Lanoix J-P, Ioerger T, Ormond A, Kaya F, Sacchettini J, Dartois V, et al Selective Inactivity of Pyrazinamide against Tuberculosis in C3HeB/FeJ Mice Is Best Explained by Neutral pH of Caseum. Antimicrob Agents Chemother. American Society for Microbiology (ASM); 2016;60: 735–43. 10.1128/AAC.01370-15 26574016 PMC4750710

[24] Irwin SM, Gruppo V, Brooks E, Gilliland J, Scherman M, Reichlen MJ, et al Limited Activity of Clofazimine as a Single Drug in a Mouse Model of Tuberculosis Exhibiting Caseous Necrotic Granulomas. Antimicrob Agents Chemother. 2014;58: 4026–4034. 10.1128/AAC.02565-14 24798275 PMC4068578

[25] Jayaram R, Shandil RK, Gaonkar S, Kaur P, Suresh BL, Mahesh BN, et al Isoniazid pharmacokinetics-pharmacodynamics in an aerosol infection model of tuberculosis. Antimicrob Agents Chemother. 2004;48: 2951–2957. 10.1128/AAC.48.8.2951-2957.2004 15273105 PMC478500

[26] Lakshminarayana SB, Huat TB, Ho PC, Manjunatha UH, Dartois V, Dick T, et al Comprehensive physicochemical, pharmacokinetic and activity profiling of anti-TB agents. J Antimicrob Chemother. 2015;70: 857–67. 10.1093/jac/dku457 25587994 PMC7714050

[27] Kashuba AD, Nafziger AN, Drusano GL, Bertino JS. Optimizing aminoglycoside therapy for nosocomial pneumonia caused by gram-negative bacteria. Antimicrob Agents Chemother. American Society for Microbiology; 1999;43: 623–9. Available from: http://www.ncbi.nlm.nih.gov/pubmed/10049277 (Accessed March 18, 2019) 10049277 10.1128/aac.43.3.623 PMC89170

[28] Boak LM, Rayner CR, Grayson ML, Paterson DL, Spelman D, Khumra S, et al Clinical Population Pharmacokinetics and Toxicodynamics of Linezolid. Antimicrob Agents Chemother. 2014;58: 2334–2343. 10.1128/AAC.01885-13 24514086 PMC4023770

[29] Sarathy JP, Via LE, Weiner D, Blanc L, Boshoff H, Eugenin EA, et al Extreme Drug Tolerance of Mycobacterium tuberculosis in Caseum. Antimicrob Agents Chemother. American Society for Microbiology; 2018;62: e02266–17. 10.1128/AAC.02266-17 29203492 PMC5786764

[30] Heinrichs MT, Vashakidze S, Nikolaishvili K, Sabulua I, Tukvadze N, Bablishvili N, et al Moxifloxacin target site concentrations in patients with pulmonary TB utilizing microdialysis: a clinical pharmacokinetic study. J Antimicrob Chemother. 2018;73: 477–483. 10.1093/jac/dkx421 29186509 PMC5890684

[31] Jindani A, Harrison TS, Nunn AJ, Phillips PPJ, Churchyard GJ, Charalambous S, et al High-Dose Rifapentine with Moxifloxacin for Pulmonary Tuberculosis. N Engl J Med. Massachusetts Medical Society; 2014;371: 1599–1608. 10.1056/NEJMoa1314210 25337749 PMC4233406

[32] Gillespie SH, Crook AM, McHugh TD, Mendel CM, Meredith SK, Murray SR, et al Four-Month Moxifloxacin-Based Regimens for Drug-Sensitive Tuberculosis. N Engl J Med. Massachusetts Medical Society; 2014;371: 1577–1587. 10.1056/NEJMoa1407426 25196020 PMC4277680

[33] Drusano GL, Sgambati N, Eichas A, Brown DL, Kulawy R, Louie A. The Combination of Rifampin plus Moxifloxacin Is Synergistic for Suppression of Resistance but Antagonistic for Cell Kill of Mycobacterium tuberculosis as Determined in a Hollow-Fiber Infection Model. MBio. 2010;1: e00139-10–e00139-10. 10.1128/mBio.00139-10 20802826 PMC2925073

[34] Dooley K, Flexner C, Hackman J, Peloquin CA, Nuermberger E, Chaisson RE, et al Repeated Administration of High-Dose Intermittent Rifapentine Reduces Rifapentine and Moxifloxacin Plasma Concentrations. Antimicrob Agents Chemother. 2008;52: 4037–4042. 10.1128/AAC.00554-08 18765687 PMC2573112

[35] Reuter A, Tisile P, von Delft D, Cox H, Cox V, Ditiu L, et al The devil we know: is the use of injectable agents for the treatment of MDR-TB justified? Int J Tuberc Lung Dis. 2017;21: 1114–1126. 10.5588/ijtld.17.0468 29037291

[36] WHO | Rapid Communication: Key changes to treatment of multidrug- and rifampicin-resistant tuberculosis (MDR/RR-TB). WHO World Health Organization; 2018; Available from: https://www.who.int/tb/publications/2018/rapid_communications_MDR/en/ (Accessed March 18, 2019)

[37] Conradie F, Diacon AH, Everitt D, Mendel CM, van Niekerk C, Howell P, et al. Sustained high rate of successful treatment outcomes: interim results of 75 patients in the Nix-TB clinical study of pretomanid, bedaquiline and linezolid. Oral presentation at 49th World Conference on lung health of the International Union against tuberculosis and lung disease (The Union) October 25, 2018, The Hague, The Netherlands. The Hague; 2018. p. S69. Available from: https://www.abstractserver.com/TheUnion2018/TheUnion2018_Abstracts_Web.pdf (Accessed March 18, 2019)

[38] Colangeli R, Jedrey H, Kim S, Connell R, Ma S, Chippada Venkata UD, et al Bacterial Factors That Predict Relapse after Tuberculosis Therapy. N Engl J Med. 2018;379: 823–833. 10.1056/NEJMoa1715849 30157391 PMC6317071

[39] Imperial MZ, Nahid P, Phillips PPJ, Davies GR, Fielding K, Hanna D, et al A patient-level pooled analysis of treatment-shortening regimens for drug-susceptible pulmonary tuberculosis. Nat Med. Nature Publishing Group; 2018;24: 1708–1715. 10.1038/s41591-018-0224-2 30397355 PMC6685538

[40] Huang Q, Yin Y, Kuai S, Yan Y, Liu J, Zhang Y, et al The value of initial cavitation to predict re-treatment with pulmonary tuberculosis. Eur J Med Res. BioMed Central; 2016;21: 20 10.1186/s40001-016-0214-0 27154410 PMC4858857

[41] Kaplan G, Post FA, Moreira AL, Wainwright H, Kreiswirth BN, Tanverdi M, et al Mycobacterium tuberculosis growth at the cavity surface: a microenvironment with failed immunity. Infect Immun. 2003;71: 7099–108. Available from: http://www.ncbi.nlm.nih.gov/pubmed/14638800 (Accessed March 18, 2019) 10.1128/IAI.71.12.7099-7108.2003 14638800 PMC308931

[42] Kempker RR, Rabin AS, Nikolaishvili K, Kalandadze I, Gogishvili S, Blumberg HM, et al Additional drug resistance in Mycobacterium tuberculosis isolates from resected cavities among patients with multidrug-resistant or extensively drug-resistant pulmonary tuberculosis. Clin Infect Dis. 2012;54: e51–4. 10.1093/cid/cir904 22198790 PMC3284212

[43] Blanc L, Daudelin IB, Podell BK, Chen P-Y, Zimmerman M, Martinot AJ, et al High-resolution mapping of fluoroquinolones in TB rabbit lesions reveals specific distribution in immune cell types. Elife. 2018;7 10.7554/eLife.41115 30427309 PMC6249001

[44] Irwin SM, Prideaux B, Lyon ER, Zimmerman MD, Brooks EJ, Schrupp CA, et al Bedaquiline and Pyrazinamide Treatment Responses Are Affected by Pulmonary Lesion Heterogeneity in *Mycobacterium tuberculosis* Infected C3HeB/FeJ Mice. ACS Infect Dis. 2016;2: 251–267. 10.1021/acsinfecdis.5b00127 27227164 PMC4874602

[45] Sarathy JP, Zuccotto F, Hsinpin H, Sandberg L, Via LE, Marriner GA, et al Prediction of Drug Penetration in Tuberculosis Lesions. ACS Infect Dis. NIH Public Access; 2016;2: 552–63. 10.1021/acsinfecdis.6b00051 27626295 PMC5028112

