# Supplementary material for: Tuberculosis drugs’ distribution and emergence of resistance in patient’s lung lesions: A mechanistic model and tool for regimen and dose optimization
Source: PLoS Med. 2019 Apr 2;16(4):e1002773. doi: 10.1371/journal.pmed.1002773 (PMC6445413; doi:10.1371/journal.pmed.1002773)
Supplement: S1 Study protocol — (PDF) [file pmed.1002773.s001.pdf]

Study Protocol:

Measurement of Anti-TB Drugs in Lung Tissue From Patients Having Surgery to Treat Tuberculosis

NCT00816426

ABBREVIATIONS LIST

Abbreviation	Text
ADME	absorption, distribution, metabolism, and excretion
AE	adverse event
AFB	acid-fast bacilli
AI	Associate Investigator
ALT (SGPT)	alanine transaminase
AST (SGOT)	aspartate transaminase
AUC	area under the curve
β-hCG	human chorionic gonadotropin
BAL	bronchoalveolar lavage
BCG	bacillus Calmette-Guérin
CC	Clinical Center
CFP10	Culture Filtrate Protein 10
CFU	colony forming unit
CI	confidence interval
CNS	central nervous system
CRF	case report form
CSF	cerebro-spinal fluid
CV	curricula vitae
DHHS	Department of Health and Human Services
DIR	Division of Intramural Research
DSMB	Data and Safety Monitoring Board
DST	drug susceptibility testing
DTP	days to positivity
DVD	Digital Versatile Disc
EKG	electrocardiogram
ELF	epithelial lining fluid
ELISA	enzyme-linked immunosorbent assay
EOT	End of Therapy
ESAT-6	Early Secretory Antigenic Target 6
FDA	Food and Drug Administration
FDG	[^{18}F]-fluoro-2-deoxy-D-glucose
GCP	Good Clinical Practice
HIV	human immunodeficiency virus
HRCT	high-resolution computed tomography
ICH	International Conference on Harmonisation
IFN-γ	interferon gamma
IMS	Imaging Mass Spectrometry
INH	Isoniazid
IRB	institutional review board
ITRC	International Tuberculosis Research Center
IUD	intrauterine device
IV	Intravenous drug administration
KM	Kanamycin

**Abbreviation**	**Text**
ADME	absorption, distribution, metabolism, and excretion
LCID	Laboratory of Clinical Infectious Diseases
LCMS	liquid chromatography-mass spectrometry
MALDI	Matrix-assisted laser desorption/ionization
MCV	mean corpuscular volume
MDR-TB	multi-drug resistant tuberculosis
MIC	Minimum inhibitory concentration
MBC	Minimum bactericidal concentration
MPC	Mutation prevention concentration
MOHW	Ministry of Health and Welfare
MS	Mass Spectrometry
MTA	Material Transfer Agreement
MTB	*Mycobacterium tuberculosis*
NIAID	National Institute of Allergy and Infectious Diseases
NIH	National Institutes of Health
NITD	Novartis Institute of Tropical Diseases
NLME	non-linear mixed effects
OFX	Ofloxacin
OHRP	Office for Human Research Protections
OHSR	Office of Human Subjects Research
PBMCs	Peripheral blood mononuclear cells
PD	Pharmacodynamics
PI	Principal Investigator
PK	Pharmacokinetics
PO	Oral drug administration
PZA	Pyrazinamide
qd	Once daily
RBC	red blood cell
RCA	Research Collaboration Agreement
RIF	rifampin or rifampicin
SAE	Serious adverse event
SM	Streptomycin
SOC	Standard of Care
SPSS	Statistical Package for Social Sciences
SUV	Standardized Uptake Value
TB	tuberculosis
TBRS	Tuberculosis Research Section
TST	Tuberculin Skin Test
ULN	upper limit of normal
US NRC	United States Nuclear Regulatory Commission
WBC	white blood cell
WHO	World Health Organization
XDR TB	Extensively Drug-resistant Tuberculosis

Table of Contents

Measurement of Anti-TB Drugs in Lung Tissue From Patients Having Surgery to Treat Tuberculosis 1

1. Précis – Abstract 6

2 Study Objectives 6

2.1 Primary Objective 6
2.2 Secondary Objectives 6

3 Study Design and Methods 8

3.1 Design 8
3.2 Population to be Studied 8
3.3 Treatment Regimen and Treatment Period(s) 8
3.4 Procedures and Screening Tests 11
4.5 Study Agents 15

4 Subject Enrollment 18

4.1 Accrual 18
4.2 Eligibility Criteria 18
4.3 Determining Eligibility 20
4.4 Consent Methodology 20
4.5 Randomization 20
4.6 Withdrawal 20
4.7 Study Completion 20

5 An Analysis of the Study---Statistical Methods and Justification 21

5.1 Definition of Terms and PK Parameters Used in Primary and Secondary Endpoints, and in Statistical Analysis 21
5.2 Sample Size Justification 21
5.3 Outcome Measures 22
5.4 Statistical Methods for Data Analysis 23

6 Adverse Event Reporting Plan 24

6.1 Definition of an Adverse Event 24
6.2 Grading Adverse Events for Severity 25
6.3 Assessing Adverse Events for Relationship to Study 25
6.4 Adverse Event Documentation 26
6.5 Adverse Event Treatment 26
6.6 Potential Hazards 26
7.7 Study Sites AE Reporting 27
7.8 Study Sites SAE Reporting Requirements 27
7.9 Adverse Event Reporting to the NIAID, Asan, National Medical Center, and Pusan Institutional Review Boards and RCHSPB Safety 28

8 Data Management Plan---Data Collection, Sample Storage and Publication 31

8.1 Data Collection 31
8.2 Data Management 31
8.3 Data Storage 31
8.4 Publication of Research Findings 32
8.5 Sample Collection 32
8.6 Sample Storage 32

9 Protocol Monitoring Plan 33

10 Subject Protection 34

10.1 Rationale for Subject Selection ... 34
10.2 Participation of Children and Other Vulnerable Subjects ... 34
10.3 Risks/Benefits Analysis including Considerations of Alternatives to Participation ... 35
10.4 Privacy and Confidentiality ... 35
10.5 Remuneration ... 36

11 Pharmaceutical, Biologic, and Device Info ... 36

11.1 Formulation and Preparation of study drugs ... 36
11.2 Stability and Storage of Study Drugs ... 36
11.3 Incompatibilities of Study Drugs ... 36
11.4 Administration Procedures ... 36

12 References ... 37

12.1 Appendix I. Informed Consent Form ... 40

1. Précis – Abstract

It takes 6 to 24 months of intensive combination therapy to cure tuberculosis (TB) with antibiotics that have proven activity in vitro. In contrast, many pulmonary infectious diseases can be cured following single drug treatment with similar drugs for only one to a few weeks. We hypothesize that the unusual complexity of TB lesions and the degree of sequestration of TB bacilli within these lesions may limit access of the drugs to their site of action, leading to treatment failure, long treatment duration and the emergence of drug resistance. To test the hypothesis that drug maldistribution into lesions impacts on treatment duration and failure, we propose to examine the lesion-specific penetration properties of 5 standard anti-TB drugs in the lungs of subjects selected for lung surgery. The study is designed to understand what lesion types are the most difficult to penetrate. This aspect of TB drug pharmacokinetics has been largely neglected so far, probably owing to the lack of adequate technology and the limited availability of human TB lesion samples. Fifteen patients who elect lung resection surgery will be asked to participate in the study. Consented subjects will receive 5 first and second line anti-TB drugs concomitantly at 1 of 5 pre-determined times prior to surgery. At the time of resection, drug levels will be measured in plasma, in uninvolved lung tissue and in lesions using standard analytical methods as well as imaging Mass Spectrometry (MS) where drug concentration gradients can be visualized across tissue sections. The major aim of this study is to determine actual concentrations and permeability coefficients of the 5 study drugs in various lesion types contained within subjects' surgically removed lung tissue. Data analysis will also provide the **relative exposure** of each drug in plasma versus lung tissue and lesion. If conclusive, the results may be taken into consideration when selecting drug doses and dosing regimens. Additionally, images generated by standard of care (SOC) High Resolution Computed Tomography (HRCT), and Dynamic MRI for each subject will provide information regarding lesion structure and anatomy, lesional blood flow and microvascular function. Lesion-specific correlations will be established between CT radiology and drug pharmacokinetic (PK) to identify which histopathologic lesion types may be particularly difficult to sterilize and to evaluate the potential impact of drug penetration on treatment outcome. The long term goal of this study is to identify the factors behind poor lesion penetration, so that new agents can be optimized with better penetration properties to target 'harder-to-sterilize' lesion types.

2 Study Objectives

2.1 Primary Objective

The primary objective of this study is to compare the relative penetration efficiency of RIF and KM for large caseous necrotic nodules versus uninvolved lung, contained within surgically removed lung tissue of subjects receiving these drugs prior to lobectomy.

2.2 Secondary Objectives

1) To determine the absolute penetration efficiency of each of the five drugs in uninvolved lung tissue and in closed necrotic lesions and cavities

2) To compare the penetration of RIF, INH, PZA, MXF and KM into closed caseous necrotic lesions versus open cavities

3) To compare the levels of RIF and KM in plasma versus caseous necrotic lesions over time

4) To compare the levels and exposure of all study drugs in plasma, normal lung and lesions

5) To calculate classical PK/PD indices in plasma and lesions, in relation to minimum inhibitory concentration (MIC), minimum bactericidal concentration (MBC) and mutation prevention concentration (MPC), for future regimen optimization

6) To correlate lesion concentrations of the 5 study drugs with lesion type, as determined by histopathology and HRCT, and to identify lesion-specific patterns of drug penetration kinetics

7) To correlate lesion concentrations of the 5 study drugs within each lesion type, and to identify drug-specific patterns of penetration kinetics

8) To correlate lesion type and structure, as determined by histological microscopic analysis, with radiologic images

9) To visualize and quantify concentration gradients of the five study drugs within the different layers of closed lesions, using scanning mass spectrometry across tissue sections

10) To assess vascular structure, endothelial function and perfusion around individual lesions, using Dynamic (contrast enhanced) MRI

3 Study Design and Methods

3.1 Design

This is a single-dose pharmacokinetic study of 5 concomitant drugs where resected lung tissue will be recovered to determine and compare drug levels within plasma, sputum, healthy lung and pulmonary TB lesions over a 24h period. Comparison and modeling of the concentration-time profiles in plasma and lesions are used to infer permeability coefficients, exposure ratios and concentration gradients for five major 1st and 2nd line TB drugs: RIF, INH, PZA, KM and MXF. Subjects will be randomized to receive the 5 drugs at one time point, either at 2, 4, 8, 12 or 24h prior to surgery, or as close as practical to those times with careful recording of the time of administration. Enrollment will continue until evaluable tissues from 3 subjects have been processed at each assigned time point. Blood and sputum samples will be obtained just before drug administration (trough), and blood samples will also be obtained at 2h (C_{max}) and 6h post-dosing or as practicable. Whenever possible, one additional blood and/or sputum sample will be obtained between drug dosing and surgery. Finally, blood will be collected at the time of central line placement and sputum at the time of intubation prior to surgery. The time of each blood draw and sputum collection will be recorded as carefully and accurately as possible. Urine will be systematically collected between the time of drug administration and surgery. The removed tissue will be dissected to separate lesion material from uninvolved lung. The location of each lesion within the lung lobes will be recorded. Each lesion will be measured and weighed, then further divided to perform the following analyses: determination of overall lesional drug concentrations by liquid chromatography-mass spectrometry (LCMS), imaging mass spectrometry and histopathology analyses to visualize 2D drug distribution within lesions. A pre-operative dynamic MRI scan (30-60 min duration) will be conducted one to two weeks prior to surgery to access lesional perfusion and endothelial function. This procedure can be performed with most MRI machines in clinical use and this may be taken at a different site. A scan will be obtained before injection of the contrast agent (gadolinium). After the contrast agent is given, the subject will be scanned serially at several time points up to 8 minutes post-injection. The rate of gadolinium uptake and its retention properties will be determined using software. The entire scan will take about 45 minutes. A subject will participate in this study from entry (1 to 2 weeks prior to surgery), through surgery and for 48h post surgery.

3.2 Population to be Studied

The study population will be drawn from subjects with pulmonary tuberculosis who elect to undergo lung resection at (i) the Asan Medical Center, Seoul, (ii)Pusan National University Hospital, Busan, Republic of Korea , , and (iii) National Medical Center, Seoul, Republic of Korea.

3.3 Treatment Regimen and Treatment Period(s)

Five groups of 3 subjects will each receive one concomitant dose of all 5 study drugs according to the doses and routes presented below. Study drugs will be administered 2, 4, 8, 12 and 24h prior to surgery. For subjects who will be on one or more of the study drugs as part of their

ongoing treatment regimen, the time of administration will be adjusted to meet the requirements of the study design. For subjects whose drug regimen already contains an aminoglycoside other than KM, this aminoglycoside will be substituted with KM, provided that the subject consented to the substitution.

Doses:
- Rifampicin (RIF) will be dosed orally at 600mg or 450mg for subjects under 50kg of bodyweight;
- Isoniazid (INH) will be dosed orally at 300mg;
- Pyrazinamide (PZA) will be dosed orally at 1.5g;
- Moxifloxacin (MXF) will be dosed orally at 400mg;
- Kanamycin (KM) will be dosed intramuscularly at 1g or 750mg for subjects under 50kg of bodyweight

The 5 groups of subjects are distinguished by the timing of the drug administration, determined by randomization. The timing will be one of 24, 12, 8, 4, and 2 hours prior to estimated vessel and bronchus ligation time, which takes place at varying times after the start of surgery, depending on the extent of adhesions between the pleural membrane and the lung lobes. These times may vary slightly due to constraints inherent to the surgical procedure. Therefore, the exact timing of vessel and bronchus ligation relative to drug administration will be recorded accurately. Ligation marks the time of isolation between tissue compartment and plasma compartment, and will therefore be used as the time post-dosing in the PK analysis.
We will allow the following deviations from assigned timing between drug administration and vessel ligation:
- minus 1h and plus 3h for the 2h group
- minus 2h and plus 4h for the 4h group
- minus 4h and plus 6h for the 8h group
- plus or minus 6h for the 12h group
- plus or minus 12h for the 24h group

In cases where these maximum deviations are exceeded, samples will be collected and analyzed for drug content, but the data will be marked as falling outside allowable time points. To compensate for these potential fall-outs, enrollment will continue until evaluable tissues from 3 subjects falling within the allowable window are obtained in each group. Two statistical analyses will be conducted, one including only those subjects in compliance with the protocol, and one including all subjects. Both sets of results will be interpreted in light of the cause(s) for delay.

3.3.1 Study Schema

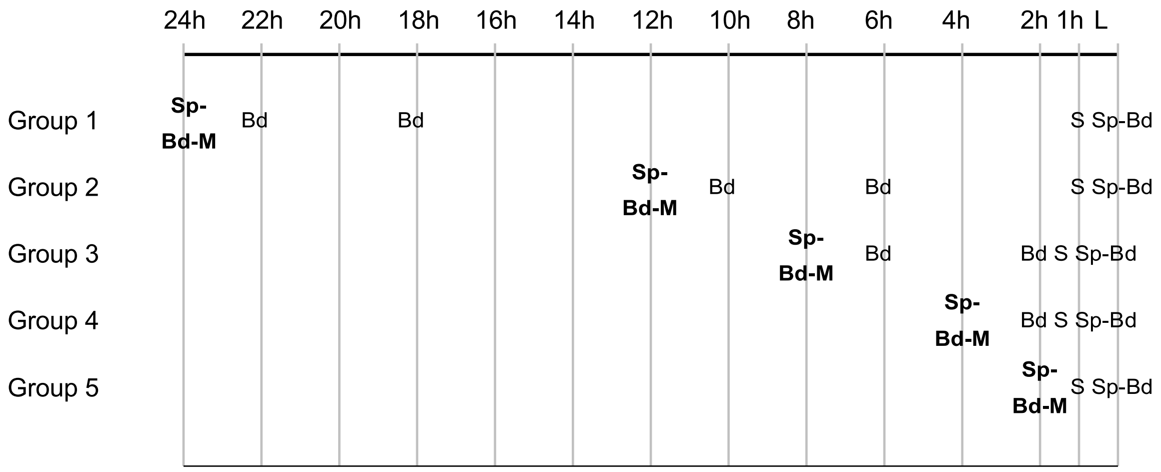

L: estimated ligation time or T_0 relative to drug administration

S: surgery starts – including blood sampling and sputum collection upon intubation

M: drug administration

Bd: blood draw for PK (2ml)

Sp: sputum sampling for PK and/or bacteriology

3.3.2 Study Time Line

Within 14 days prior to surgery

- Consent
- Pregnancy test if female
- Randomization
- Dynamic MRI to quantify lesional perfusion and endothelial function

Two weeks to one day prior to surgery

- Sputum collection for bacteriological analysis

24h to 2h prior to surgery

- Subject will receive the 5 study drugs **<u>once</u>**, as shown below.
- Doses:
  - RIF 600mg PO (450mg if <50kg);
  - INH 300mg PO
  - PZA 1.5g PO
  - KM 1g IM (750mg if <50kg)
  - MXF 400mg PO

Drug administration: 24, 12, 8, 4, or 2h (as randomized) prior to estimated vessel and bronchus ligation time, which takes place at varying times after the start of surgery, depending on the complexity of the surgical procedure (see section 4.3 above for allowable deviations from assigned time points). For subjects whose drug regimen already contains an aminoglycoside other than KM, this aminoglycoside will be substituted with KM, provided that the subject consented to the substitution.

Blood sampling for PK: just prior to drug administration, 2h post-dosing (except for group 5), 6h post-dosing (except for group 4 & 5), at anytime between drug dosing and surgery (optional), at the time of central line placement and at the time of vessel and bronchus ligation. While exact timing of sampling can deviate from the recommended time points, accurate recording of the sampling time is critical to the success of the study.

Sputum sampling prior to surgery: Sputum will be collected immediately before drug administration (1 mL or more) with accurate recording of each production time, only if the subject is productive. One additional sputum sample will be collected at any time between drug administration and surgery, with accurate recording of production time.

Urine sampling

From the time of drug administration to the time of surgery, urine will be collected to determine drug clearance and support PK modeling.

Sputum sampling during surgery

Sputum will be sampled after endotracheal intubation using a suction catheter with a sputum trap as close as possible to the time of ligation. Optionally, broncho-alveolar lavage (BAL) fluid may also be obtained at the time of ligation by passing a bronchoscope through the endotracheal tube.

3.4 Procedures and Screening Tests

Tests that have been completed within 15 days *prior* to study entry can be reviewed and used to determine eligibility. (This will be done after subject consent.) In such a situation, entry evaluation tests do not have to be repeated.

3.4.1 Study Evaluations at Entry (After Consent)

1) Pregnancy test for female subjects who are of child bearing age
2) A clinical examination which will consist of a physical examination, including body temperature, pulse, blood pressure, respiratory rate, weight, signs and symptoms of tuberculosis
3) Medical history will be taken at enrollment to include any previous diagnoses of major organ systems
4) Sputum smear microscopy and TB culture

5) Medication history will include a complete history of any prescription or non-prescription medications taken, including actual or estimated start and stop dates; allergies to any medications and their formulations will be documented
6) A baseline Dynamic MRI scan
7) Sputum, urine, and blood collections for experimental tests (in Diagnostic/Laboratory Procedures)
8) EKG with recording of QTc interval

3.4.2 Diagnostic/Laboratory Procedures

Pregnancy Testing
A 10 mL to 50 mL sample of urine will be collected for pregnancy testing at study entry and before each radiological imaging scan by commercial human chorionic gonadotropin (β-hCG) determination kit.

Mycobacterial Testing Procedures
Within 2 weeks prior to surgery, approximately 3 mL to 30 mL of sputum will be collected in each of 1-3 sterile containers for AFB smear and culture (quantitative solid and liquid culture methods) and molecular testing. If subjects cannot expectorate, sputum may be obtained via induction with hypertonic saline as previously described [35]. A sputum booth, if available, will be used. In addition, if a prior culture for the subject has been archived, a sample of this isolate will be collected for molecular characterization.

AFB smears will be scored by the World Health Organization's (WHO) standard scale. (Laboratory services in tuberculosis control Part II, 1998. Microscopy Document WHO/TB 98.258. World Health Organization, Geneva, Switzerland.)

The bacterial isolate or sputum will be tested for species identification and the results of previously ordered DST tests may also be collected, recorded on the CRF in subject history, and banked and stored at ITRC or Yonsei University. These samples may be shipped to NIAID or UMDNJ for additional testing.

Dissection of nodules and cavities from resected lung tissue
The dissection of resected tissue will follow established SOPs for determination of drug concentration in lesions and is described here briefly. All specimen collection tubes are prelabeled and weighed to speed the dissection process.

Prior to surgery, the CT will be reviewed by the pathologist and study team to form a dissection plan. Classes of tissue to be specifically collected are: 1) several regions of apparently normal tissue, 2) large caseous tuberculomas ($\geq$ 0.5 cm), 3) cavity contents, 4) cavity walls including fibrotic tissue layer 6) fibrotic granulomas, and 7) small nodules.

Each lesion collected will be classified, measured for size (length, thickness and depth will be recorded in order to calculate surface-to-volume ratio for PK modeling) and pH, weighed, and recorded on the dissection log describing the disposition of the tissue. All lesions will be divided in this priority as follows and described in the dissection SOP.
1) Tissue frozen in liquid nitrogen or dry ice for drug extraction

2) Tissue frozen in liquid nitrogen vapor on tissue trays for Imaging Mass Spectrometry and histology
3) Tissue in formalin fixation solution for histology
4) Any remaining tissue frozen in liquid nitrogen for back-up

All lesions will have at least one sample that may be examined by normal histology methods, including hematoxylin and eosin staining of either formalin fixed or frozen sections. In addition, sections will be stained for acid fast bacilli and CD31 or CD34 (vessels). Larger lesions (0.8 cm or greater) will provide tissue for assessment of bacterial burden, bacterial genome equivalence, bacterial gene expression, and lesion cellular content using standard laboratory procedures.

Pharmacokinetic profiling of the study drugs

Approximately 2 mL of blood will be collected in heparinized tubes at several time points between drug administration and vessel ligation, to measure the concentrations of the study drugs and evaluate drug absorption and systemic exposure. The timepoints will be (i) trough (immediately before drug administration), (ii) average C_{max} or peak (2 hours after drug administration), (iii) end of absorption (6h after drug administration), (iv) time of central line placement before anesthesia, and (v) T_0 or time of ligation (L) of the vessels and bronchus. One additional blood sample may be collected at any time between drug administration and ligation with accurate recording of sampling time. The group of subjects receiving the study drugs 2 hours prior to surgery will only provide blood samples at trough, time of central line placement and T_0. The group of subjects receiving the study drugs four hours prior to surgery will provide blood samples at trough, C_{max}, time of central line placement and T_0. These procedures are outlined in the Study Schema. While exact timing of sampling can deviate from the recommended time points, as outlined in section 4.3, careful and accurate recording of the sampling time is the key to the success of the study.

Sputum will be collected before drug administration (1 mL or more) with accurate recording of each production time. Whenever possible, small sputum sample(s) (1 mL or more), spontaneously produced by subjects, will be obtained at any time between drug administration and surgery, with careful recording of the time of production. If these samples are not available due to lack of production, it will not be considered a failure in protocol execution. At the time of surgery, sputum will be sampled after endotracheal intubation using a suction catheter with a sputum trap. All samples should be stored in a below -70 ℃ freezer as quickly as possible after collection.

Urine will be collected from drug administration and throughout surgery, with accurate recording of time and volume. Creatinine and drug levels will be measured to estimate renal elimination throughout the study period as well as the true clearance of drugs that are minimally metabolized such as KM.

These blood, urine and sputum samples will be processed at ITRC or Yonsei University College of Medicine and approximately 0.5 to 1 mL of plasma and sputum will be transferred to and analyzed at UMDNJ to determine the concentrations of RIF, INH, PZA, KM, MXF, and other 2nd line drugs which the subjects will be taking concomitantly.

Determination of drug levels in uninvolved lung tissue and lesion homogenates by LCMS

Aliquots of approximately 0.2g (0.1g to 0.25g with accurate recording of the weight to facilitate further processing) of lung and lesion material will be homogenized and processed according to the protocol provided by NITD. Homogenized samples will be mixed with a suitable volume of organic solvent based on indicated weight. The organic phase, containing small molecule drugs, will be evaporated to dryness. These lyophilized samples will be shipped to and analyzed by UMDNJ for the absolute quantification of study drug levels as well as concomitantly administered non-study drugs.

Determination of drug distribution into normal lung tissue and lesions by imaging Mass Spectrometry

Lesion and lung sections will be subjected to 3Mrad γ-irradiation to sterilize them prior to analysis by scanning MALDI-TOF. Slices of frozen tissue samples (~12-20 ∝m thick) from each different lesion type will be mounted on flat metallic target plates. Histological analysis of alternating slices will direct the placement of matrix drops onto specific cells with high placement accuracy effected by a robotic micro-dispenser. Processing digital images of the spotted plate will provide relative locations of each matrix spot. These coordinates will then be transferred and registered to the mass spectrometer for automated data acquisition. The heterogeneity of drug distribution is visualized by the rate of change in brightness or color of the output file. The resolution (~50-500∝M) is user-defined, within instrumentation or other limitations. Ion distribution is then superimposed onto the histology image, to co-register with the relative density and precise location of the analytes in the tissue/granuloma of interest.

Detection of genetic polymorphisms of hepatic and enteric N-acetyltransferases (NAT1 and NAT2) and other polymorphisms related to TB drug metabolism

Genetic analysis of the genes will be done by isolating DNA from whole blood or tissue. The human DNA will be amplified by using a series of specific primers that span the regions known to confer the difference in rapid and slow acetylators of INH, and Rifampicin metabolism (CYP3A4) as examples. The PCR products will be sequenced and the results compared with known sequences to classify the patients into categories.

3.4.3 Diagnostic Radiation

There is no research radiation on this protocol. X-rays and CT scans will be performed as SOC at each hospital.

Sites responsible for data generation and analysis

Procedure	Responsible Site(s)	Location
Microbiology (resistance detection by DST & molecular methods, AFB smear and culture, genotyping)	Yonsei University<br>ITRC<br>AMC, PNUH, NMC	Seoul, Korea<br>Seoul/Changwon, Korea<br>Busan, Korea
Histology (formalin)	ITRC	Seoul/Changwon, Korea

Histology and IHC for vessels (frozen sections)	NIH	Bethesda, MD
Drug quantification by standard MS	UMDNJ	New Jersey
2D distribution of drugs by imaging MS	Novartis Institute for Biomedical Research (dept of “Analytical Sciences”)	Basel, Switzerland
Data analysis and modeling	Novartis Institute for Biomedical Research (Dept of “Modeling and Simulation”)	Basel, Switzerland

4.5 Study Agents

Rifampicin or **rifampin** (RIF) is a semi-synthetic compound derived from *Amycolatopsis rifamycinica*. It is mostly used in combination to treat TB, while other disease indications include brucellosis, leprosy, legionnaire's disease and problematic drug-resistant staphylococcal infections. RIF inhibits DNA-dependent RNA polymerase in bacterial cells by binding its beta-subunit, thus preventing transcription to RNA. Its MIC against replicating TB bacilli is 0.1∝g/ml and its minimum bactericidal activity (MBC) is 0.5∝g/mL. It is one of the rare anti-TB drugs with some activity against non-replicating cells.

The RIF molecule is quite lipophilic and hence is well and rapidly absorbed following oral administration, though high-fat meals may delay or decrease absorption. Although this phenomenon has not proven entirely consistent and reproducible, many studies have reported a negative effect of co-administered INH on the oral exposure of RIF in mice and in TB subjects [28, 36, 37]. Studies in mice indicate that INH interferes with the absorption of RIF thereby decreasing its bioavailability. In the study by McIlleron *et al.*, [37] it was observed that formulation was a contributing factor to this phenomenon, hence the lack of reproducibility across studies [38, 39].

RIF is thought to have good intracellular penetration despite its high molecular weight. The recommended daily dose is 10mg/kg up to 600mg, PO or IV, though it is unclear whether this dose was originally selected based on experimentally obtained PK/PD data. A recent study conducted in South Africa [40] demonstrates that RIF maintains a linear dose response of early bactericidal activity up to 1200mg, suggesting that the drug may be underdosed at 600mg daily. It is prepared alone or in combination and commercially available from numerous sources.

Rifampicin induces mainly cytochromes CYP3A4 and CYP2C9 and therefore reduces blood concentrations of drugs which are metabolized by these enzymes, among which anti-HIV drugs (Indinavir, Efavirenz, Nelfinavir, Saquinavir) and antifungals (Fluconazole, Itraconazole, Ketoconazole, Terbinafine).

Isoniazid (INH) is a first-line antituberculous medication discovered in 1952 and used in the prevention (alone) and treatment (in combination) of tuberculosis. Isoniazid is a prodrug and

must be activated by a bacterial catalase to inhibit the synthesis of mycolic acids in the mycobacterial cell wall. Consequently, INH is bactericidal to rapidly-dividing mycobacteria, with an MIC of 0.05∝g/ml and an MBC of 1∝g/ml, but is inactive if the mycobacterium is non-replicating or slow-growing.

INH is well absorbed following oral or intramuscular administration, though peak plasma concentrations are significantly affected by concomitant fatty food intake. It is recognized to have good penetration in inflamed meninges and caseous granulomas. The recommended daily dose is 5mg/kg up to 300mg, PO or IV.

INH is metabolized in the liver mostly via acetylation by the NAT-1 and NAT-2 acetyltransferases. The genetic diversity of these enzymes in humans has a marked influence on isoniazid metabolism, resulting in either toxic levels in slow acetylators or subtherapeutic levels in fast acetylators. There exist numerous polymorphisms of this gene that determine the rate of drug acetylation. Major genotypes have recently been grouped into three metabolic phenotypes: fast acetylators (FF), intermediate acetylators (FS), and slow acetylators (SS). A recent study conducted with Japanese volunteers concluded that the proper daily dose for rapid acetylators may be 1.5-times higher than that currently recommended [41]. The proportion of rapid acetylators in the Korean population has not been reported. It is estimated to be greater than 40% [42] and up to 90% [43] in the Japanese population, as opposed to the 40% figure observed among Caucasians and which was used to establish the conventional dose of 300mg daily.

Pyrazinamide (PZA) is a synthetic derivative of nicotinamide requiring activation by the mycobacterial enzyme pyrazinamidase, only active under acidic conditions which are thought to be found within the phagolysosomal compartment of macrophages. The conversion product, pyrazinoic acid, inhibits fatty acid synthetase I, required by the bacterium to synthesize fatty acids, though this has been disputed. It has an MIC of 6 ∝g/ml and is not cidal under *in vitro* conditions. Overall, its mechanism of action and reasons for good sterilizing activity *in vivo* are poorly understood. It is part of the 4-drug combination recommended by the WHO to treat drug- sensitive tuberculosis and is also included in most second-line regimens.

PZA is completely absorbed orally and only available as an oral drug, with a recommended daily dose of 20 to 25 mg/kg up to 2 grams. No significant effect of concomitant PZA has been observed on the exposure of 1st line TB drugs.

Little is known about the penetration of PZA into tissues. In a study where PZA levels were measured in tuberculous pleural effusion and psoas abscess, intralesional PZA concentrations were 5 to 10-fold lower than serum levels and below the MIC 2h post dose [44]. In contrast, PZA levels in cerebro-spinal fluid (CSF) were similar to those found in plasma, both in rabbits and in humans [45, 46].

Kanamycin (KM) is an aminoglycoside antibiotic belonging to the same class of drugs as Streptomycin, one of the first drugs used to treat TB in the 50's. It kills sensitive bacteria by binding to the 30S ribosomal subunit and interfering with protein synthesis. Its MIC and MBC against MTB are 2 and 6 ∝g/mL, respectively, with a remarkably low MBC/MIC ratio. However, KM is only used to treat serious bacterial infections due to severe renal toxicity and ototoxicity. No interaction with the metabolism of other drugs has been reported. The drug is approved by

the Korean Ministry of Food and Drug Safety (MFDS) but not the US FDA for use against pulmonary TB.

Aminoglycosides do not concentrate effectively in tissues and remain mostly extracellular, in agreement with their hydrophilic nature and low cLogP (Table 1). Accordingly, KM is not orally absorbed and is administered via the intramuscular or intravenous route at a dose of 1g daily or 750mg for subjects under 50kg of bodyweight. The epithelial lining fluid (ELF):plasma ratio of aminoglycosides used against TB ranges from <0.3 for streptomycin to <1.0 for KM. However, receptor-mediated endocytosis of aminoglycosides, by the two main receptors megalin and cubilin found at the surface of epithelial cells, plays an important role in the accumulation of aminoglycosides in renal proximal tubules [47]. Megalin has also been found on the surface of type II pneumocytes, which are critical for the establishment of mycobacterial pulmonary infections as a portal of mycobacterial organisms to the lungs.

The PK/PD parameter driving aminoglycoside efficacy is the ratio between peak concentration and MIC (C_{max}/MIC), with a maximum effect at C_{max}/MIC around 10-12. Aminoglycosides have a long post-antibiotic effect, which can be described as an ability to inhibit bacterial growth well after the plasma/tissue levels have fallen below the MIC.

Moxifloxacin (MXF) is a synthetic fluoroquinolone antibiotic. It inhibits bacterial topoisomerase II (DNA gyrase) and topoisomerase IV, which are essential enzymes playing a crucial role in the replication and repair of bacterial DNA. Its MIC, MBC and intracellular activity against MTB are 0.5, 2 and 1 ∝g/mL, respectively, with again a low MBC/MIC ratio.

Moxifloxacin is used to treat a variety of respiratory infections, as well as skin and skin structure infections. Moxifloxacin is also used for the treatment of complicated intra-abdominal infection. The usual dosage is 400 mg daily taken orally or via intravenous infusion over 1 hour. Although it is increasingly used as second line agent in MDR-TB cases, the drug is not formally approved by the MFDS or the USFDA for use against pulmonary TB.

MXF is rapidly absorbed following oral administration, reaching a mean peak drug plasma concentration within 2 h after a 400 mg dose. The rate and extent of absorption are not significantly affected by food. Moxifloxacin binds weakly to plasma proteins and penetrates well into most tissue and fluid compartments, with generally higher drug concentrations in tissues than in plasma, and good intracellular penetration [3, 48]. Of the various fluoroquinolones considered for the treatment of tuberculosis, MXF exhibits the highest ELF:plasma ratio with a value of ~6 and a 20 to 70-fold accumulation in alveolar macrophages [49].

Moxifloxacin undergoes phase II metabolism in the liver to produce sulphate and glucuronide conjugates. Induction of conjugative enzymes by RIF has not been thoroughly investigated but some effect on MXF metabolism cannot be excluded since RIF was shown to induce sulphate conjugation of one oral contraceptive [50].

Table 1. Physico-chemical Properties of the 5 Study Drugs

drug	isoniazid	kanamycin	moxifloxacin	pyrazinamide	rifampicin
clogP	-0.668	-5.173	-0.082	-0.206	3.71
mol weight	137.14	484.51	401.44	122.13	822.96

CMR*	3.714	11.153	10.464	3.343	21.925
PSA (square Å)**	68.01	282.61	86.27	55.98	220.15
volume (cubic Å)	126.75	473.28	366.49	113.27	847.56
pKa	3.27 (amine)	9.90/8.22/7.24/6.57 (amines)	9.77 (amine)/3.05 (COOH)	3.53 (amine)	na
H-bond acceptors	4	15	7	3	16
H-bond donors	3	15	2	2	6
amide group	1	0	0	1	1
rotatable bonds	2	6	4	1	5
flexibility index	14.58	12.38	9.96	8.19	6.08
logD 7.4	-0.7	-8.6	-0.1	-0.2	na
logPm	-2.29	-6.55	-4.78	-2.29	-2.39
water solubility	2	1	2	1	3

* CMR: Computed Molar Refractivity
** PSA: Polar Surface Area

4.5.1 Other Agents

While on study drugs, subjects will also be receiving 2nd line agents determined by DST results as part of their ongoing regimen. The possible drugs used include but are not limited to prothionamide, para-amino salicylic acid (PAS), ofloxacin, levofloxacin, and cycloserine There are no known interactions between these drugs and the study drugs. Subjects having received rifampin or rifabutin over the past 30 days will be excluded from the study.

4 Subject Enrollment

4.1 Accrual

Subjects with diagnosed pulmonary TB who have elected to undergo lung lobectomy will be given the opportunity to participate in the study. We expect accrual to take approximately 12 months among the three study centers of Asan Medical Center, National Medical Center, and Pusan National University Hospital.

4.2 Eligibility Criteria

4.2.1 Inclusion Criteria

1) Males and Females age 20 and above
2) Selected for lung resection due to anti-tuberculous treatment failure, multidrug resistant disease, or other reason determined by the treating physician
3) Radiographic evidence of tuberculous disease of the lung(s)
4) If already on an aminoglycoside, ability and willingness to substitute this aminoglycoside with KM for the one study dose
5) Willingness to receive MRI scan and marker and Gadolinium injection
6) Willingness to have samples stored
7) Ability and willingness to give written or oral informed consent
8) EKG without evidence of QT* prolongation within 1 week of study.

* QT prolongation is considered when the QTc interval is greater than 440 ms (men) and 460 ms (women), although arrhythmias are most often associated with values of 500 ms or more.

4.2.2 Exclusion Criteria

1) Subjects < 20 years of age
2) Women of childbearing potential, who are pregnant, breast feeding, or unwilling to avoid pregnancy (*i.e.*, the use of appropriate contraception including oral and subcutaneous implantable hormonal contraceptives, condoms, diaphragm, intrauterine device (IUD), or abstinence from sexual intercourse) [Note: Prospective female participants of childbearing potential must have negative pregnancy test (urine) within 48 hours prior to study entry.]
3) Allergy or hypersensitivity to any of the 5 study drugs or any fluoroquinolone, aminoglycoside, or rifamycin
4) Those with severe gout
5) Severe claustrophobia or Gadolinium hypersensitivity (tbc)
6) Renal, hepatic, auditory and/or vestibular impairment.
   a. Serum creatinine > 2.0 mg/dL (renal)
   b. Aspartate aminotransferase (AST or SGOT) >100 IU/L (LFTs)
   c. Alanine aminotransferase (ALT or SGPT) >100 IU/L(LFTs)
   d. Total bilirubin > 2.0 mg/dL(LFTs)
7) Documented QT prolongation
8) The use of any of Rifampicin (RIF), Rifapentine or Rifabutin within 30 days prior to the study
9) HIV infection, determined by a positive HIV test performed with the past 6 months
10) The use of any of the following drugs within 30 days prior to study
    a. Systemic cancer chemotherapy
    b. Chronic systemic corticosteroids (oral or IV only) with the following exceptions (i.e., the following are NOT exclusion criteria): intranasal, topical, and inhaled corticosteroids, a short course (10 days or less) of corticosteroids for a non-chronic condition completed at least 2 weeks prior to enrollment in this study
    c. Systemic IND agents other than linezolid
    d. Antiretroviral medications
    e. Growth factors
11) The need for ongoing therapy with warfarin, phenytoin, lithium, cholestyramine, levodopa, cimetidine, disulfiram, ergot derivatives, fosphenytoin, carbamazepine, cyclosporine, tacrolimus, sirolimus, amiodarone or phenobarbital (If a potential subject is on one of these medications but it is being stopped per standard of care, to be eligible for the study the drug must be stopped at least one day prior to receiving study drug. A longer washout period is not necessary.) The only exception to this is amiodarone; because of amiodarone's long half-life and potential for QT prolongation, it should be stopped at least 60 days prior to receiving study drugs.

4.3 Determining Eligibility

Evaluations to determine eligibility will be reviewed within 15 days prior to study entry unless otherwise specified. Referring doctors will use data from normal hospital admission tests and medical history to evaluate potential eligibility of patients. Doctors in the hospital will invite potentially eligible patients to participate.

4.4 Consent Methodology

Consent will be obtained prior to any additional testing or sample collection needed for the study. For women of childbearing potential, a urine pregnancy test will be performed prior to the other laboratory evaluations. The consent forms will be prepared in Korean and back translated into English to check the content. As the majority of the patient populations from which the study subjects are drawn are literate, written consent will be obtained. Oral consent will be obtained in the case of illiterate or blind/vision disturbed subjects with the written consent being presented orally by the investigator (principal investigator or subinvestigator) and witnessed by an impartial third party. The subject will be asked to sign the form or thumb-print the form if unable to sign. A witness signature and date is only required for subjects who are presented the consent orally. The Investigator (principal investigator or subinvestigator), a trained member of the study team, will sign the form also. A copy of the consent form will be given to the subject. Consent forms will be stored in the subject study binders containing the case report forms.

4.5 Randomization

Subjects will be assigned to one of the 5 drug administration times by simple randomization. A single balanced list of ID numbers with the 5 drug dose times permuted randomly across the IDs will be provided by the statistician. S/he will provide the randomized list to the centralized monitoring organization who will manage the randomization assignments for the 3 active sites. Each time a subject consents and is enrolled, a designee at that site will contact the monitoring organization who will then assign the study ID and drug dosing time for the subject by assigning the next available ID on the list.

4.6 Withdrawal

Subjects who are consented to participate in the study and do not follow through with surgery, are found to be ineligible after signing the consent form, do not have an evaluable primary endpoint or request to be withdrawn will be withdrawn from the study. If the investigator believes that the study is no longer in the best interest of the subject, the subject may also be withdrawn from the study at the investigator's discretion. If possible, subjects who are withdrawn will be replaced.

4.7 Study Completion

A subject will have completed the study 48 hours post surgery (vessel ligation). If a subject withdraws or is withdrawn, for any reason, before lung tissue has been transferred to the dissection team, his/her participation in the study will be completed.

5 An Analysis of the Study---Statistical Methods and Justification

5.1 Definition of Terms and PK Parameters Used in Primary and Secondary Endpoints, and in Statistical Analysis

In this study, the primary endpoint will be the relative **permeability coefficient** (***P***) of the two primary study drugs, rifampicin (RIF) and kanamycin (KM) in large necrotic nodules.

Permeability coefficient (***P***): the speed at which a compound moves through a tissue, generally due to diffusion but also to facilitated transport. Its units are cm/sec. In this study, the parameter to be determined for the rate of penetration of a drug to the lesions will be a combination permeability-surface area (**PS**) coefficient. This combined parameter is a flow, its units are cm^2.cm/sec or volume/time, but it can also be thought of as a diffusion-length term (cm^2s^{-1}.cm).

Relative permeability coefficient: the ratio of the permeability coefficient for diseased tissue over that for healthy tissue. Due to the limited tissue samples that will be available for estimating permeability, we will pool the tissue data for all subjects within a single drug group to determine the relative permeability of drug to diseased nodules versus healthy lung tissue. This will allow us to work with all the tissue samples for a given subject group to simultaneously model the permeability data. By assessing its relative value to healthy tissue, it decreases the dimensionality of the model and thus will help to obtain better estimates with limited data.

Diffusion rate constant: the constant which links the change of drug concentration over time to the concentration gradient according to the following equation:

$$dC_{lesion}(t)/dt = k_{12} \, . \, [C_{plasma}(t) - C_{lesion}(t)] - k_{21} \, . \, [C_{lesion}(t) - C_{plasma}(t)]$$

k_{12} : diffusion rate constant from blood (compartment 1) to lesion (compartment 2);
k_{21}: diffusion rate constant from lesion to blood.
The diffusion rate constant is a function of the permeability coefficient:

$$k_{12} = PS/V_1 \qquad k_{21}=PS/V_2$$

where V_1 and V_2 are the volumes of the two interconnected compartments (to be estimated from the drug concentration profiles in plasma and lesions).

Penetration ratio: the ratio between the area under the concentration-time curve in lesion and in blood or $\mathbf{AUC_{lesion} : AUC_{plasma}}$ [51], where AUC is Area Under the Curve.

5.2 Sample Size Justification

Comparison of the permeability was chosen as the primary endpoint rather than drug exposure in lesions relative to plasma. This type of endpoint is often adopted in case of sparse PK sampling design, where each subject essentially contributes plasma and tissue PK measurements at one or few time points. In such case, AUC or drug exposure over time cannot be estimated accurately but is rather fit to a model. On the other hand, since each time point/subject will contribute plasma, lung tissue and 2-5 lesion measurements, an estimation of P for each drug, using pooled PK data across subjects, can be made with much better precision than an estimation of AUC. Because a tissue sample will be taken from each subject only at one time point, pharmacokinetic data pooled across subjects will be used to fit a population PK model. However, for tractability, the sample size is justified by the variability expected in the individual permeability coefficient.

For any given drug, the variability of the permeability coefficient (P) across subjects or between similar lesions within one subject is expected to be around 50% or typical PK variability. The range of variation of P between drugs and lesion types is more difficult to assess. According to Sauermann et al [12], the range of P within abscesses can be as wide as 6 orders of magnitude. However, the study deals with abscesses which are widely scattered throughout the body and therefore don't have a common regional blood flow to draw from (except cardiac output). Also, since Sauermann et al [12] estimate the spatial parameters V and S and fix them in the data analysis, all the variability has to be incorporated into their estimate of P. In this study, we will have tissues from one anatomical location (lung) but of differing pathology.

The sample size for this study was determined based on the number of subjects necessary to detect a 50% difference in the individual permeability coefficient for caseous necrotic nodules between RIF and KM with 80% power. The SD of each P coefficient is assumed to be 50% the underlying population mean of P. Furthermore for the purposes of the sample size calculation, it is assumed that there is zero correlation between the 2 permeability coefficients. This assumption will be conservative so long as the coefficients for RIF and KM are not negatively correlated. With these assumptions, and normally distributed differences in the coefficient P between the 2 drugs, a paired t-test would need 12 subjects. A 20% inflation factor was applied to the sample size to allow for loss of data due to some administrative or analytical issue. The required sample size for this study is thus 15 subjects.

5.3 Outcome Measures

5.3.1 Definitions of Terms

- **Caseous necrotic nodule**: closed lesion generally encapsulated with fibrotic tissue, and containing a caseous necrotic center surrounded with multiple cellular layers
- **Cavity**: thick-walled open lesion communicating with an airway and often containing liquefied necrotic material
- **Permeability coefficient (P)**: the speed at which a compound moves through a tissue, generally due to diffusion but also to facilitated transport. Its units are cm/sec. In this study, the parameter to be determined for the rate of penetration of a drug to the lesions will be a combination permeability-surface area (PS) coefficient.
- **Penetration ratio**: the ratio between the area under the concentration-time curve in lesion and in blood or AUC_{lesion} : AUC_{plasma} [51].

5.3.2 Primary Endpoint

The comparison between the relative permeability coefficients of RIF and KM in pathologically defined large caseous necrotic nodules. The relative permeability coefficient is estimated from the pooled PK data as the ratio of the permeability in diseased tissue over that in healthy tissue.

5.3.3 Secondary Endpoints

1) The comparison between the absolute permeability coefficients of each of the five drugs in uninvolved lung and in closed necrotic lesions

2) The comparison between the relative permeability coefficients of RIF, KM, INH, PZA and MXF in caseous necrotic nodules versus open cavities

3) The comparison between the exposure ratio or ratio between the Area Under the Curve (AUC_{0-24}) in lesions (AUC_{lesion}), uninvolved lung (AUC_{lung}) and plasma (AUC_{plasma}) for RIF and KM in large caseous necrotic nodules
4) The comparison between the exposure ratio or ratio between the Area Under the Curve (AUC_{0-24}) in plasma (AUC_{plasma}), uninvolved lung (AUC_{lung}) and lesions (AUC_{lesion}) for each drug in each lesion type
5) Standard PK/PD indices for each drug in each lesion type (AUC_{0-24}/MIC; AUC_{0-24}/MBC; AUC_{0-24}/MPC; Cavg/MBC)
6) The correlation between permeability coefficients of the 5 study drugs and lesion type, determined by histopathology and HRCT
7) The correlation between permeability coefficients of the 5 study drugs and their respective physico-chemical properties
8) The correlation between macroscopic observations from High-Resolution CT and Dynamic MRI with lesion types as determined by microscopic histopathological observations
9) The concentration gradients of the five study drugs within the different layers of closed lesions, as determined by mass spectrometry imaging across tissue sections
10) The distribution and function of the vascular network present around individual lesions, determined by analyzing dynamic MRI values

5.4 Statistical Methods for Data Analysis

5.4.1 Primary Analysis

Descriptive statistics will be calculated to assess subject characteristics as a whole, as well as by the drug-delivery group assignment. Statistical summaries will also be calculated for the measured drug physico-chemical properties and in vitro PK parameters. These statistics include: for categorical variables, the number and percent in each category; for continuous variables, the mean, median, standard deviation, and range (minimum, maximum).

The primary analysis of the primary endpoint will use the parameter and variance estimates from the population PK model for the relative permeability (P) of disease tissue (large necrotic nodules) compared to healthy tissue for KM and RIF. From the model estimates, a 95% confidence interval for the difference in the relative P for RIF and KM will be calculated. If 0 lies outside the 95% confidence interval, the difference in permeability for the 2 drugs is declared significant.

An analysis of the primary endpoint will take place once the final subject has completed his/her surgery and all samples have been analyzed.

5.4.2 Secondary Analysis

A brief description of some of the planned analysis for secondary endpoints is given below. In a manner similar to the primary analysis, pair wise comparisons of relative permeability between other pairs of drugs for a fixed tissue type, as well as the relative permeability comparison for caseous necrotic nodules versus open cavities for each drug, will be examined by calculating the 95% confidence intervals for the difference estimated from the model parameters and variance

for each comparison of interest. Confidence intervals for examining the difference in exposure ratios between the AUC in plasma, uninvolved lung, and lesions for each drug and lesion type will be generated using Monte Carlo simulation and the model estimates for the relevant parameter and their variances. A similar analysis will also be done for the other standard PK/PD indices of interest.

The correlation between the permeability coefficients and physico-chemical properties for the 5 study drugs will be examined graphically by plotting the values of the permeability against different measured physico-chemical properties for each of the study drugs and lesion types.

6 Adverse Event Reporting Plan

6.1 Definition of an Adverse Event

Adverse Events (AEs) are any unfavorable and unintended diagnosis, symptom, sign (including an abnormal laboratory finding), syndrome or disease which either occurs during the study, having been absent at baseline, or, if present at baseline, appears to worsen. Stable chronic conditions which are present prior to clinical trial entry and do not worsen are not considered AEs and will be accounted for in the subject's medical history**.**

Serious Adverse Events (SAEs) are any untoward medical occurrences that: (1) result in death, (2) are life-threatening, (3) require (or prolong) hospitalization, (4) cause persistent or significant disability/incapacity, (5) result in congenital anomalies or birth defects, or (6) are other conditions which in the judgment of the investigators represent significant hazards.

Expected Adverse Events are AEs that are listed as possibly related to the study agent/intervention in the protocol, informed consent document, or the package insert.

Unanticipated problem that is not an Adverse Event (UPnonAE): An unanticipated problem that does not fit the definition of an adverse event, but which may, in the opinion of the investigator, involves risk to the subject, affect others in the research study, or significantly impact the integrity of research data. For example, report occurrences of breaches of confidentiality, accidental destruction of study records, or unaccounted-for study drug.

Protocol Violation: Any change, divergence, or departure from the study procedures in an IRB-approved research protocol that has a major impact on the subject's rights, safety, or well-being and/or the completeness, accuracy or reliability of the study data.

Protocol Deviation: Any change, divergence, or departure from the study design or procedures of a research protocol that is under the investigator's control and that has not been approved by the IRB. It does not have a major impact on the subject's rights, safety or well-being, or the completeness, accuracy and reliability of the study data.

Unanticipated Problem (UP) is any incident, experience, or outcome that is:

1. unexpected in terms of nature, severity, or frequency in relation to
   a. the research risks that are described in the IRB-approved research protocol and informed consent document; Investigator's Brochure or other study documents; and
   b. the characteristics of the subject population being studied; and

2. related or possibly related to participation in the research; and
3. places subjects or others at a greater risk of harm (including physical, psychological, economic, or social harm) than was previously known or recognized.

6.2 Grading Adverse Events for Severity

The severity of each AE will be determined using the modified DAIDS Table for Grading Adult Adverse Experiences. This table can be found using the following link: DAIDS 2004 Toxicity Table. Any events that are not listed in this toxicity table will be graded by the local PI as follows:

Grade 1 - Mild	Transient or mild discomfort; no limitation in activity; no medical intervention/ therapy required
Grade 2 - Moderate	Moderate limitation in activity – some assistance may be needed; no or minimal medical intervention/ therapy required
Grade 3 - Severe	Marked limitation in activity; some assistance usually required; medical intervention/therapy required, hospitalizations possible
Grade 4 - Life-threatening	Extreme limitation in activity, significant assistance required; significant medical intervention/therapy required, hospitalization or hospice care probable
Grade 5 - Death	

6.3 Assessing Adverse Events for Relationship to Study

Any AE that occurs in a subject will be assessed for its relationship to the study. A causal relationship means a drug caused (or is reasonably likely to have caused) the AE. This usually implies a relationship in time between one or more drugs/study agents and the AE—for example, the AE occurred shortly after the subject received the drugs/study agents.

For all AEs, the clinician who examines and evaluates the subject will determine the AE's causality based upon the temporal relationship to administration of the study agents, the pharmacology of the study agents, and his/her clinical judgment.

The following scale will be used to reflect the PI's judgment as to the relationship between the study agents and the AE:

Definitely Related: The AE is clearly related to one or more of the study agents – follows a reasonable temporal sequence from administration of one or more of the study agents, follows a known or expected response pattern to the one or more of the study agents that is confirmed by

improvement on stopping and reappearance of the event in repeated exposure and that could not be reasonably explained by the known characteristics of the subject's clinical state.

***Probably Related**:* The AE and administration of the study agents are reasonably related in time and/or follows a known pattern of response, and the AE is more likely explained by one or more of the study agents than other causes.

Possibly Related: AE follows a reasonable temporal sequence from administration of the study drugs, follows a known or expected response pattern to the suspected drug or drugs, but that could readily have been produced by a number of other factors.

***Unlikely Related**:* A potential relationship between one or more of the study agents and the AE could exist (i.e., the possibility cannot be excluded), but the AE is most likely explained by causes other than one or more of the study agents (e.g., could readily have been produced by the subject's clinical state or could have been due to environmental or other interventions)

Unrelated: AE is clearly not related to one or more of the study agents – another cause of the event is most plausible and/or a clinically plausible, temporal sequence is consistent with the onset of the event and the study medication administration and/or event is biologically implausible.

6.4 Adverse Event Documentation

Any AE that will be reported on this trial will be fully and completely documented on the Adverse Event Case Report Form (CRF) and in the subject/study participant's clinical chart. The start date, the stop date, the severity of each reportable event, and the Investigator's judgment of the AEs relationship to the study agent/intervention will also be recorded on the CRF. In the event that the subject is withdrawn from the study due to an AE, it must be recorded on the CRF as such.

6.5 Adverse Event Treatment

Once an AE is known, staff at the study site should ensure that the subject receives prompt and appropriate care. Should a subject call a study clinician to report an AE, it will be determined at that time if an extra visit(s) will be scheduled, and/or appropriate medical advice will be provided. All actions taken by the investigator after observing an AE should be documented, including increased monitoring of the subject/study participant, suspension of the treatment, etc. Additionally, all calls will be documented in the subject/study participant's study chart, and discussed with the PI.

6.6 Potential Hazards

6.6.1 Anticipated Adverse Events

Each study drug will be administered only once, unless it is part of the subject's drug regimen prior to study initiation. Anticipated AEs related to a single dose of drug are as follows:

RIF: Orange staining of body fluids and GI upset.
INH: GI upset.
PZA: GI upset.

KM: Nephrotoxicity, vestibular toxicity ototoxicity (mostly with advanced age and prolonged use but possible with single dose) and local pain with IM injections,
MXF: Occasional GI intolerance, headache, insomnia, restlessness, dizziness and remote possibility of QT prolongation.

AEs and contraindications for all 5 study drugs can be found in "Drug-Resistant Tuberculosis: A Survival Guide for Clinicians." [52]

Subjects will be monitored by the primary care team for these AEs. In women, the appearance of vaginal discharge will prompt a diagnostic work-up, or subjects will be empirically treated for Candida vaginitis.

7.7 Study Sites AE Reporting

Since the subjects on this protocol will be seen because of illness, and are likely to be ill at baseline, many will have AEs by the above criteria. However, their medical management will be standard of care for those illnesses and complications and not part of the research process. Therefore, AEs associated with the underlying disease will not be recorded as AEs. However, AEs assessed to be related to the research will be recorded as such. As described below, all SAEs will be recorded and reported.

Any grade 3 or 4 AE (as defined in Section 7.1) that is possibly, probably or definitely related to study drugs and occurs between the times a subject signs the informed consent form and the time s/he completes the planned surgery (or at the time of early discontinuation of the subject from study participation for any reason) will be captured and recorded and reported with continuing annual review.

7.8 Study Sites SAE Reporting Requirements

All deaths and life-threatening SAEs reported as above will be reported to the main PI on the study within 3 business days. SAEs that are possibly, probably, or definitely related to the research will be reported to the NIAID IRB within 7 days of investigators awareness, regardless of expectedness. The main PI (NIAID) will report SAEs and AEs reported during this study to all sites at the time of continuing review.

In Korea, local PIs have a responsibility to report AEs to each IRB and if needed, to MFDS.

7.9 Adverse Event Reporting to the NIAID, Asan, National Medical Center, and Pusan Institutional Review Boards and RCHSPB Safety

As stated above, all sites will report directly to the main PI at the NIAID. Each site must also report to their respective IRBs. The NIAID site must in addition report to RCHSPB Safety. Details of reporting are described in the sections below. Respective reporting will be made by telephone, fax or email to the appropriate addresses.

7.9.1 PI reporting to the NIAID IRB

7.9.1.1 Expedited Reporting to the NIAID IRB

Unanticipated problems that are either AEs or non-AEs (as defined above) and Protocol Violations will be reported within 7 calendar days of investigator awareness. SAEs that are possibly, probably, or definitely related to the research will be reported to the NIAID IRB within 7 days of investigators awareness, regardless of expectedness.

All deaths will be reported within 7 calendar days of investigator awareness.

7.9.1.2 Annual Reporting to the NIAID IRB

The following items will be reported to the NIAID IRB in summary at the time of Continuing Review:

- All unanticipated problems, including AEs and non-AEs
- Expected serious adverse events that are possibly, probably, or definitely related to the research
- Serious adverse events that are not related to the research
- All protocol deviations which in the opinion of the investigator should be reported.
- Any trends or events which in the opinion of the investigator should be reported

(Protocol violations and deviations mentioned above are defined in the monitoring entity policy.)

7.9.2 PI reporting to RCHSPB Safety

1) The NIAID PI is responsible for reporting to RCHSPB Safety.
2) NIAID Investigators must report to RCHSPB Safety within 7 days after becoming aware of a subject death or a potentially life-threatening serious adverse event.
3) NIAID Investigators must report to RCHSPB Safety within 15 days after becoming aware of an inpatient hospitalization (other than elective) or a persistent or significant disability/incapacity which fits the reporting requirements.
4) NIAID Investigators will report within 15 days any other event or condition regardless of grade, which in their judgment represents an event reportable to RCHSPB Safety.

7.9.3 Asan Site Reporting to the Asan IRB

In addition to reporting to the NIAID PI (as above), Asan investigators should report all reportable SAEs and AEs to the Asan IRB and the director as promptly as possible within the time period described as follows:

1) For the cases at Asan, ALL SAEs at occurring at Asan should be reported to the Asan IRB within one business day of the investigator's awareness. .
2) Serious and unexpected Adverse Drug Reactions at other sites that lead to death or are life-threatening must be reported to the AMC IRB within 7 business days of AMC's awareness. Other ADRs from other sites should be reported to the AMC IRB within 15 days of AMC's awareness.

The sponsor must provide continuing report until the AE comes to an end (loss of this adverse reaction or when it is impossible to do the follow up investigation).

7.9.4 PNUH Site Reporting to the PNUH IRB

In addition to reporting to the NIAID PI (as above):

1) PNUH Investigators must provide a report to the PNUH IRB within 24 hours after becoming aware of a subject death or a potentially life-threatening serious adverse event at occurring at PNUH. Any other SAEs at occurring at PNUH should be reported to the PNUH IRB within 7 days of the investigator's awareness.
2) Any other SAEs occurring at other sites must be reported to PNUH IRB within 15 days after the PNUH investigator's awareness.
3) Protocol violations which the investigator decides are important will be reported to the PNUH IRB within 7 days of the PNUH investigator's awareness. Other protocol violations will be reported to the PNUH IRB quarterly.

7.9.5 NMC Site Reporting to the NMC IRB

In addition to reporting to the NIAID PI (as above) local investigators should quickly report all serious and unexpected adverse events related to study design, study intervention, and/or study agent/device within the following timeframe:

1) Local investigators must provide a preliminary report to the NMC IRB within 7 days after becoming aware of a subject death or a potentially life-threatening adverse event. A detailed report should follow within 8 days after the initial report. If a death is reported, the additional information, such as the autopsy report (if performed) and death certificate, should be provided at this time. In addition, these items will be reported to NMC IRB at the time of Continuing Review.
2) Local investigators must provide a report to the local IRB within 15 days after becoming aware of any other unexpected adverse events which are serious in nature**.**
3) Investigator must provide follow up report until the AE comes to an end (resolved or impossible to follow up).

Reporting requirements of an unanticipated problem (UP) which harms the subject(s) and/or others:

1) Local Investigator must report the following items to the IRB within 7 days of awareness.
   a) New information which negatively affects the safety of the subject(s) or the conduct of clinical trial
   b) Changes which have a significant impact on the conduct of clinical trial or places subject(s) at a greater risk of harm.
2) Investigator should describe any corrective action initiated during the study in order to protect the subject(s) from potential or further damage and to prevent a recurrence of the problem.
3) Other UPs will be reported to the NMC IRB in summary at the time of Continuing Review. If not applicable, it is possible to describe simply in summary as 'there was no UP'.

In the case of a multicenter trial, if the protocol or informed consent form needs to be changed due to UPs from other sites which increase the risk for the subjects, the UP(s) should be reported to NMCIRB.

8 Data Management Plan---Data Collection, Sample Storage and Publication

8.1 Data Collection

Study data will be collected on standardized paper CRFs prepared in conjunction with the RCHSPB of NIAID/NIH and the NIAID study team. The local study team will use only NIAID-approved CRFs. The PI is responsible for assuring that the data collected is complete, accurate, and recorded in a timely manner. The CRFs will be collected and placed into a subject-specific binder. Source documentation (the point of initial recording of a piece of data) should support the data collected on the case report form, and be signed and dated by the person recording and/or reviewing the data. Source documents include all recordings of observations or notations of clinical activities and all reports and records necessary for the evaluation and reconstruction of the clinical trial. Source documents include, but are not limited to, the subject medical records, electronic chart records, laboratory reports, electrocardiogram (EKG) tracings, x-rays, radiologist reports, biopsy reports, ultrasound photographs, subject progress notes, pharmacy records and any other similar reports or records of procedures performed during the subject's participation in the protocol. Data for CRFs will be collected during subject visits by health care providers and abstracted from the medical record. Once the data is collected, it will be reviewed by the RCHSPB monitoring team or their contractors. Once reviewed, a copy of each CRF will remain at the site while the original will be retained by the monitoring team and a copy sent to the NIAID investigators. Any data that is compiled for statistic or other manipulation will be handled in an Access database. The scientific results from this study will require various formats, depending on the data type. Locked copies of these files containing the results will be compiled by the research supervisor and made available to monitoring and regulatory agencies as necessary.

HRCT and Dynamic MRI image files will be transferred to the ITRC team and will then be transferred to NIAID. The radiology analysis team at NIAID will access these stored records to perform their analysis. The results of the analysis will included numerical and image output that will be deposited into the TBRS database.
Case Report Forms (CRFs) will be filled out by the health care providers. Information for the CRFs will be obtained from the subject interview upon consent, medical records, and data accrued in the trial, and will include a medical history, lab results and specimen data, and scan results obtained in the trial.

8.2 Data Management

Data entered onto the CRF will be entered into an access database, which will be managed and monitored by the ITRC-NIH team. Access to the database is password controlled and will be limited to those with data entry and management responsibilities. Records are protected by ownership control and a complete log of all activities within the system is recorded. All study related data will be maintained on servers located at ITRC and NIH and image files will be stored and indexed in the system.

8.3 Data Storage

All essential documentation for all study subjects including history and physical findings, laboratory data, and results of consultations are to be maintained by the investigators in a secure storage facility for a minimum of three years. These records are to be maintained in compliance

with IRB/EC (Institutional Review Board/Ethics Committee), local and government requirements, whichever is longest. All records are to be kept confidential to the extent provided by law.

8.4 Publication of Research Findings

Collaborating protocol team members will own the data generated by or resulting from this project, and they may arrange for publication of this original research (with consent of all study investigators) in a primary scientific journal, and for copyright by the journal unless the journal's copyright policy would preclude individuals from making or having made a single copy of any such article for their own use.

8.5 Sample Collection

Sputum, urine and blood samples dedicated for drug quantification will be processed at the research laboratories of ITRC, Yonsei University College of Medicine or each hospital where this protocol is taking place. Bacteriological testing of sputum samples will also be conducted using standard procedures in either the ITRC or BSL-3 Yonsei laboratories. Dissection of resected lung tissue will be conducted in either the ITRC BSL-3 laboratory or the allotted space in the Hospital Clinical microbiology or pathology laboratories as designated by each site. All data collected at these sites will be entered on CRFs.

Quantification of drug levels in plasma, sputum, urine, lung and lesion samples will be conducted at UMDNJ laboratories in New Jersey. Gamma-irradiation of lung and lesion samples prior to imaging MS analysis and corresponding histological analysis and sample preparation will be conducted at NIAID in Bethesda, MD and the Jain Lab at Massachusetts General Hospital. Imaging MS analyses will be carried out at The Novartis Institute for Biomedical Research (NIBR) in Basel (Switzerland) in the Department of Discovery Technology. Analysis of pharmacokinetic data using modeling tools and software will be carried out at NIBR in the Modeling & Simulation Department. Determination of MTB genome equivalents and profiling of bacterial RNA expression will be conducted at NIAID in Bethesda, MD.

8.6 Sample Storage

Sputum, blood, plasma, urine, lung and lesion specimens collected at AMC, NMC and PNUH will be transported to, processed and stored in the ITRC building pathology lab. All specimens will be labeled, indexed and stored at ITRC by study ID so that review of the actual specimens is possible by all investigators on the protocol. Non-infectious specimens will be stored in the ITRC specimen bank freezer room in below -70 degrees C freezers. Infectious specimens will be stored inside the ITRC BSL-3 laboratory. All sets of specimens will be stored in locked rooms, access to the specimens will be controlled and use will be recorded in study specific log books.

Samples to be analyzed by investigators outside of ITRC, Yonsei University, AMC, NMC and PNUH will be shipped by an experienced courier (e.g., World Courier). Sputum, plasma, urine, lung and lesion samples for pharmacokinetic analyses will be shipped to UMDNJ laboratories. Lung and lesion sample portions dedicated for imaging MS and histological analysis will be first shipped to NIAID for □-irradiation and then to NIBR for analysis. Sputum or bacterial isolates and lesion sample portions dedicated to bacteriological genome analysis and RNA profiling will be shipped to NIAID. Treated sputum and bacterial isolates will be shipped to NIAID. NIAID

and UMDNJ may transfer samples to contractors or collaborators for analysis not currently conducted within their respective laboratories.

Should sharing of specimens with institutions not on the protocol be desired, the NIAID and hospital IRBs will review and approve a protocol amendment specifying the conditions under which the human specimens may be shared, ensuring adequate provisions to protect the privacy of subjects. Clinical information shared about the samples would similarly require prior IRB approval. The research use of stored, unlinked or unidentified samples may be exempt from the need for prospective IRB review and approval. Exemption requests will be submitted in writing to the NIH Office of Human Subjects Research, which is authorized to determine whether a research activity is exempt. Cultured bacterial isolates may be transferred without additional IRB review only as anonymous strains with limited data including no personal identifiers with the agreement of the PI.

Additionally, subjects may decide at any point not to have their samples stored. In this case, the principal investigator will destroy all known remaining samples and report what was done to both the subject and to the IRB. This decision may not affect the subject's participation in this protocol or any other protocols at NIH.

A 20% loss of or destruction of samples will constitute a compromise of the scientific integrity of the data collected and will be reported to all IRBs. At the termination of the NIAID study, any remaining samples will be retained or disposed of as determined by the study sites and their respective IRBs, according to Korean laws.

9 Protocol Monitoring Plan

The RCHSPB of NIAID/NIH will monitor this trial for source document verification and regulatory compliance. The RCHSPB monitoring team will discuss a detailed monitoring plan with the PI.

The study will be conducted in compliance with this protocol, International Conference on Harmonization of Good Clinical Practices (ICH GCP) and all applicable regulatory requirements. Monitors under contract to the NIAID will visit designated clinical research sites to monitor all aspects of the study in accordance with the appropriate regulations. The objectives of a monitoring visit will be:

1) To verify the prompt reporting of all data points, including Adverse Events requiring expedited reporting, to check the availability of sign informed consent
2) To compare individual subjects' records (case report forms, data pulls) to the source documents (supporting data, laboratory specimen records, medical records to include physician progress notes, nurses notes, subjects' hospital charts)
3) To ensure protection of study subjects, compliance with the protocol, and accuracy and completeness of records

The monitors will also inspect the clinical site's regulatory files to ensure that applicable regulatory requirements are being followed. During the monitoring visits the Investigator, and/or designee, and other study personnel will be available to discuss the study. The Investigator (and/or designee) will make study documents (e.g., consent forms, CRFs) and pertinent hospital

or clinical records readily available for inspection by the local IRB, the FDA, the MFDS, the site monitors, and the NIAID staff for confirmation of the study data.

Any changes or additions to the protocol will be submitted to all necessary IRBs and relevant regulatory agencies for review. The written IRB approvals will be filed in the investigator's study binder, and a copy of the approvals will be forwarded to the monitoring team. Furthermore, essential documents will be collected in the study binders and will include:

1) IRB/EC approvals for the study protocol and all amendments
2) All source documents and laboratory records
3) CRF copies
4) Informed consent forms

10 Subject Protection

This protocol must receive the approval of the NIAID, Asan, NMC, and PNUH Institutional Review Boards prior to implementation. Each site may start implementing the protocol separately as long as that respective site and NIAID have approval. The study will be conducted in accordance with the design and specific provisions of this IRB approved protocol, in accordance with the ethical principles that have their origin in the Declaration of Helsinki, and that are consistent with Good Clinical Practice (GCP) and any applicable regulatory requirement(s), as well as in accordance with the NIAID policies. The PI will assure that no deviation from, or changes to the protocol will take place without prior agreement and documented approval from the IRB, except where necessary to eliminate an immediate hazard(s) to the trial participants. The PI will promptly report to the IRBs and to NIAID any changes in research activity and will promptly report to the IRBs all unanticipated problems involving risk to human subjects, or others.

10.1 Rationale for Subject Selection

Asan, NMC, and PNUH were selected due to the high rate of surgeries for MTB infection in the population seen at these hospitals. Korea is an interesting place to study TB for several reasons: (1) Korea has a high rate of tuberculosis infection, with ~70 cases of active tuberculosis per 100,000 population; (2) Korea has a high rate of infection with drug-resistant strains of *M. tuberculosis* (10.6% and 2.2% prevalence of any drug-resistance and multi-drug resistance, respectively, among new and previously-treated cases of tuberculosis), as well as patients resistant to almost all known TB therapies; (3) rates of HIV infection in the Korean population are low, reducing the likelihood that host immunosuppression contributes to treatment failure or relapse; (4) existence of a well-managed, specialized hospitals with highly-trained staff. Also, surgery is a known predictor of favorable outcome in Korean patients with MDR-TB (Shim and Kwon, unpublished).

10.2 Participation of Children and Other Vulnerable Subjects

This study will enroll only persons from 20 years and above. It is rare for subjects less than 20 years old to undergo lung resection at each of the three study centers, so few subjects will be functionally excluded on this basis. In addition, if a child at one of these hospitals did need resection surgery, he or she would be transferred to a children's hospital for the operation.

10.3 Risks/Benefits Analysis including Considerations of Alternatives to Participation

10.3.1 Potential Benefits to Study Subjects

There will be no benefit to subjects for participating in this study.

10.3.2 Potential Risks to Study Subjects

1) Each study drug will be administered only once, unless it is part of the subject's drug regimen prior to study initiation. Among the following side effects, those associated with cumulative toxicity upon prolonged administration are not anticipated as a consequence of the present study. **All risks except ones in bold are extremely unlikely to occur with a single dose.**

2) **RIF**: **Orange staining of body fluids**, **GI upset,** rash and pruritus, flu-like syndrome (usually only with intermittent administration), hepatotoxicity, hematologic abnormalities (thrombocytopenia, hemolytic anemia).

   INH: Peripheral neuropathy, hypersensitivity reactions, optic neuritis, arthralgias, CNS changes, drug-induced lupus, and diarrhea and cramping with liquid product

   PZA: Gout (hyperuricemia) and arthralgias, hepatotoxicity, rash, photosensitivity, **gastrointestinal upset**.

   KM: Nephrotoxicity and ototoxicity (mostly with advanced age and prolonged use), local pain with IM injections, vestibular toxicity, electrolyte abnormalities, including hypokalemia and hypomagnesemia.

   MXF: **Occasional GI intolerance, headache, malaise, insomnia, restlessness**, dizziness, allergic reactions, diarrhea including C. difficile, photosensitivity, increased hepatic enzymes, occasional tendon rupture as well as QT prolongation.

10.3.3 Risk-Benefit Analysis

Subjects enrolled in this study will have elected lung resection surgery likely because they have failed first and second line therapy and face a substantial risk of death. As part of this study, 5 approved TB drugs will be administered only once prior to surgery, which minimizes the risk of adverse events. The major objective of this study is to understand how well the 5 study drugs are permeating TB lesions. The benefit to the subjects could be a potentially more effective management of the TB disease with potentially more appropriate drug doses; however there may be no benefit to subjects. Subjects that elect not to participate will still be undergoing their elective surgery, thus there is no penalty for not participating. Overall the potential benefits of participating in the study outweigh the very small risks of the interventions that will be performed.

10.4 Privacy and Confidentiality

All laboratory specimens, evaluation forms, reports, and other records that leave the site will be identified by coded number only to maintain subject confidentiality. All records will be kept locked. All computer entry and networking programs will be done with coded numbers only. Clinical information will not be released without written permission of the subject, except as necessary for monitoring by the IRB, NIAID, or Office for Human Research Protections (OHRP).

10.5 Remuneration

Subjects will be given 325,000 won for their participation in the study. If the study is paying for otherwise unpaid out-of-pocket expenses this payment to the subject will be considered remuneration and the 325,000 won will not be given to the subject.

11 Pharmaceutical, Biologic, and Device Info

Study agent on the market will be purchased by ITRC, labeled based on applicable regulations, and delivered to the pharmacies of each study site. The drugs will be managed by designated pharmacists and the stocking, storage, dispensation, and return of study drugs will be recorded in a study document.

11.1 Formulation and Preparation of study drugs

RIF, INH, PZA and MXF will be administered as tablets. For KM, one gram (750mg if subject is <50kg of bodyweight) will be dissolved in 5mL of normal saline (0.9% NaCl) and injected intramuscularly into the gluteal muscle.

11.2 Stability and Storage of Study Drugs

Drugs will be stored per their respective package insert.

11.3 Incompatibilities of Study Drugs

- **RIF**: Rifampicin induces hepatic enzymes, and may increase the dosage requirements of drugs metabolized in the liver. These include corticosteroids, steroid contraceptives, oral hypoglycaemic agents, oral anticoagulants, phenytoin, cimetidine, cyclosporin and digitalis glycosides.

- **INH**: Isoniazid tends to raise plasma concentrations of phenytoin and carbamazepine by
- inhibiting their metabolism in the liver. The absorption of isoniazid is impaired by aluminium hydroxide.

- **PZA:** No reported drug incompatibilities.

- **KM:** Other ototoxic or nephrotoxic drugs should not be administered to subjects receiving KM. These include other aminoglycoside antibiotics, amphotericin B, cefalosporins, etacrynic acid, cyclosporin, cisplatin, furosemide and vancomycin. Streptomycin may potentiate the effect of neuromuscular blocking agents administered during anesthesia.

- **MFX**: drugs which exacerbate QT prolongation such as Solatol, a general antiarrhythmic, should be avoided.

These incompatibilities were obtained from:
Francis J. Curry National Tuberculosis Center and California Department of Health Services, 2004: Drug-Resistant Tuberculosis: A Survival Guide for Clinicians,

11.4 Administration Procedures

The study pharmacist at each hospital will be required to maintain complete records of the study drugs dispensed and administered.

12 References

1. Assandri, A., B. Ratti, and T. Cristina, *Pharmacokinetics of rifapentine, a new long lasting rifamycin, in the rat, the mouse and the rabbit.* J Antibiot (Tokyo), 1984. **37**(9): p. 1066-75.
2. Della Bruna, C., et al., *LM 427, a new spiropiperidylrifamycin: in vitro and in vivo studies.* J Antibiot (Tokyo), 1983. **36**(11): p. 1502-6.
3. Murdoch, M.B. and L.R. Peterson, *Antimicrobial penetration into polymorphonuclear leukocytes and alveolar macrophages.* Semin Respir Infect, 1991. **6**(2): p. 112-21.
4. Del Tacca, M., et al., *Penetration of clofoctol into human lung.* J Antimicrob Chemother, 1987. **19**(5): p. 679-83.
5. Watanabe, A., et al., *Penetration of minocycline hydrochloride into lung tissue and sputum.* Chemotherapy, 2001. **47**(1): p. 1-9.
6. Naline, E., et al., *Penetration of minocycline into lung tissues.* Br J Clin Pharmacol, 1991. **32**(3): p. 402-4.
7. Danesi, R., et al., *Comparative distribution of azithromycin in lung tissue of patients given oral daily doses of 500 and 1000 mg.* J Antimicrob Chemother, 2003. **51**(4): p. 939-45.
8. Seno, N., S. Matsumoto, and H. Yamamoto, *[Clinical study on the penetration of latamoxef into the pulmonary tissue in surgery of the chest].* Jpn J Antibiot, 1985. **38**(12): p. 3471-6.
9. Stucki, A., et al., *Efficacy of telavancin against penicillin-resistant pneumococci and Staphylococcus aureus in a rabbit meningitis model and determination of kinetic parameters.* Antimicrob Agents Chemother, 2006. **50**(2): p. 770-3.
10. Carryn, S., et al., *Comparative intracellular (THP-1 macrophage) and extracellular activities of beta-lactams, azithromycin, gentamicin, and fluoroquinolones against Listeria monocytogenes at clinically relevant concentrations.* Antimicrob Agents Chemother, 2002. **46**(7): p. 2095-103.
11. Gladue, R.P., et al., *In vitro and in vivo uptake of azithromycin (CP-62,993) by phagocytic cells: possible mechanism of delivery and release at sites of infection.* Antimicrob Agents Chemother, 1989. **33**(3): p. 277-82.
12. Sauermann, R., et al., *Antibiotic abscess penetration: fosfomycin levels measured in pus and simulated concentration-time profiles.* Antimicrob Agents Chemother, 2005. **49**(11): p. 4448-54.
13. Wagner, C., R. Sauermann, and C. Joukhadar, *Principles of antibiotic penetration into abscess fluid.* Pharmacology, 2006. **78**(1): p. 1-10.
14. Bartlett, J.G., *Experimental aspects of intraabdominal abscess.* Am J Med, 1984. **76**(5A): p. 91-8.
15. Cremieux, A.C., et al., *Ceftriaxone diffusion into cardiac fibrin vegetation. Qualitative and quantitative evaluation by autoradiography.* Fundam Clin Pharmacol, 1991. **5**(1): p. 53-60.
16. Carbon, C., A.C. Cremieux, and B. Fantin, *Pharmacokinetic and pharmacodynamic aspects of therapy of experimental endocarditis.* Infect Dis Clin North Am., 1993. **7**(1): p. 37-51.
17. Drexler, D.M., et al., *Utility of imaging mass spectrometry (IMS) by matrix-assisted laser desorption ionization (MALDI) on an ion trap mass spectrometer in the analysis of drugs and metabolites in biological tissues.* J Pharmacol Toxicol Methods, 2007. **55**(3): p. 279-88.

18. Chaurand, P., D.S. Cornett, and R.M. Caprioli, *Molecular imaging of thin mammalian tissue sections by mass spectrometry.* Curr Opin Biotechnol, 2006. **17**(4): p. 431-6.
19. Zhang, Y., et al., *Role of acid pH and deficient efflux of pyrazinoic acid in unique susceptibility of Mycobacterium tuberculosis to pyrazinamide.* J Bacteriol, 1999. **181**(7): p. 2044-9.
20. Kaplan, G., et al., *Mycobacterium tuberculosis growth at the cavity surface: a microenvironment with failed immunity.* Infect Immun., 2003. **71**(12): p. 7099-108.
21. Lee, J.Y., et al., *Pulmonary tuberculosis: CT and pathologic correlation.* J Comput Assist Tomogr, 2000. **24**(5): p. 691-8.
22. Im, J.G., et al., *Pulmonary tuberculosis: CT findings--early active disease and sequential change with antituberculous therapy.* Radiology, 1993. **186**(3): p. 653-60.
23. Kono, R., et al., *Dynamic MRI of solitary pulmonary nodules: comparison of enhancement patterns of malignant and benign small peripheral lung lesions.* AJR Am J Roentgenol, 2007. **188**(1): p. 26-36.
24. Zou, Y., et al., *Quantitative investigation of solitary pulmonary nodules: dynamic contrast-enhanced MRI and histopathologic analysis.* AJR Am J Roentgenol, 2008. **191**(1): p. 252-9.
25. Dartois, C., et al., *Overview of model-building strategies in population PK/PD analyses: 2002-2004 literature survey.* Br J Clin Pharmacol, 2007. **64**(5): p. 603-12.
26. Pillai, G.C., F. Mentre, and J.L. Steimer, *Non-linear mixed effects modeling - from methodology and software development to driving implementation in drug development science.* J Pharmacokinet Pharmacodyn, 2005. **32**(2): p. 161-83.
27. Barclay, W.R., et al., *Distribution and excretion of radioactive isoniazid in tuberculous patients.* J Am Med Assoc, 1953. **151**(16): p. 1384-8.
28. Kislitsyna, N.A. and N.I. Kotova, *[Rifampicin and isoniazid concentration in the blood and resected lungs in tuberculosis with combined use of the preparations].* Probl Tuberk, 1980(8): p. 63-5.
29. Canetti, G., et al., *[Rifomycin levels in the lung and tuberculous lesions in man].* Acta Tuberc Pneumol Belg, 1969. **60**(3): p. 315-22.
30. Furesz, S., et al., *Rifampicin: a new rifamycin. 3. Absorption, distribution, and elimination in man.* Arzneimittelforschung, 1967. **17**(5): p. 534-7.
31. Kiss, I.J., et al., *Investigation on the serum and lung tissue level of rifampicin in man.* Int J Clin Pharmacol Biopharm, 1976. **13**(1): p. 42-7.
32. Hutschala, D., et al., *In vivo measurement of levofloxacin penetration into lung tissue after cardiac surgery.* Antimicrob Agents Chemother, 2005. **49**(12): p. 5107-11.
33. Di Paolo, A., et al., *Pharmacokinetics of azithromycin in lung tissue, bronchial washing, and plasma in patients given multiple oral doses of 500 and 1000 mg daily.* Pharmacol Res, 2002. **46**(6): p. 545-50.
34. Kislitsyna, N.A., *[Comparative evaluation of rifampicin and isoniazid penetration into the pathological foci of the lungs in tuberculosis patients].* Probl Tuberk, 1985(4): p. 55-7.
35. Saralaya, D., et al., *Serum and sputum concentrations following the oral administration of linezolid in adult patients with cystic fibrosis.* J Antimicrob Chemother, 2004. **53**(2): p. 325-8.
36. Grosset, J., et al., *Antagonism between isoniazid and the combination pyrazinamide-rifampin against tuberculosis infection in mice.* Antimicrob Agents Chemother., 1992. **36**(3): p. 548-51.

37. McIlleron, H., et al., *Determinants of rifampin, isoniazid, pyrazinamide, and ethambutol pharmacokinetics in a cohort of tuberculosis patients.* Antimicrob Agents Chemother, 2006. **50**(4): p. 1170-7.
38. Boman, G., *Serum concentration and half-life of rifampicin after simultaneous oral administration of aminosalicylic acid or isoniazid.* Eur J Clin Pharmacol., 1974. **7**(3): p. 217-25.
39. Acocella, G., et al., *Kinetics of rifampicin and isoniazid administered alone and in combination to normal subjects and patients with liver disease.* Gut., 1972. **13**(1): p. 47-53.
40. Diacon, A.H., et al., *Early bactericidal activity of high-dose rifampin in patients with pulmonary tuberculosis evidenced by positive sputum smears.* Antimicrob Agents Chemother, 2007. **51**(8): p. 2994-6.
41. Kubota, R., et al., *Dose-escalation study of isoniazid in healthy volunteers with the rapid acetylator genotype of arylamine N-acetyltransferase 2.* Eur J Clin Pharmacol, 2007. **63**(10): p. 927-33.
42. Ohno, M., et al., *Slow N-acetyltransferase 2 genotype affects the incidence of isoniazid and rifampicin-induced hepatotoxicity.* Int J Tuberc Lung Dis, 2000. **4**(3): p. 256-61.
43. Ellard, G.A., *Variations between individuals and populations in the acetylation of isoniazid and its significance for the treatment of pulmonary tuberculosis.* Clin Pharmacol Ther, 1976. **19**(5 Pt 2): p. 610-25.
44. Jutte, P.C., et al., *Penetration of isoniazid, rifampicin and pyrazinamide in tuberculous pleural effusion and psoas abscess.* Int J Tuberc Lung Dis, 2004. **8**(11): p. 1368-72.
45. Chan, K. and C.L. Wong, *Pharmacokinetics of pyrazinamide in plasma and CSF of rabbits following intravenous and oral administration.* Eur J Drug Metab Pharmacokinet, 1988. **13**(3): p. 195-9.
46. Ellard, G.A., et al., *Penetration of pyrazinamide into the cerebrospinal fluid in tuberculous meningitis.* Br Med J (Clin Res Ed), 1987. **294**(6567): p. 284-5.
47. Nagai, J. and M. Takano, *Molecular aspects of renal handling of aminoglycosides and strategies for preventing the nephrotoxicity.* Drug Metab Pharmacokinet, 2004. **19**(3): p. 159-70.
48. Moise, P.A., M.C. Birmingham, and J.J. Schentag, *Pharmacokinetics and metabolism of moxifloxacin.* Drugs Today (Barc), 2000. **36**(4): p. 229-44.
49. Soman, A., et al., *Concentrations of moxifloxacin in serum and pulmonary compartments following a single 400 mg oral dose in patients undergoing fibre-optic bronchoscopy.* J Antimicrob Chemother, 1999. **44**(6): p. 835-8.
50. Li, A.P., et al., *Effects of cytochrome P450 inducers on 17alpha-ethinyloestradiol (EE2) conjugation by primary human hepatocytes.* Br J Clin Pharmacol, 1999. **48**(5): p. 733-42.
51. Drusano, G.L., et al., *A population pharmacokinetic analysis of the penetration of the prostate by levofloxacin.* Antimicrob Agents Chemother, 2000. **44**(8): p. 2046-51.
52. *Drug-Resistant Tuberculosis: A Survival Guide for Clinicians, Second Edition*, ed. M. Ann M. Loeffler. 2008: Francis J. Curry National Tuberculosis Center.

Appendices

Appendix I: Informed Consent Form

12.1 Appendix I. Informed Consent Form

Research study title: Pharmacokinetics of Standard First and Second Line Anti-TB Drugs in the Lung and Lesions of Subjects Elected for Resection Surgery

Why is this study being done?

Tuberculosis (TB) is an infection of the lung that can be successfully treated by taking the proper medications for 6 months. However, some people get a more serious kind of TB (called multi-drug resistant TB, MDR-TB or extensively drug-resistant TB, XDR-TB) which are very difficult to treat and may not be cured by the regular medicines available. It is not completely known why sometimes the medication does not work. We think that some or all of the medication may not be getting to the place where the TB bacteria are.

Doctors at the Asan and National Medical Centers in Seoul, and Pusan National University Hospital in Busan, together with doctors and scientists from the Yonsei University and the National Institutes of Health (NIH) in the United States, have joined to find out if some of the common TB drugs are getting to the place where the TB bacteria are. Because you have elected to have part of your lung removed, you may be eligible for this study. If you agree to participate in this research study, we will give you one dose of 5 common TB drugs prior to your surgery. Once you have surgery, we will take some of your removed lung tissue and measure the amount of drug in different parts of your lung. This might help us to understand better how to use the drugs we have and how to design better drugs in the future.

Who is eligible for this study?

TB patients 20 years and above, who have decided to have lung resection surgery because of their hard to treat TB and are willing to give written or oral informed consent, are eligible for this study. We will have to be able to see your TB on a scan and make sure your heart is ok by doing an EKG. Those eligible will agree to the following: willing to have an MRI scan performed (described below); willing to substitute kanamycin for one dose of your drug regimen if an aminoglycoside (a type of drug) is in your regimen; also, in order to be on this study, you will have to agree that we can store your samples for future research. Portions of your medical record containing information collected prior to your enrollment into this study will be reviewed in order to ensure that you are eligible to participate in this protocol.

Participation in this research study is entirely voluntary. If you choose not to participate, you will still be able to receive the normal care you would otherwise be entitled to.

If you have an allergy to any of the five study drugs [isoniazid, rifampin, pyrazinamide, moxifloxacin or aminoglycosides (specific types of TB drugs)], any fluoroquinolone, rifamycin, or gadolinium, you will not be allowed on the study. Also, if you have kidney, liver or hearing impairment, you may not be able to be on the study. To be on the study, you must agree to use

contraceptives or not have sexual intercourse while on the study. If you are pregnant or breastfeeding, have severe gout, HIV, need to take certain medications, or have sever claustrophobia you will not be allowed on study. The investigator has the right exclude anyone with a condition he feels would warrant exclusion.

What will be done to me as part of this study?

If you participate in this study, you will continue taking the same drugs you are now taking. You will be given one dose of five drugs at one of 5 time points before your surgery, either 24, 12, 8, 4 or 2 hours before surgery. If you are presently on a drug that is designated an aminoglycoside, we will substitute that **one dose** of drug for the aminoglycoside in our study. The five drugs given will be rifampicin (RIF), isoniazid (INH), pyrazinamide (PZA), kanamycin, and moxifloxacin (MFX).

During surgery, the surgeon will take out part of your lung and some of the fluid around your lungs. After surgery, we will be given some of the fluid and a piece of the removed tissue as well as some information about your surgery. We will analyze the regions where TB live and also the lung tissue itself.

If you take part in this study, you will have the following tests:

1) A medical history. You will be asked questions about what kind of illnesses you may have had in the past, and what kind of medicines you may have taken for them.
2) Sputum samples. Sputum is the material you cough up which contains the TB germ. Hospital staff will test the TB germs in your sputum to find out if they are killed by the regular TB medicines used at your study hospital. Small amounts of sputum will be saved and analyzed to understand how the drug affects the germs. We will also take a sputum sample at the time of your surgery.
3) A clinical examination and medication history when you are enrolled.
4) Blood tests. You will also have about 2 mL of blood drawn from you 3-4 times during the course of the study, between the time of drug administration and the time of surgery. One more blood sample will also be collected during surgery. We will use this blood to measure levels of the different study drugs and other drugs you are taking.
5) If you are a woman of child bearing age, a urine sample is needed at the beginning of the study to make sure that you are not pregnant.
6) Urine. You will be asked to collect your urine from the time of your drug dosing to the time of your surgery. This is so we can look at the amount of drug you are excreting in your urine.
7) Special scan called ***a dynamic MRI (magnetic resonance imaging) scan.*** This type of scan uses magnetic and radio waves so there is no radiation exposure from this test. It takes between 30 and 60 min to complete. This is described in more detail in the next section. (What is an MRI scan?) You may be transported to another site in order to have your MRI scan.

<u>What is an MRI scan?</u>
The MRI scan is a special test that will give your doctor a better look at the TB in your lungs. This type of scan has the ability to look at almost all the tissue in the body and shows a clearer picture than a CT scan. Also, this scan does not involve any radiation. As part of your MRI you

will have an intravenous (IV) catheter placed to administer gadolinium, a contrast media that is an FDA approved medication used to improve MRI images. Most patients receiving gadolinium have no symptoms related to the injection of this medication. Mild symptoms that may occur include: coldness in the arm at injection, a metallic taste, headache, and nausea. MRI contrast agents may cause damage to the kidneys and soft tissue in certain patients with poor kidney function. However, you will not be given these agents if you have any of the conditions which might produce such adverse effects. An IV catheter is a small plastic tube that is inserted into a vein in your arm or hand using a small needle. This may result in discomfort at the site where the IV is placed. There is about a 1 % chance of discomfort due to injury to the vein during the injection. There is also a small risk of bleeding, blockage, inflammation (infection) of the vessel and surrounding tissues, or lightheadedness and weakness from this procedure. These complications are usually short lived. Permanent damage is extremely rare.

The MRI scanner is shaped like a big tube. You will be asked to lie very still on a table within the machine with your head on a soft cradle and your hands over your head. We will obtain several images for less than 5 minutes at a time. The total time required to complete the dynamic scan is 30 to 60 minutes. The machine will make banging noises while it takes the pictures. Because the scanner is shaped like a large cylinder (or tube), you may feel claustrophobic. If you are afraid that this might happen, please talk to your doctor ahead of time. If for any reason you feel that you cannot continue the scan, the scanning can be stopped and you can be removed from the scanner immediately.

How many people will take part in this study?

This study will involve fifteen (15) subjects. Each subject will be randomly assigned to one of the 5 dosing times.

How long will I be in the study?

If you agree to participate, you will be asked to stay on the study until 48 hours after your surgery. You can stop participating at any time. However, if you decide to stop participating, we encourage you to talk to your doctor first. The study may be terminated by the investigator, NIAID (as sponsor), or a regulatory agency without regard to your consent.

Stored Samples and Future Research

The samples taken will be stored for research. Samples taken at your study site will be stored at the International TB Research Center (ITRC). Some of the samples will be analyzed by investigators outside of ITRC and Yonsei and will be sent by an experienced courier service. These samples will help us learn more about how tuberculosis affects human cells, blood and other molecules. The research tests we will use are not like medical tests. Therefore we will not put the test results in your medical record; however, if you ask, someone on the study team will discuss the test results with you. You may decide at any time that you do not want your samples to be stored. If you choose for your samples not to be stored, please talk with your study doctor about your decision. This decision may or may not affect your participation in this protocol; however, it will not affect your medical treatment. You will be entitled to the same treatment as that given to other patients with your kind of infection if you can no longer participate in the protocol.

Labeling of Stored Samples

We will label your stored samples with a code that only the study team can link to you. Your information and samples will be stored under a study ID number and not your name. We will keep any information that can be traced back to you private to the extent permitted by law.

Future Studies

The study team will not send your samples to researchers outside the study team without an Institutional Review Board (IRB) review of any new research protocol that is requesting use of your stored samples. The IRB is a committee that oversees medical research studies to protect volunteers' rights and welfare.

Investigators will only use your samples for research. We will not sell them. Future research that uses your samples may lead to new products, but you will not receive payment for these products. Some future studies may need health information (such as smoking history or present health status) that we don't already have. If so, the study team will contact you for this information if you give us permission.

Genetic Testing

Some of the blood drawn or tissue from you as part of this study will be used for genetic tests. Genetic tests can help researchers study how health or illness is passed on to you by your parents or from you to your children. Some things to consider in thinking about whether or not to participate in these genetic studies include the possible effects on your emotional well being. In other words, how might you feel about yourself and your life if you learn information about risks that could affect your own health or that of your children? Also, relationships with other family members may be affected by finding out risks they have but did not want to know. An example would be if your children, brothers or sisters find out they have risks for health problems because of information found out about you.

It is our policy to not discuss such information with anyone unless it has direct medical or reproductive implications for you or your family. Any genetic information collected or discovered about you or your family will be confidential and will not be entered into your government healthcare center medical records. Research records containing this information will not contain your name, the data will be coded, and it will be kept under lock and key. Genetic information can be requested and obtained when a person applies for health insurance or a job and has signed a release. We will not release any information about you or your family to relatives, any insurance company, or employer unless you sign a release requesting us to do so.

What are the potential risks to me?

There are potential risks to you for participating in this study. There are minor risks related to drawing blood. You may feel some discomfort when the blood is drawn. You may also develop a small bruise or firm bump on your arm where the blood is drawn, and there is an extremely small risk that the skin around the blood draw may get infected.

There are potential side effects from the study drugs; however these are unlikely because you will only receive one dose of the drugs. Even with this small risk, the doctors treating you will still monitor you for possible events such as rash, flu-like symptoms, gastrointestinal upset, body fluids changing color, tingling feelings in your hands or feet, diarrhea, liver and kidney changes, headaches, a decrease in blood cells, blurry vision, sensitivity to light, drug-induced lupus, gout, pain, inability to sleep, restlessness, allergic reactions, increased liver enzymes and in extremely rare cases, a tear in your tendon(s) or a irregular heart rhythm.

There is a risk of serious and/or life-threatening side effects when non-study medications are taken with study drug(s). For your safety, you must tell the study doctor or nurse about all the medications you are taking before you start the study and also before taking any non-study medications while you are on the study. In addition, you must tell the study doctor or nurse before enrolling in any other clinical trials while you are on this study. The following medications are not allowed to be taken while on the study: antidepressants, warfarin, phenytoin, lithium, cimetidine, disulfiram, ergot derivatives, cholestyramine, fosphenytoin, carbamazepine, cyclosporine, tacrolimus, sirolimus, amiodarone, phenobarbital or levodopa.

What are the potential benefits to me?

There will be no benefit to you for participating in this study. We hope that the information learned from this study will benefit other patients with TB in Korea and throughout the world.

What other options are there?

Participation in this study is voluntary. Refusal to participate or deciding to discontinue participation will involve no penalty and will not affect your treatment in any way. If you decide not to participate your medical treatment will be the same as that given to other patients with your kind of infection.

What about privacy and confidentiality?

All personal information collected as part of this study will be kept strictly confidential. Any information about you that is linked to your name or other identifying information will be kept in a locked file cabinet that only study staff can access. After the study information about you is collected, it will be put together with information from all other study subjects without your name or other identifying information on it. No one other than the study staff, and governmental regulatory agencies (both in Korea and the US) who review and approve this research study, will be able to link you to the collected information.

When results of a research study are reported in medical journals or scientific meetings, the people who take part are not named or identified. In most cases, no information will be released about your involvement in the research study without your permission; however, if you sign a release of information form, for example, for an insurance company, the health center will give the insurance company information from your medical record. This information might affect (either favorably or unfavorably) the willingness of the insurance company to sell you insurance.

The Act on the Protection of Personal Information Maintained by Public Agencies and the Medical Services Act protect the confidentiality of your medical records. However, you should

know that the Acts allow release of some information from your medical records without your permission, for example, if it is required by the Korean Ministry of Food and Drug Safety, law enforcement officials, or other Korean regulatory agencies. In addition, monitors under contract to the National Institute of Allergy and Infectious Disease (NIAID) may have access to your research file in order to monitor all aspects of the study in accordance with the appropriate regulations.

By agreeing to participate in this study, you do not waive any rights that you have regarding access to and disclosure of your records. For further information on those rights, please contact the local Principal Investigator at your study hospital whose contact number and address is shown below.

What are the costs for me?

There is no cost to you for participating in this study. All study testing will be done free of charge to you. You will be given 325,000 won for your participation in the study. However, if the study is paying for your medical out-of-pocket expenses that you would normally pay yourself, you will not receive the 325,000 won. Any extra treatment related to side effects of the blood draw or of the study drugs will be provided by your study hospital.

What are my rights as a participant?

Taking part in this study is voluntary. You may choose not to take part or you may leave the study at any time. Leaving the study will not result in any penalty or loss of benefits to which you were otherwise entitled. We will tell you about new information that may affect your health, welfare, or willingness to stay in the study. You have the right to ask any questions you want about this study and about how the information will be used.

We would also like you to know that all investigators participating in this trial have received a copy of “The Guide to Preventing Financial and Non-Financial Conflicts of Interest in Human Subjects Research at NIH.” This is done because of NIH policy, and each institution will follow their own rules for avoiding conflicts of interest on this trial.

Whom do I call if I have any questions or problems?

Should injury related to the study occur during your participation in this study, immediate medical treatment will be offered to you from the hospital where you are participating at no charge. There will be no other monetary compensation given. Treatment may involve medications and/or hospitalization. For questions about the study or a research-related injury, contact the investigator at your study site.

For questions about your rights as a research participant, contact the IRB representative associated with your study site. The IRB is a group of people who review the research to protect your rights at your institution:

Asan Medical Center: Person in charge of ethical considerations

Pusan National University Hospital: Person in charge of ethical considerations
National Medical Center

If you would like to talk to another group whose goal is to protect research subjects, you can contact the Subjects Protection Center. These people are not connected to the study in any way.

A description of this clinical trial will be available on http://www.ClinicalTrials.gov, as required by U.S. Law. This Web site will not include information that can identify you. At most, the Website will include a summary of the results. You can search this Web site at anytime.

You will get a copy of this form. You can also ask for a copy of the protocol (full study plan).

I agree to take part in this study.

Name ____

Signature____ Date ________________________

Witness* ____ Date ________________________

* if subject is unable to read the consent

PI or Subinvestigator Signature________________________ Date ________________________
