# Supplementary material for: Tuberculosis drugs’ distribution and emergence of resistance in patient’s lung lesions: A mechanistic model and tool for regimen and dose optimization
Source: PLoS Med. 2019 Apr 2;16(4):e1002773. doi: 10.1371/journal.pmed.1002773 (PMC6445413; doi:10.1371/journal.pmed.1002773)
Supplement: S1 Table — (DOCX) [file pmed.1002773.s002.docx]

S1 Table: Number of observations for each lesion and drug

|  | Number of observations (number of patients) | | | | | | |
| --- | --- | --- | --- | --- | --- | --- | --- |
|  | RIF | INH | PZA | MFX | KAN | LZD | CFZ |
| Plasma | 54 (16) | 58 (16) | 59 (16) | 53 (16) | 49 (16) | 44 (9) | 12 (2) |
| Lung | 75 (16) | 71 (16) | 67 (15) | 71 (16) | 67 (15) | 41 (9) | 11 (2) |
| Cavity wall | 38 (11) | 36 (10) | 37 (10) | 37 (10) | 31 (9) | 24 (7) | 2 (2) |
| Small nodule | 16 (4) | 9.5 (3) | 10 (3) | 10 (3) | 8 (3) | 8 (3) | 9 (3) |
| Caseum from cavity | 36 (10) | 30.5 (10) | 31 (9) | 31 (9) | 23 (8) | 29 (9) | 7 (2) |
| Closed nodule caseum | 4 (3) | 4 (3) | 4 (3) | 4 (3) | 3 (2) | 4 (3) | 0 (0) |
| Caseous fibrotic nodule | 24 (4) | 14 (3) | 18 (3) | 18 (3) | 10 (3) | 9 (2) | 9 (2) |
| Necrotic nodule | 55 (12) | 55 (12) | 55 (12) | 55 (12) | 44 (11) | 37 (8) | 5 (2) |
| Fibrotic tissue | 8 (5) | 8 (5) | 8 (5) | 8 (5) | 8 (5) | 4 (2) | 1 (1) |
| Fungal ball | 4 (1) | 4 (1) | 4 (1) | 4 (1) | 4 (1) | 0 (0) | 0 (0) |

*Number of patients contributing to each lesion in parenthesis
