# Supplementary material for: Tuberculosis drugs’ distribution and emergence of resistance in patient’s lung lesions: A mechanistic model and tool for regimen and dose optimization
Source: PLoS Med. 2019 Apr 2;16(4):e1002773. doi: 10.1371/journal.pmed.1002773 (PMC6445413; doi:10.1371/journal.pmed.1002773)
Supplement: S2 Table — PK, pharmacokinetic (DOCX) [file pmed.1002773.s003.docx]

S2 Table: Plasma pharmacokinetic results compared to literature values

|  | Lesion model | Published values | Comments |
| --- | --- | --- | --- |
| RIF | 5.72 | 10.0 [1] |  |
|  |  | 7.76 [2] | after first dose |
|  |  | 8.67 [3] | non-parametric expectation maximization |
|  |  | 14.4 [4] | non-compartmental analysis at steady state |
|  |  | 14.5 [5] | non-compartmental analysis at steady state |
|  |  | 19.2 [6] | patients at steady state |
| INH | s: 9.41 | s: 9.7, |  |
|  | i: 24.0 | f: 21.6 [7] |  |
|  | f: 38.1 | 19.8 [5] |  |
|  |  | 25.8 [4] |  |
|  |  | s: 14.96 |  |
|  |  | f: 44.4 f [3] | non-parametric expectation maximization |
| PZA | 2.05 | 3.7 [3] | non-parametric expectation maximization |
|  |  | 4.5 [4] |  |
|  |  | 4.6 [5] |  |
| MFX | 8.93 | 7.9 [8] |  |
|  |  | 11.3 [9] |  |
|  |  | 11.6 [10] |  |
|  |  | 11.9 [5] |  |
|  |  | 12.9 [11] | intravenous dosing |
| CFZ | 16.3 | 4.18 [12] |  |
|  |  | 76.7  [13] |  |
| KAN | 4.61 | 3.75 [14] |  |
| LZD | 3.77 | 3.8 [15] |  |
|  |  | 4.8 [16] |  |
|  |  | 5.86 [17] |  |

References

1. Svensson R, Simonsson U. Application of the Multistate Tuberculosis Pharmacometric Model in Patients With Rifampicin-Treated Pulmonary Tuberculosis. CPT Pharmacometrics Syst Pharmacol. John Wiley & Sons, Ltd; 2016;5: 264–273. doi:10.1002/psp4.12079

2. Smythe W, Khandelwal A, Merle C, Rustomjee R, Gninafon M, Bocar Lo M, et al. A semimechanistic pharmacokinetic-enzyme turnover model for rifampin autoinduction in adult tuberculosis patients. Antimicrob Agents Chemother. American Society for Microbiology (ASM); 2012;56: 2091–8. doi:10.1128/AAC.05792-11

3. Peloquin CA, Jaresko GS, Yong CL, Keung AC, Bulpitt AE, Jelliffe RW. Population pharmacokinetic modeling of isoniazid, rifampin, and pyrazinamide. Antimicrob Agents Chemother. American Society for Microbiology Journals; 1997;41: 2670–9. doi:10.1128/AAC.41.12.2670

4. Tostmann A, Mtabho CM, Semvua HH, van den Boogaard J, Kibiki GS, Boeree MJ, et al. Pharmacokinetics of first-line tuberculosis drugs in Tanzanian patients. Antimicrob Agents Chemother. American Society for Microbiology Journals; 2013;57: 3208–13. doi:10.1128/AAC.02599-12

5. Magis-Escurra C, Later-Nijland HMJ, Alffenaar JWC, Broeders J, Burger DM, van Crevel R, et al. Population pharmacokinetics and limited sampling strategy for first-line tuberculosis drugs and moxifloxacin. Int J Antimicrob Agents. 2014;44: 229–234. doi:10.1016/j.ijantimicag.2014.04.019

6. Wilkins JJ, Savic RM, Karlsson MO, Langdon G, McIlleron H, Pillai G, et al. Population pharmacokinetics of rifampin in pulmonary tuberculosis patients, including a semimechanistic model to describe variable absorption. Antimicrob Agents Chemother. American Society for Microbiology (ASM); 2008;52: 2138–48. doi:10.1128/AAC.00461-07

7. Wilkins JJ, Langdon G, McIlleron H, Pillai G, Smith PJ, Simonsson USH. Variability in the population pharmacokinetics of isoniazid in South African tuberculosis patients. Br J Clin Pharmacol. 2011;72: 51–62. doi:10.1111/j.1365-2125.2011.03940.x

8. Dooley K, Flexner C, Hackman J, Peloquin CA, Nuermberger E, Chaisson RE, et al. Repeated Administration of High-Dose Intermittent Rifapentine Reduces Rifapentine and Moxifloxacin Plasma Concentrations. Antimicrob Agents Chemother. 2008;52: 4037–4042. doi:10.1128/AAC.00554-08

9. Kees MG, Schaeftlein A, Haeberle HA, Kees F, Kloft C, Heininger A. Population pharmacokinetics and pharmacodynamic evaluation of intravenous and enteral moxifloxacin in surgical intensive care unit patients. J Antimicrob Chemother. Oxford University Press; 2013;68: 1331–1337. doi:10.1093/jac/dkt040

10. Stass H, Kubitza D. Pharmacokinetics and elimination of moxifloxacin after oral and intravenous administration in man. J Antimicrob Chemother. 1999;43 Suppl B: 83–90. Available: http://www.ncbi.nlm.nih.gov/pubmed/10382880

11. Zhu L, Wang N, Yang W, Zhang Y, Zhao X, Ji S. Population pharmacokinetics of intravenous moxifloxacin 400 mg once-daily dosage in infected patients. J Infect Chemother. 2014;20: 621–626. doi:10.1016/j.jiac.2014.06.001

12. Sangana R, Gu H, Chun DY, Einolf HJ. Evaluation of Clinical Drug Interaction Potential of Clofazimine Using Static and Dynamic Modeling Approaches. Drug Metab Dispos. 2018;46: 26–32. doi:10.1124/dmd.117.077834

13. Nix DE, Adam RD, Auclair B, Krueger TS, Godo PG, Peloquin CA. Pharmacokinetics and relative bioavailability of clofazimine in relation to food, orange juice and antacid. Tuberculosis. Churchill Livingstone; 2004;84: 365–373. doi:10.1016/J.TUBE.2004.04.001

14. Chang MJ, Jin B, Chae J, Yun H, Kim ES, Lee YJ, et al. Population pharmacokinetics of moxifloxacin, cycloserine, p-aminosalicylic acid and kanamycin for the treatment of multi-drug-resistant tuberculosis. Int J Antimicrob Agents. Elsevier; 2017;49: 677–687. doi:10.1016/J.IJANTIMICAG.2017.01.024

15. Roger C, Muller L, Wallis SC, Louart B, Saissi G, Lipman J, et al. Population pharmacokinetics of linezolid in critically ill patients on renal replacement therapy: comparison of equal doses in continuous venovenous haemofiltration and continuous venovenous haemodiafiltration. J Antimicrob Chemother. Oxford University Press; 2016;71: 464–470. doi:10.1093/jac/dkv349

16. Dryden MS. Linezolid pharmacokinetics and pharmacodynamics in clinical treatment. J Antimicrob Chemother. Oxford University Press; 2011;66: iv7-iv15. doi:10.1093/jac/dkr072

17. Meagher AK, Forrest A, Rayner CR, Birmingham MC, Schentag JJ. Population pharmacokinetics of linezolid in patients treated in a compassionate-use program. Antimicrob Agents Chemother. American Society for Microbiology Journals; 2003;47: 548–53. doi:10.1128/AAC.47.2.548-553.2003
