# Supplementary material for: Tuberculosis drugs’ distribution and emergence of resistance in patient’s lung lesions: A mechanistic model and tool for regimen and dose optimization
Source: PLoS Med. 2019 Apr 2;16(4):e1002773. doi: 10.1371/journal.pmed.1002773 (PMC6445413; doi:10.1371/journal.pmed.1002773)
Supplement: S3 Table — KPL, rate parameter (DOCX) [file pmed.1002773.s004.docx]

S3 Table: Rate of drug moving from plasma to lesion (KPL) measured in (h^-1^)

| Drug | Lung | Necrotic nodule | Caseum closed nodule | Caseous fibrotic nodule | Caseum from cavity | Cavity wall | Fibrotic tissue | Small cellular nodule | Fungal ball |
| --- | --- | --- | --- | --- | --- | --- | --- | --- | --- |
| RIF | 1.68 | 0.751 | 0.367 | 0.367 | 0.204 | 1.98 | 0.209 | 4.67 | 0.212 |
| INH | 0.625 | 0.223 | 0.013 | 0.168 | 0.148 | 0.173 | 0.166 | 0.68 | 0.026 |
| PZA | 1.80 | 0.916 | 2.33 | 1.28 | 0.818 | 1.52 | 0.225 | 7.34 | 0.251 |
| MFX | 0.276 | 0.289 | 1.06 | 0.348 | 0.210 | 0.476 | 0.345 | 5.78 | 0.34 |
| CFZ | 4.32 | 4.77 | NA | 2.75 | 2.74 | 3.21 | 0.0113 | 2.86 | NA |
| KAN | 0.290 | 0.175 | 0.302 | 0.212 | 0.128 | 0.186 | 0.85 | 0.133 | 0.222 |
| LZD | 0.152 | 0.229 | 0.006 | 0.275 | 0.427 | 0.305 | 1.29 | 0.001 | NA |
