# Supplementary figures and images for: Tuberculosis drugs’ distribution and emergence of resistance in patient’s lung lesions: A mechanistic model and tool for regimen and dose optimization
Source: PLoS Med. 2019 Apr 2;16(4):e1002773. doi: 10.1371/journal.pmed.1002773 (PMC6445413; doi:10.1371/journal.pmed.1002773)

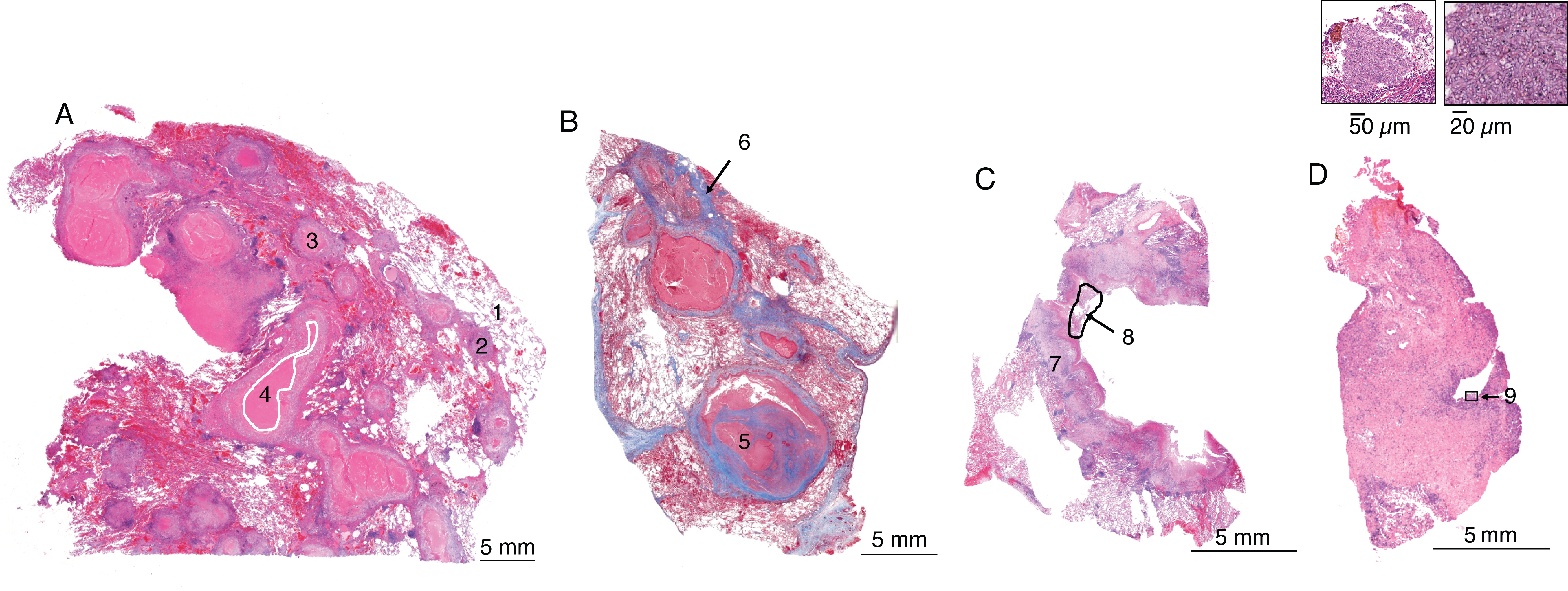

Supplement: S1 Fig — Samples were stained by HE and Mason’s trichrome for fibrosis. (A) HE staining of a lesion cluster including (1) uninvolved lung, (2) small cellular granuloma, (3) necrotic nodule, and (4) closed necrotic nodules with central caseum delineated by the white contour line, as indicated. (B) Mason’s trichrome staining of (5) large caseous (necrotic) fibrotic nodules and (6) highlighting of the fibrotic cuff in blue; (C) HE staining of a large cavity; most of the central caseum was lost upon sectioning. (7) Cavity wall delineates (8) remnants of caseum highlighted by the black contour lines. (D) HE staining of a collapsed cavity with fragments of a (9) fungal ball marked by black box and arrow. The hypha are shown in the inset above D. HE, hematoxylin–eosin. (TIF) [file pmed.1002773.s005.tif]

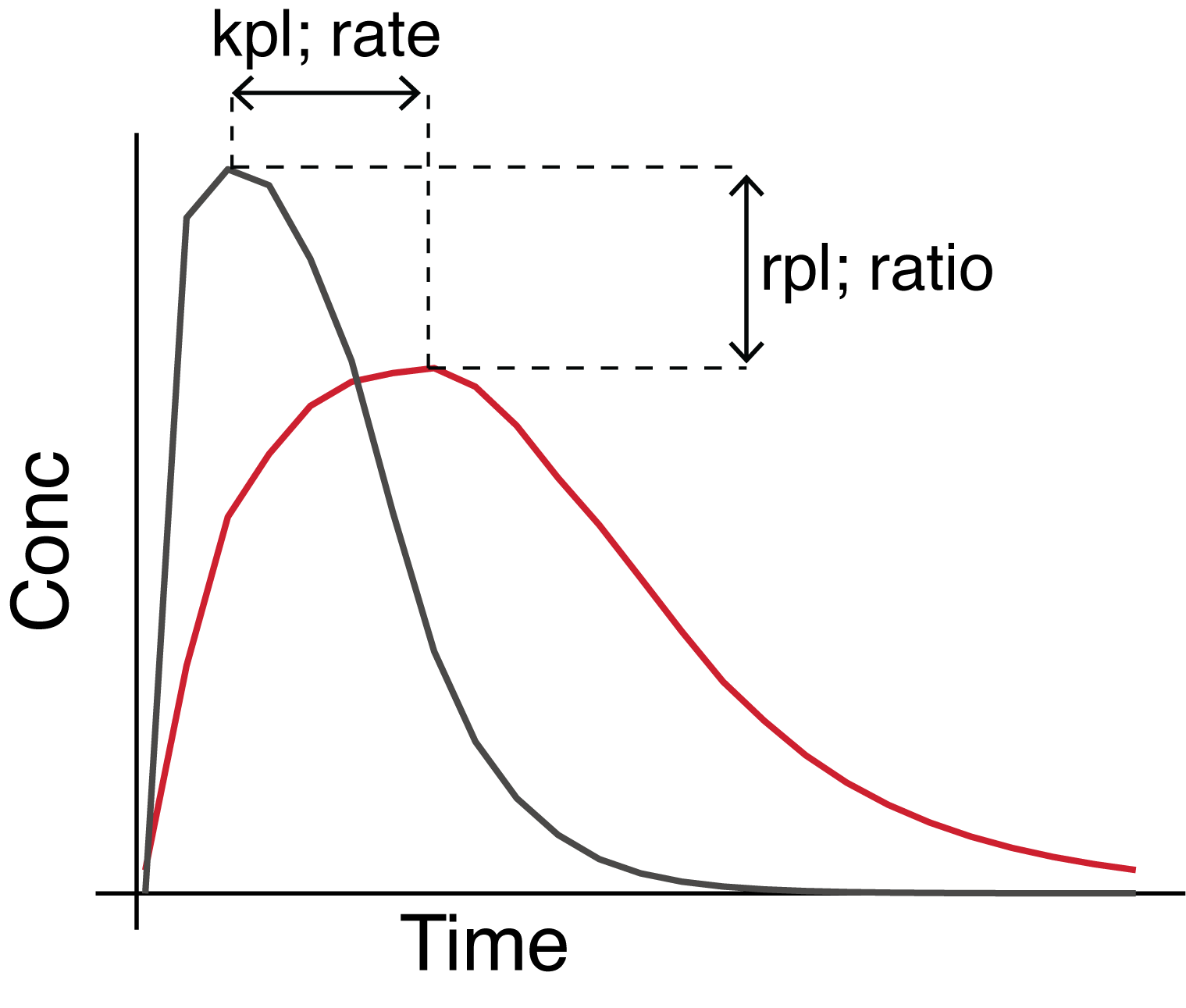

Supplement: S2 Fig — Plasma profile is shown in black and example of lesion profile shown in red. The KPL changes the profile on the time scale, while the RPL scales the concentration dimension of the profile. KPL, rate parameter; RPL, ratio parameter. (TIF) [file pmed.1002773.s006.tif]

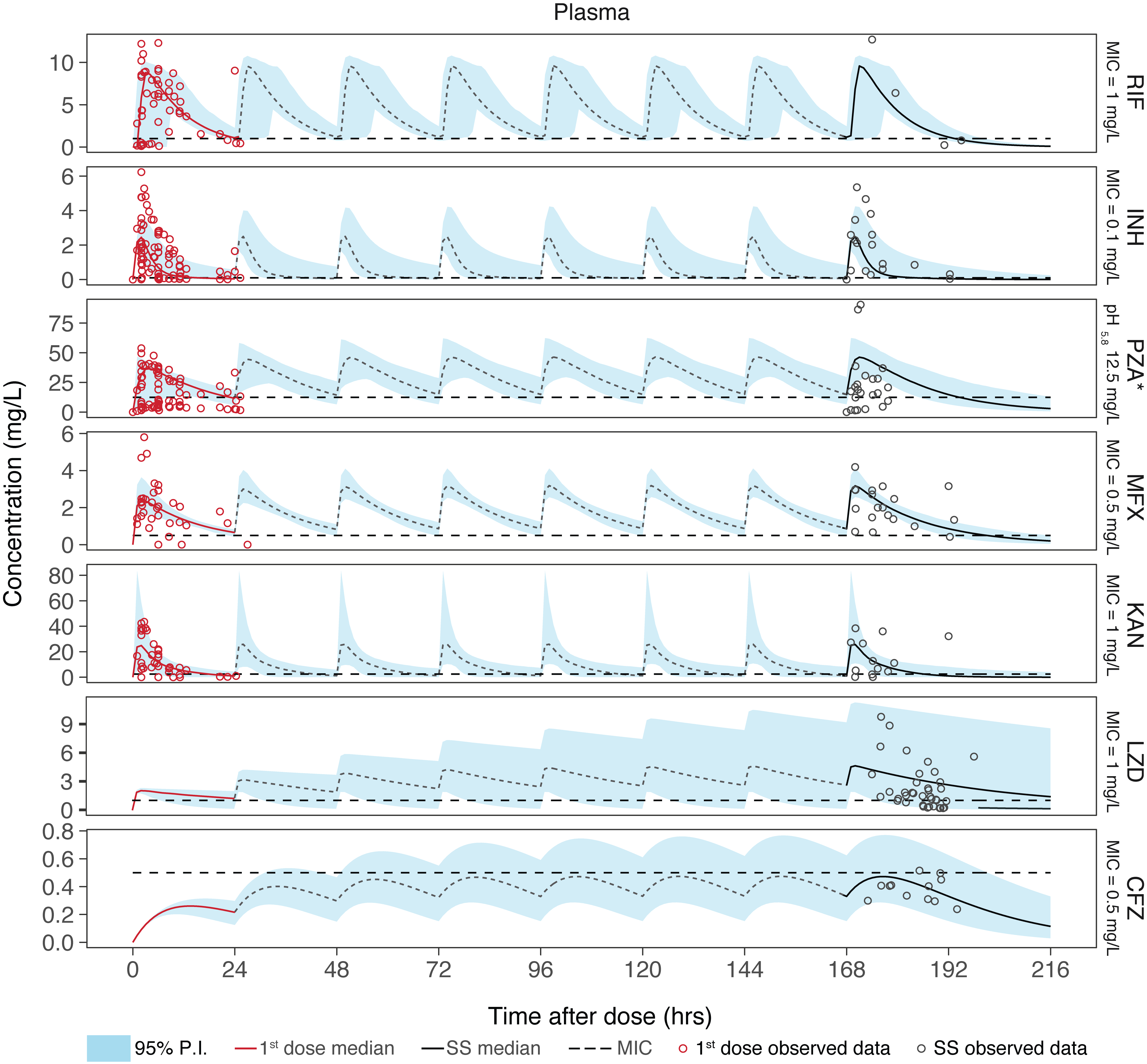

Supplement: S3 Fig — The visual predictive checks were simulated for 1,000 patients and are shown for all 7 drugs in plasma. The simulated concentration–time profiles are represented by a solid black line to represent the median at steady state, with the shaded light blue area representing the 95% prediction interval of the 1,000 patients simulated. The red line represents the median of plasma–time concentrations after the first dose of each respective drug. Open red circles represent drug concentrations of patients receiving their first doses, and black open circles represent observed data for patients who were at steady state at the time of resection. The dashed PK profiles serve to illustrate drug exposure increasing after each dose until reaching steady state. *PZA MIC of 12.5 mg/L is shown and is specific to an acidic environment less than pH 5.8. MIC, minimum inhibitory concentration; PK, pharmacokinetic; PZA, pyrazinamide. (TIF) [file pmed.1002773.s007.tif]

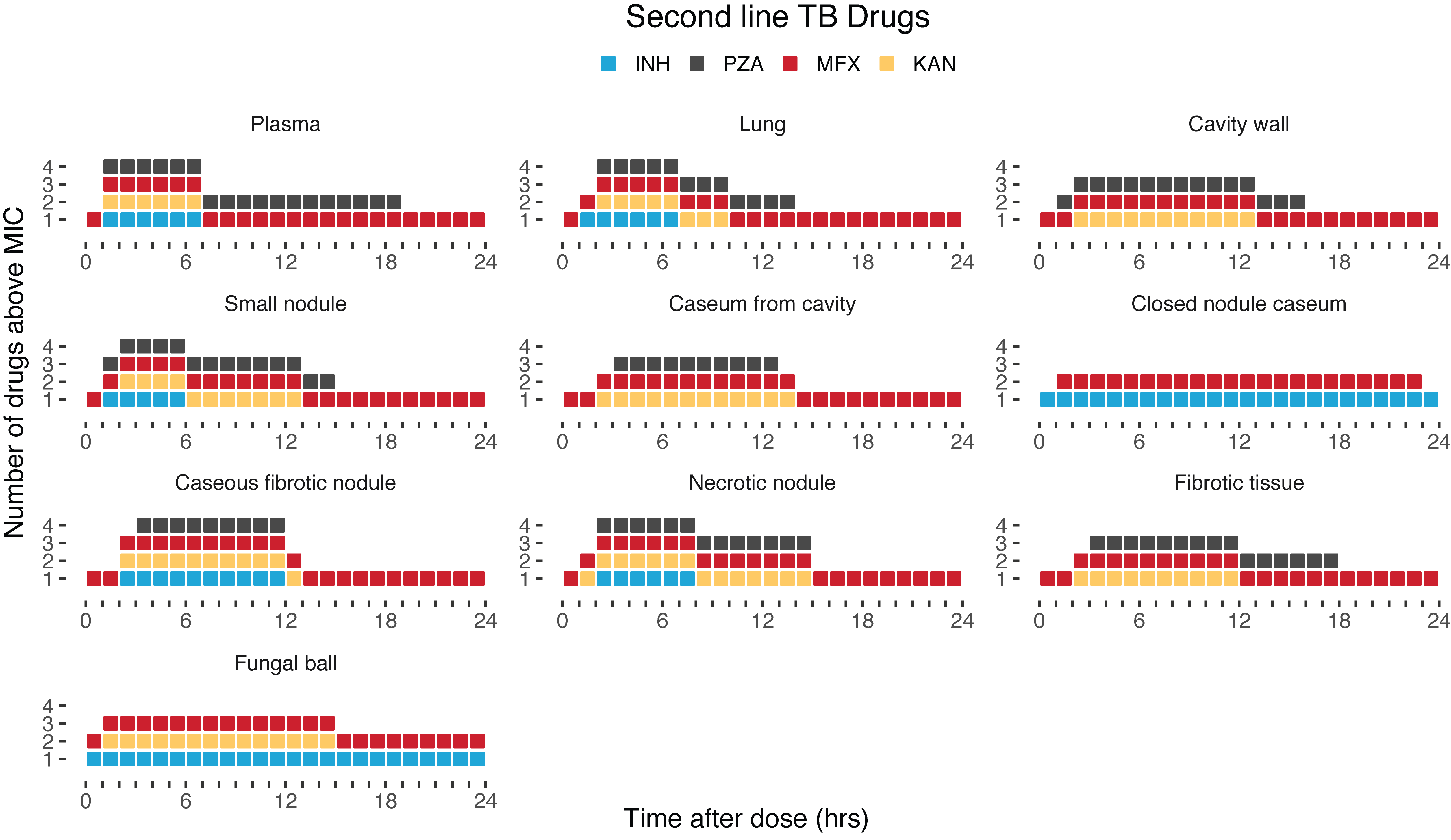

Supplement: S4 Fig — Second-line drugs at steady state collected as the fifth percentile (low-exposure or at-risk patients) of 1,000 simulated patients over the 24-h dosing interval. Each square represents 1 h that a drug is above the MIC. The drugs are stacked for each hour with different colors representing different drugs. MIC, minimum inhibitory concentration. (TIF) [file pmed.1002773.s008.tif]

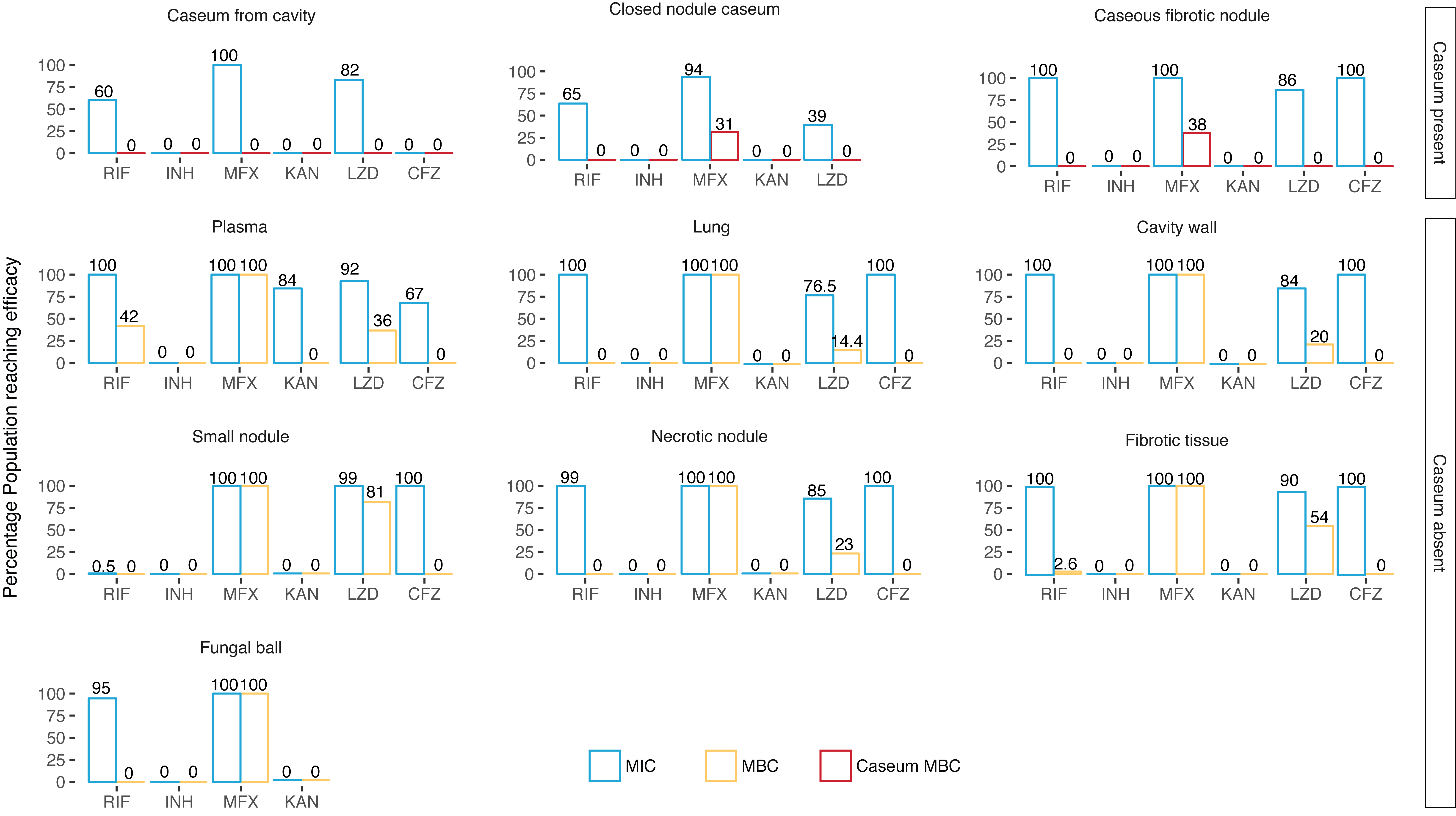

Supplement: S5 Fig — The percentage PK/PD efficacy target reached by a 1,000 simulated patient PK profiles as defined by their drug exposure versus drug MIC is shown in blue and MBC shown in yellow. Red boxes use CasMBC90 instead of MIC in the same set of simulated patients to compare that efficacy is both lesion and drug specific. CasMBC90, caseum-specific minimum bactericidal concentration; MIC, minimum inhibitory concentration; PK/PD, pharmacokinetic/pharmacodynamic. (TIF) [file pmed.1002773.s009.tif]
